# Chip-integrated metasurface full-Stokes polarimetric imaging sensor

**DOI:** 10.1038/s41377-023-01260-w

**Published:** 2023-09-06

**Authors:** Jiawei Zuo, Jing Bai, Shinhyuk Choi, Ali Basiri, Xiahui Chen, Chao Wang, Yu Yao

**Affiliations:** 1 School of Electrical, Computer and Energy Engineering, Arizona State University, 85281 Tempe, AZ USA; 2 Center for Photonic Innovation, Arizona State University, 85281 Tempe, AZ USA; 3 Biodesign Center for Molecular Design and Biomimetics, Arizona State University, 85281 Tempe, AZ USA

**Keywords:** Optoelectronic devices and components, Imaging and sensing, Nanophotonics and plasmonics

## Abstract

Polarimetric imaging has a wide range of applications for uncovering features invisible to human eyes and conventional imaging sensors. Chip-integrated, fast, cost-effective, and accurate full-Stokes polarimetric imaging sensors are highly desirable in many applications, which, however, remain elusive due to fundamental material limitations. Here we present a chip-integrated Metasurface-based Full-Stokes Polarimetric Imaging sensor (MetaPolarIm) realized by integrating an ultrathin (~600 nm) metasurface polarization filter array (MPFA) onto a visible imaging sensor with CMOS compatible fabrication processes. The MPFA is featured with broadband dielectric-metal hybrid chiral metasurfaces and double-layer nanograting polarizers. This chip-integrated polarimetric imaging sensor enables single-shot full-Stokes imaging (speed limited by the CMOS imager) with the most compact form factor, records high measurement accuracy, dual-color operation (green and red) and a field of view up to 40 degrees. MetaPolarIm holds great promise to enable transformative applications in autonomous vision, industry inspection, space exploration, medical imaging and diagnosis.

## Abstract (web summary)

We present a chip-integrated Metasurface-based Full-Stokes Polarimetric Imaging sensor (MetaPolarIm) with ultra-compactness, record high measurement accuracy, dual color operation, and a field of view up to 40 degrees.

## Graphical abstract

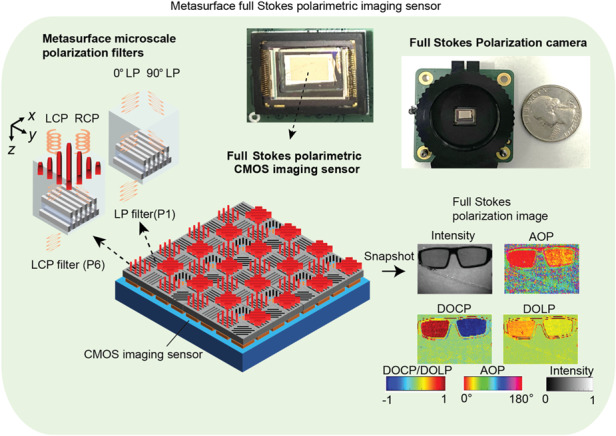

## Introduction

The development of imaging systems has profoundly impacted our lives, from smartphone cameras to the most advanced medical imaging equipment and even to space exploration. Besides intensity and color, the polarization state of light, which can change upon emission, scattering, or transmission by an object, is essential for various applications such as target detection^1–3^, biomedical diagnostics^4–6^, remote sensing^7^, defense^8^, and astronomy^9^, etc. Thus, it is highly desirable to create a compact and economical polarimetric imaging system that not only records the light intensity but also analyzes the polarization state at each pixel to provide more complete information about the target object.

Conventional polarimetric imaging systems require complex optical components and moving parts, making system miniaturization difficult^10–12^. Moreover, these systems suffer from reduced frame rates and inaccurate extracted polarization information due to motion in the scene. Monolithic integrated linear polarization imaging sensors have been demonstrated by integrating metallic nanowire-based linear polarization filter arrays onto CMOS imaging sensors^13–16^ based on a spatial division measurement approach to avoid moving parts; they are ultra-compact and high-speed, yet cannot perform complete measurement of polarization states due to the lack of materials with strong chirality for ultra-thin circular polarization filters. Recently, various types of chip-integrated polarimetric imaging sensors equipped with circular polarization filters have been explored, including liquid crystal polymers^17–19^, birefringent polymers^20,21^, and micro retarders on metallic nanowires^22^. However, these thin-film structures post various limitations when integrating with silicon imaging sensors due to material compatibility issues, environmental stability issues, and potential crosstalk issues due to the relatively thick films (5μm) required for sufficient polarization filter extinction ratios^19^ (See Table S1 for a summary of the state-of-art polarimetric imaging sensors).

Recent development in optical metasurfaces and metamaterials show promising progress toward much more compact, flexible, and robust solutions for polarization detection than conventional techniques^23^. Prevailing techniques for metasurface-based full Stokes polarimetric imaging can be categorized into dielectric metasurface diffraction grating^24,25^, microscale polarization metalens array^26,27^, and plasmonic metasurface^28^. Among them, the dielectric metasurface diffraction grating^24,25^ devices are efficient, yet the field of view is limited to less than 10° (to avoid significant cross talk)^24^ and operation bandwidth is limited to 10 nm^24^ or less than 2 nm^25^ due to the dispersive nature of diffraction gratings. The microscale polarization metalens array^26,27^ exhibits high efficiency and high compactness. However, the operation bandwidth is limited to less than 10 nm^26,27^ due to the dispersive nature of metalens in polarization control and focus. Moreover, the optical crosstalk becomes more pronounced as pixel size decreases (less than 7.5% for pixel size of 7 μm and 13% for pixel size of 2.4 μm)^26^ due to optical diffraction. The plasmonic metasurface microscale polarization filter arrays^28^ based on spatial division principles exhibited high measurement accuracy, and broad bandwidth, and can be integrated on chip, yet the working wavelength is limited to infrared wavelength due to high plasmonic loss in visible wavelengths. (See Table S2 for a summary of the state-of-art metasurface-based polarimetric imaging sensors). Besides metasurface-based polarimetric imagers, many single point polarimetric detectors have also been demonstrated. Among them, dielectric diffraction gratings^29^ have achieved ultra-compactness and high efficiency in visible, yet the incidence angle range and operation bandwidth are still small due to their angular and chromatically dispersive nature. Graphene-plasmonic hybrid based on spatial division^30^ and division of time^31^ and plasmonic metasurfaces based on spatial division^32–35^ has achieved broader working wavelengths^33,34^, high detection accuracy^33^, on-chip integration with photodetector^30–33,35^ for infrared wavelengths. However, the employment of plasmonic structures in the visible range generally remains challenging due to the high optically loss of plasmonic structures. Metal-dielectric hybrid metasurfaces^36^ have been used for chiral metasurfaces and polarization detection with high efficiency and high performance for near IR wavelengths^36^, yet similar designs for visible range post challenges for nanofabrication (See Table S3 for a summary of the state-of-art polarimetric imaging sensors).

For many potential applications of polarimetric imaging sensors, from machine vision to space exploration^37–39^, chip-integrated imaging devices are highly desirable due to their ultra-compactness and mechanical robustness. So far, the demonstration of chip-integrated metasurface-based Full-Stokes polarimetric imaging sensors for visible wavelengths remains elusive. Polarimetric imaging sensors based on dielectric metasurface diffraction grating require at least a millimeter-scale propagation distance and an optical lens between the metasurface and the imaging sensor^24,25^, thus fundamentally not suitable for on-chip integration. Polarimetric imaging devices based on microscale polarization metalens arrays require tens of micrometers propagation distances between the microlens array and the imaging sensor^26,27^, which post fundamental challenges for chip-integration, especially for large-scale imaging sensors with high pixel density due to such large spacing and potential optical cross-talk^26^. Thus, in our search for a proper design strategy to implement chip-integrated full-Stokes polarimetric imaging sensors at visible wavelengths, we focused on transmission-type metasurface micro-polarization filter arrays, which could be directly integrated onto CMOS imaging sensors for high-speed single-shot polarimetric imaging, with high polarization measurement accuracy, high pixel density, large field of view, minimal crosstalk, and suitable for scalable nanomanufacturing method.

Here, we report the demonstration of a chip-integrated single-shot Metasurface-based Full-Stokes CMOS Polarimetric Imaging sensor (MetaPolarIm), which is featured with ultra-compactness, high speed, high detection accuracy, large field of view, broad bandwidth and feasibility for large scale fabrication. The MetaPolarIm is composed of a subwavelength-thick hybrid metasurface polarization filter array (MPFA) integrated into a CMOS imaging sensor. We demonstrated CMOS-compatible broadband LP and CP filters with high polarization extinction ratios based on double-layer metallic gratings and hybrid chiral metasurfaces, respectively. The metasurface designs exhibit large alignment error tolerance and are suitable for large-scale fabrication. Theoretical analysis suggests that our MPFA design can maintain low crosstalk (<1%) and high polarization measurement accuracy as micro-polarization-filter pixel size decreases to 1 μm, which makes ultra-high resolution polarization imaging sensors possible. The MetaPolarIm sensor adopted the spatial division measurement approach^40^ to obtain full Stokes polarimetric images at one snapshot with imaging speed ultimately limited by the CMOS imaging sensor. Compared to other methods based on polarization splitting^24–27^ or division of time^31^, this spatial division approach based on chip-integrated MPFA sacrifices optical intensity but allows simultaneously the simplest device configuration, highest speed (single-shot measurement), best mechanical stability, and most compact device configuration. In the experiment, our MetaPolarIm sensor achieved measurement errors for all Stokes parameters <2% for red and green at normal incidence. Its field of view is up to 40 degrees with Stokes parameter measurement error <4% for red color. Our demonstration proved a viable path to implement chip-integrated full-Stokes polarimetric imaging sensors at visible wavelengths based on metasurface device concepts, which are promising to enable transformative applications of ultra-compact high speed full-Stokes polarimetric imaging systems in various applications, such as autonomous vision^38^, space exploration^39^, medical imaging and diagnosis^4–6^ and industry inspection^41^.

## Results

### Design concept

**Fig. 1:**
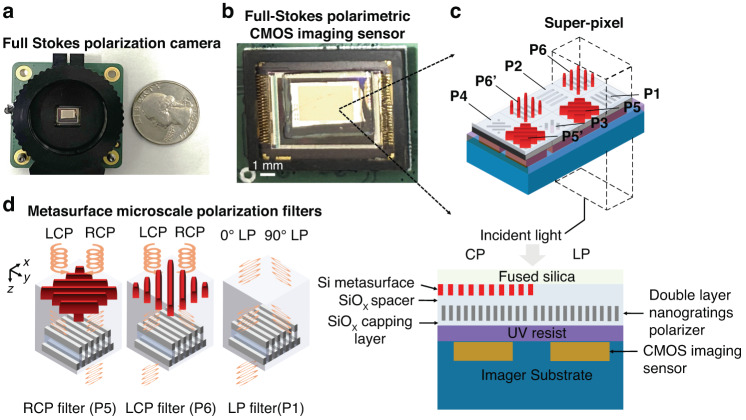
CMOS integrated full Stokes polarimetric imager with dual operation wavelength. **a** Image of a full Stokes polarization camera beside a U.S. quarter dollar coin (lens not attached). **b** Image of full Stokes polarimetric CMOS imaging sensor **c** Top: 3D Conceptual illustration of chip integrated full Stokes CMOS polarimetric imaging sensor. Here P1-P4 denotes the LP filters with transmission axes at 0°, 90°, 45°, 135° respectively. P5, P5’ and P6, P6’ denote chiral metasurface filters transmitting right-handed circularly polarized (RCP) and left-handed circularly polarized (LCP), respectively. Here, P5 and P5’, P6 and P6’ are identical in dimensions respectively. bottom: 2D cross-section of the chip-integrated polarimetric imaging sensor. **d** 3D conceptual illustration of a pair of chiral metasurfaces responsible for transmitting RCP and LCP light, respectively (P5, P6), and a LP filter (P1)

The chip integrated MetaPolarIm (Fig. 1a) comprises an MPFA and a commercial CMOS imaging sensor beneath it, as shown in Fig. 1b, and is almost the same size as the conventional CMOS imaging sensor. The MPFA consists of over 75,000 microscale polarization filters. Each super-pixel has two pairs of circular polarization (CP) filters (P_5_ and P_6_), and four linear polarization (LP) filters (P_1_ to P_4_). Each polarization filter is defined as one sub-pixel and integrated on top of one or a few imaging pixels of the CMOS imaging sensor underneath (Fig. 1c). The polarization state at each super-pixel can be obtained by the measurement results of a combination of LP and CP sub-pixels and their corresponding instrument matrix^33^, as discussed in detail in Materials and Methods. Figure 1d shows the schematics of the CP and LP filter designs, which are based on metal-dielectric hybrid metasurfaces. Both LP and CP polarization filters have a thickness of less than a wavelength, resulting in a highly compact form factor for the demonstrated full-Stokes polarimetric imaging camera.

**Fig. 2:**
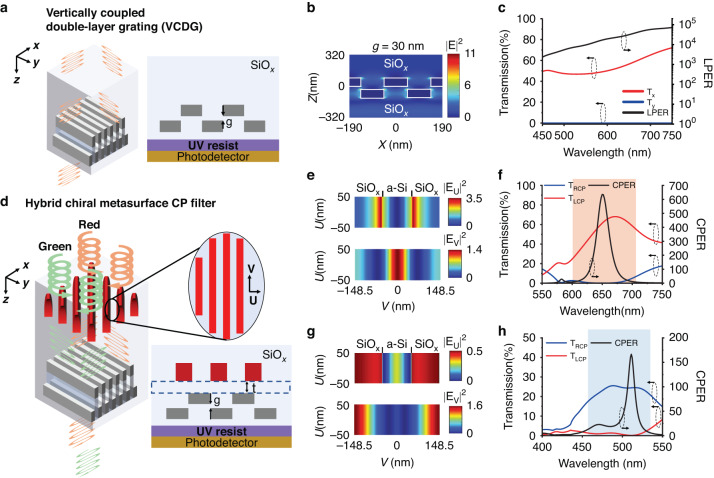
Design principle and optical simulation of the chiral metasurface and VCDG for CP and LP detection. **a**–**h** Design of the chiral metasurface and VCDG for CP and LP detection (**a**) 3D schematic and 2D cross-section of VCDG. The thickness(*t*_*Al*_) of Aluminum (Al), width(*w*_*Al*_), period(*p*_*1*_), and the vertical gap (*g*) of VCDG are optimized to be *t*_*Al*_ = 80 nm, *p*_*1*_ = 190 nm, *w*_*Al*_ = 95 nm and *g* = 30 nm, respectively. **b** Cross-sectional view of VCDG near field distribution with input light (650 nm) polarized along x axis. **c** Transmission spectra and LPER of VCDG with input light polarized along x and y axis respectively. **d** 3D schematic and 2D cross-sectional view of the top layer Si nanograting and bottom layer VCDG, respectively. The thickness (*t*_*si*_), period (*p*_*Si*_), width (*w*_*Si*_), and tilted angle (*θ*_*Si*_) of Si nanograting are *t*_*si*_ = 130 nm, *p*_*Si*_ = 297 nm, *w*_*Si*_ = 100 nm and *θ*_*Si*_ = 6° respectively. The thickness (*t*_*Al*_) of Aluminum (Al), period (*p*_*2*_), and vertical gap (*g*) of bottom layer VCDG are optimized *t*_*Al*_ = 80 nm, *p*_*2*_ = 210 nm, and *g* = 30 nm, respectively. The thickness of the SiO_*x*_ spacer layer is *t* = 400 nm. **e** Near field distribution of the Si grating when incident polarization is along the width of the Si nanogratings (U axis) and length of the Si nanogratings (V axis) at 629 nm. **f** Simulated transmission spectra(left) and CPER (right) of dielectric-metal hybrid chiral metasurface at 550 nm-750 nm. The red shadow region indicates the wavelength range for CPER over 10. **g** Near field distribution of the Si grating when incident polarization is along the U and V axes at 500 nm. **h** Simulated transmission spectra(left) and CPER (right) of dielectric-metal hybrid chiral metasurface at 400 nm-550 nm. The blue shadow region indicates the wavelength range for CPER over 10

The metasurface-based microscale polarization filters were all made of CMOS compatible materials, i.e., Aluminum (Al), silicon, and silicon oxide. We chose vertically coupled double-layered gratings (VCDGs) made of Al at subwavelength scale (Fig. 2a) as the LP filter because of its high linear polarization extinction ratio (LPER) and fabrication simplicity. For incident light with electric field vector oriented in parallel with the nanogratings (i.e., y-axis in Fig. 2a), it is almost completely reflected by the double layers of gratings. For incident light with an electric field vector oriented vertically to the nanogratings (x-axis in Fig. 2a), it can couple effectively into the gap plasmon modes in the deep subwavelength-scale vertical nanogap (g < λ_0_/10) between the top and bottom Al nanowires and then transmit through the grating layer with high efficiency, as shown in Fig. 2b. The small vertical gap between the two coupled grating layers is essential for high transmission and LPER. We have optimized the design parameters of the VCDG, including the vertical nanogap, grating duty cycle, period, and Al thickness to maximize the LPER and transmission efficiency over the visible wavelength range (Fig. S1). Our designed VCDG has LPER over 1000 in the visible range with maximum efficiency of 56% and 47% at 650 nm and 510 nm, respectively, as shown in Fig. 2c. Compared to single-layered metallic grating linear polarizers of the same grating thickness^13^, the VCDGs has 2 order of magnitude higher LPER and slightly lower transmission efficiency for the transmitted polarization (Fig. S2). Moreover, the fabrication process of VCDGs is much simpler and has a higher success rate, since it does not require Al metal etching and lift-off of Al metal films with a thickness of a few hundred nanometers, which are very challenging for nanofabrication of feature size down to tens of nanometers.

We designed the CP filters based on metal-dielectric hybrid chiral metasurfaces by integrating a Silicon metasurface quarter-wave plate (QWP) onto the metallic (Al) VCDG linear polarizer. In a previous work, we designed Si nanopillars array as metasurface QWP to achieve high-efficiency CP filters for NIR wavelength^36^. Yet, here for shorter wavelengths in visible spectral region, we engineered artificial optical birefringence (Fig. 2d) utilizing Si nanogratings with much more feasible dimensions for nanofabrication and broadband operation bandwidth. Compared with nanopillars, silicon nanograting metasurfaces exhibit a larger artificial birefringence Δ*n* ($\varDelta n={n}_{V}-{n}_{U}$, where ${n}_{V}\,,{n}_{U}$ are defined as the effective refractive index along nanograting direction and its orthogonal direction), therefore requiring a much smaller height-to-width aspect ratio (~1.3 according to Fig. S3) for the same $\frac{\pi }{2}$ phase difference engineering than that of Si nanopillar design (~4 according to Fig. S4). The large artificial birefringence of Si nanogratings stems from the anisotropic near-field distribution under different incident light polarization (illustrated in Fig. 2e, a cross-sectional view in Fig. S5). When incident light is polarized along the U axis (fast axis), the Si gratings mainly support leaky modes^42–44^, and the electric field intensity is highly localized in SiO_*x*_ gaps between Si (Fig. S5a). On the other hand, for incident light polarized along the V axis (slow axis), the Si nanogratings support modes with an electric field mostly located inside Si (Fig. S5b). Therefore, the effective refractive index along the U axis is lower than that along V axis. The CP filters were realized by integrating an aluminum VCDG linear polarizer with its optical axis oriented at +/-45 degrees (for LCP/RCP filters) to that of the silicon metasurface QWP. In the LCP filter design (red in Fig. 2d), the LCP incident light is converted into LP light oriented along x axis by the Si nanogratings and then passes through the VCDGs with high transmission, while the RCP incident light is converted into LP light oriented along y axis and blocked by the VCDGs, resulting in very low transmission (<1%). Moreover, we also demonstrated that by engineering the dispersion of silicon nanograting metasurfaces, one can achieve CP filters simultaneously for two wavelength ranges. By properly adjusting the silicon nanograting period, duty cycle, and thickness, we achieved a phase difference between fast and slow axes to be $\frac{\pi }{2}$ at red color (629 nm, Fig. S6). Meanwhile, Si nanogratings acts as QWP at a shorter wavelength centered around 500 nm (green color), mainly due to its highly dispersive near-field distribution (Fig. 2g, cross-sectional near-field distribution in Fig. S7), and therefore produce an advanced phase $\frac{3}{2}\pi$ along the fast axis (Fig. S6). In this case, LCP incident light around 500 nm is converted into LP light polarized along y axis and then blocked by VCDGs, while RCP incident light is converted into LP light polarized along x axis and transmits through the VCDGs (green in Fig. 2d). Figure 2f shows the transmission spectra (left axis) and the circular polarization extinction ratio (CPER, right axis) of an LCP filter for red color 550 ~ 750 nm, obtained by full wave simulation. The device has a maximum CPER of over 600 with a transmission efficiency of 61.5% at 650 nm. Moreover, the device design can operate from 600 nm to 700 nm with CPER over 10 (shadowed in red). For shorter wavelength around 500 nm, the chiral metasurface detects opposite CP handedness at a shorter wavelength, as shown in Fig. 2h. The chiral metasurface exhibits a maximum CPER of 170 with a transmission efficiency of 24.5% at 510 nm and 450 nm to 525 nm with CPER over 10 (shadowed in blue). The dual working wavelength of Si nanograting broadened the operation wavelength range with a total bandwidth of 175 nm (CPER > 10), which is one order higher than other grating diffraction-based designs^24,26^.

We have also studied the impact of finite microscale polarization filter size on device performance because the truncation was found to affect metasurface properties^45^. Our simulation results suggest that the truncation decreases the polarization extinction ratios of the metasurface polarization filters (Figs. S8 and S9). Nonetheless, the degradation of the polarization extinction ratio becomes very significant for filter lateral length (square-shaped filter) smaller than 1.5 μm. For the chiral metasurface microscale CP filters, the minimal lateral dimensions can be as small as 0.945 × 0.945 µm^2^, to maintain CPER > 100 and transmission efficiency over 60% in red color (Fig. S8). This makes our MPFA design suitable for polarization imaging sensors with high pixel density.

### Device fabrication and characterization

**Fig. 3:**
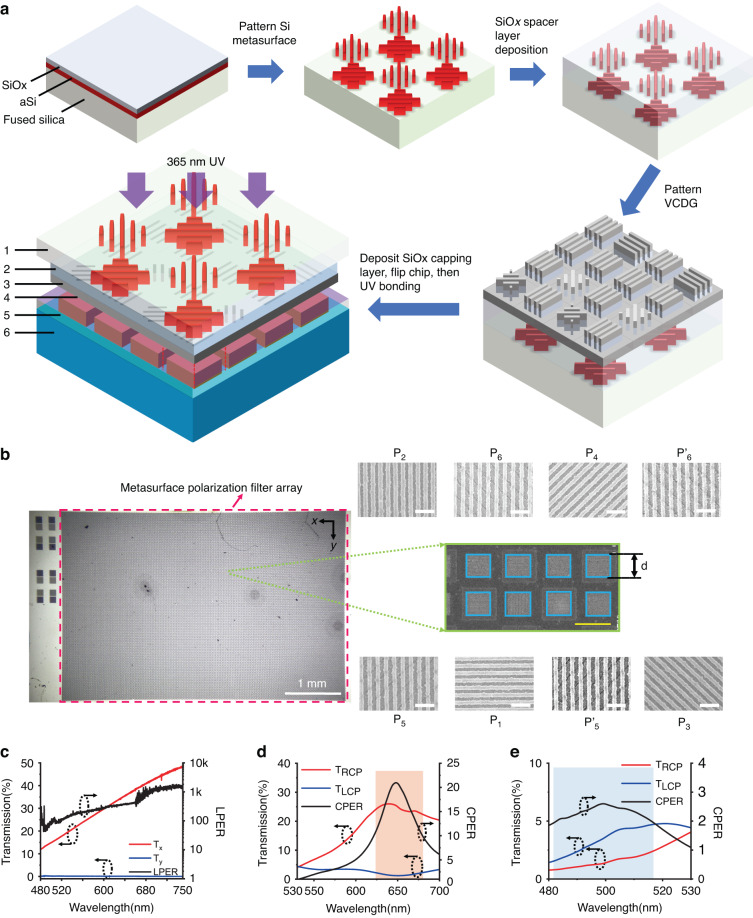
Device fabrication and characterization. **a**–**e** Schematic of device fabrication and experimental characterization**. a** Schematic of the device fabrication process. 1: fused silica wafer, 2: SiO_*x*_ spacer layer, 3: VCDG,4: SiO_*x*_ capping layer, 5: spin coated ultra-violet (UV) resist onto the CMOS imaging sensor, 6: CMOS imaging sensor. **b** The microscopic photograph(left) and the SEM image of one super pixel among the fabricated MPFA (right), scale bar:10 μm. 90°,45°,0°,135° LCP, and RCP on each sub-Fig indicate the polarization state each metasurface filter transmits. Scale bar: 500 nm (white). The size of CP and LP micro-filters are 6.2 µm by 6.2 µm, with spacing 4.65 µm between adjacent filters to minimize crosstalk. **c** Measured transmission and LPER of fabricated VCDG. **d**, **e** Measured transmission and CPER of the chiral metasurface at 530–700 nm and 480 nm-530 nm, respectively. Legend T_RCP_, T_LCP_ indicate the transmission ratio at their correspondent input CP handedness. Red shadow region indicates the wavelength range for CPER over 10. Blue shadow region indicates the wavelength range for CPER over 2

Figure 3a shows the fabrication process flow of the MPFA. First, amorphous silicon (*a-*Si) and SiO_*x*_ were deposited onto fused silica wafers by plasma-enhanced chemical vapor deposition (PECVD), followed by electron-beam lithography (EBL), lift-off, reactive-ion etching (RIE) of SiO_*x*_ mask, and inductively coupled plasma etching (ICP) of *a-*Si to form Si nanogratings (SEM image and detailed dimension of fabricated Si nanogratings are included in Supplementary Information, Fig. S10). Then, a dielectric spacer layer (520 nm) of SiO_*x*_ was sputtered onto Si nanogratings, followed by EBL patterning and Cr deposition for the VCDG layer. After lift-off, the formed Cr nanogratings were used as a hard mask to transfer the grating pattern into the SiO_*x*_ spacer layer (depth 110 nm) by reactive ion etching (RIE). Then 80 nm Aluminum (Al) was deposited by electron beam evaporation to form the VCDG structure. Such a bi-layer metasurface fabrication scheme is fundamentally compatible with conventional CMOS manufacturing. In fact, our VCDG fabrication eliminates Al dry etching, thus greatly reducing the processing constraint. Further, it is also possible to further avoid the use of a metal liftoff process when metal contamination is a concern by lithographically patterning in a metal-free resist stack^45^ as a mask in both the Si QWP and VCDG fabrication. In addition, the use of nanogratings in the metasurface geometry designs also simplify the nanolithography and pattern transfer processes, both in conventional CMOS manufacturing and other scalable nanomanufacturing such as nanoimprint lithography. Finally, the fabricated MPFA was covered with 200nm-thick sputtered SiO_*x*_ as a protection layer and was cut into 4 mm * 3 mm pieces using a wafer dicing saw and bonded onto a CMOS imaging sensor (Sony IMX 477) using a home-built UV bonding setup. The schematic of the UV bonding setup is included in Supplementary Information (Fig. S11), and more detailed information about the fabrication process and UV bonding process is discussed in the Materials and methods section. The fabricated MPFA (Fig. 3b) consists of over 75.2 K microscale metasurface polarization filters (MMPF), with a total area of 3.65 × 2.43 mm^2^. The scanning electron microscope (SEM) images of one super pixel (center) and 8 different MMPFs, i.e., subpixels, are shown in Fig. 3b. Detailed dimensions of each subpixel fabricated are included in Supplementary information (Table S4). The duty cycle of fabricated VCDGs is between 46% to 55% and is dependent on the orientations of the gratings due to slightly different EBL writing results at different writing angles.

We performed full wave simulation using FDTD to further investigate the dependence of crosstalk on the pixel size of our device (see Fig. S12a for the definition of pixel size in our investigation). When the polarization filter is fabricated directly on the Si photodiode (*t*_*s*_ = 0, see Fig. S12d). Our analysis suggests that 1μm pixel size can ensure optical crosstalk of less than 1% (Fig. S12b). The minimum required pixel size increases with the gap size between the polarization filter and photodiode due to increased optical diffraction (Fig. S12c). For example, a minimum of 3.3 μm (Al frame size included) is required to ensure crosstalk less than 1% for *t*_*s*_ = 700 nm. (Fig. S12d). We used metal frames around each MMPF to reduce the optical cross talk between the adjacent pixels to less than 1%. Here the metal frame (width 4.65 μm) is chosen to minimize the amount of oblique incidence light, which can have a serious impact given a thick spacer layer (around 700 nm, including bonding resist and SiO_*x*_ capping layer) between the MPFA and CMOS imaging sensor and absence of micro lens array on the metasurface array, the metal frame width could be further reduced by directly fabricating the MPFA on Si photo diode and/or adding micro lens array to minimize crosstalk between adjacent pixels.

The performance of fabricated MPFA was first characterized by a visible spectrometer with a broadband LP polarizer with LPER over 1000 and a broadband QWP. (See more details of the setup in the Materials and methods section and Fig. S13 in Supplementary Information). Figure 3c shows the measured transmission spectra of the VCDG (oriented at 0°) patterned on the SiO_*x*_ spacer layer without the Si metasurface buried underneath, which shows an efficiency of 35% and LPER over 400 around 650 nm. Noticeably the VCDG provides LPERs from 100 ~ 400 from 520 nm to 750 nm, offering broadband LP detection with high accuracy, which is one order higher than single layered gratings presented in literature^13^. Compared with simulation results, the transmission efficiency and LPER of fabricated VCDG are still lower. We attributed such performance degradation to the surface roughness of sputtered SiO_*x*_ spacer layer (*Ra* = 8.43 nm, as shown in Fig. S14) as well as the surface and edge roughness introduced in the metal evaporation. As a comparison, we fabricated VCDG on a fused silica wafer with similar dimensions by EBL patterning followed by Al E-beam evaporation. We increased the Al deposition rate and vacuum level to improve Al film quality. The results showed reduced surface and edge roughness of the fabricated VCDG (Fig. S15a). The measured transmission efficiency was also improved by almost twice across the visible wavelengths (results also shown in Fig. S15d). Thus, we expect to improve the VCDG linear polarizer performance by improving the quality of the SiO_*x*_ spacer and Al thin film.

Figure 3d shows the measured circular dichroism (CD) spectra of a CP filter that transmits RCP light while rejecting LCP light at wavelengths around 650 nm. The measurement wavelength range was limited to 480 nm to 700 nm due to the limitations of the experimental setup. This CP filter provides CPERs of more than ten over a wavelength range of 60 nm (shadowed in red), about 10% of the optimal operation wavelength at 650 nm. Figure 3e shows the measured circular dichroism (CD) spectra of the same device at shorter wavelengths around 500 nm. As expected in the simulation, the CP filter transmits LCP light while rejecting RCP light with a CPER of ~2.5 at 500 nm (the region with CPER > 2 is shadowed in blue color). Compared with simulation results, the chiral metasurface-based CP filters showed significant degradation in performance. Besides the surface and edge roughness that led to lowered efficiency and LEPR of the VCDG structure, the Si nanogratings buried under the spacer layer leave an increased surface roughness (*R* = 28.3 nm, as shown in Fig. S16) of the SiO_*x*_ spacer layer, resulting in a significant decrease in LPER and thereby reducing the CPER of the chiral metasurface. We measured the LPER (Fig. S19) of the bottom layer VCDG of a chiral metasurface (device B) with similar dimensions (Fig. S17). The LPER of the bottom layer VCDG is only ~15. As a result, the CPER of device B is reduced to below 10 (Fig. S18). Thus, we expect to increase the device’s optical performance experimentally by reducing the surface roughness of the spacer layer via surface planarization. One possible method is using chemical-mechanical polishing (CMP) for planarization, yet CMP is challenging for nanoscale structures because it potentially introduces local defects. We plan to address surface planarization issues for future integration of multi-layer metasurface devices. Furthermore, we also investigated the alignment errors between top Si gratings and bottom VCDGs and their impacts on the device performance. Alignment angle deviation of ±1° from the target (*θ* = ±45°) lead to a slight CPER decrease (<4.5% for red, Fig. S20a and <10% for green, Fig. S20b). To keep CPER above 80% of the design, an alignment deviation within ±2° should be realized. From the SEM images of the fabricated MPFA, we estimated an alignment error of 0.5° from the target (*θ* = ±45°) (Fig. S21). This angle deviation has a negligible impact on CPER (<0.1%) of the chiral metasurface (Fig. S20c) while the efficiency remains the same (Fig. S20d). In addition, the translational displacement between Si nanogratings and VCDGs also only slightly decreases CPER of the chiral metasurface design by <5.3% (Fig. S22). Based on experiment observations and theoretical calculations, here we conclude that our designed chiral metasurface is robust to fabrication alignment errors.

### High-accuracy polarimetric detection

**Fig. 4:**
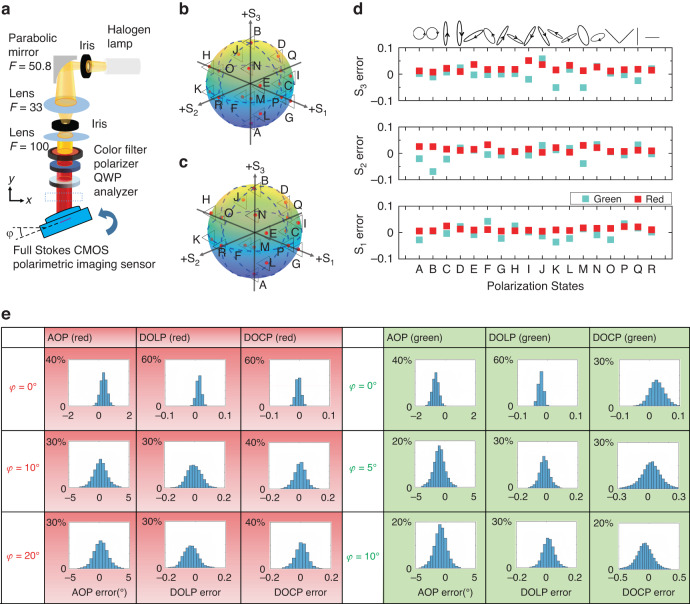
High Accuracy full Stokes polarization detection. **a**–**e** Full Stokes polarization detection in dual color and different incidence angles. **a** A schematic of the customized experimental setup for generating arbitrary polarization states for full Stokes polarization detection, φ denotes the camera rotation in azimuth angle for oblique incidence measurement. Here parabolic mirror, 2 iris and 2 lens were applied to generate a collimated beam with divergence angle of less than 0.5°, unit: mm. **b**, **c** Illustration of measured polarization state (Red dot) and its input reference (Triangle) distribution on Poincaré sphere in red and green color, respectively. **d** Error distribution for Red/Green color full Stokes parameter detection measurement result. **e** AOP, DOLP_,_ and DOCP detection error distributions of all MPFA pixels for polarization state D at normal incidence and oblique incidence of red color (left) and green color (right). In all the plots, x-axes represent the errors, and y axes represent the corresponding percentage of pixels

The simplest method for obtaining the Stokes parameters in the spatial division method is directly subtracting the intensity of 0°,90°,45°,135° LP, and RCP and LCP components^46^. Yet the accuracy is limited by the LPER and CPER of the polarization filters and their wavelength dependence^47^. Here, we applied bandpass filters of 630–670 nm, 480–520 nm to demonstrate the best measurement accuracy of polarization state for red and green colors, respectively. We first performed calibration to obtain the instrument matrix *A* of the polarimetric imaging sensor. Then the Stokes parameters *S* can be obtained using the linear equation: $S={A}^{-1}I$, where *I* denote the intensity vector of the polarimetric imaging sensor. (See Supplementary Information section 5 for more details about the Instrument matrix calibration process). This method can greatly increase polarization detection accuracy^33,48^. Here, the calibration process is not dependent on the imaging target as the purpose of the calibration process is to obtain the instrument matrix of a super-pixel, i.e., the first row of the Mueller matrix of each individual metasurface polarization filter element. Besides, the calibration process, which is expected to take a half hour, can be finished quicker (less than 2 min) if the rotation of optical components can be done by motorized motors. Figure 4a show a customized optical setup for calibrating the device. A color-filtered, uniform, collimated beam with sufficient spot size is incident onto the full Stokes polarization imaging sensor, which is mounted onto a rotational stage to control the light incidence angle *φ*. During the calibration, *φ* is kept at 0° (normal incidence) to obtain the instrument matrix for each microscale polarization filter. We apply a polarization state generator (polarizer and QWP) to generate arbitrary polarization states. With each polarization state input, a snapshot of the transmitted light intensity through MPFA was taken to obtain the intensity vector^48^. The instrument matrix *A* of MPFA could be readily obtained after sufficient polarization state input to form an intensity matrix. Besides, a spatially over sampling approach was applied during the instrument matrix calculation to enhance the imaging resolution (see Fig. S23 in Supplementary Information for more details).

After calibration, we performed full-Stokes polarization detection on the same setup at different incidence angles, using the instrument matrix *A* calibrated at *φ* = 0°. Note that we did not perform the instrument matrix calibration at different incident angles because in most imaging applications, the imaging sensor will receive light from different incident angles all at once and usually could not distinguish light from different angles.

We first generate 18 arbitrary polarization states and measure them using a conventional PSA (see Materials and Methods), then the generated polarization states are incident onto the device uniformly. Figure 4b, c depicts the measured Stokes parameter ${S}_{i}^{j}$ and their reference values ${{S}_{R}}_{i}^{j}$ measured by polarization state analyzer (PSA) (*i* = *1,2,3; j* = *A, B… R*) on the Poincaré sphere under red and green color input respectively. In total, we chose 18 reference polarization states sparsely distributed in all eight quadrants of the Poincaré sphere to verify the full Stokes polarimetric detection accuracy better. Here ${S}_{i}^{j}$ is averaged out over the entire MPFA : ${S}_{i}^{j}=\tfrac{\mathop{\sum }\nolimits_{a=1,b=1}^{n,p}{{S}_{i}^{j}}_{a,b}/{{S}_{0}^{j}}_{a,b}}{n\times P}$ (*i* = 1, 2, 3, *j* = 1,2… 18, *n* = 335, *p* = 221). where ${{S}_{i}^{j}}_{a,b}/{{S}_{0}^{j}}_{a,b}$ represents normalized Stokes parameters measured by each pixel. Figure 4d shows the measurement error ${\varDelta S}_{i}^{j}$ for each polarization states at *φ* = 0° under red and green colors. Here ${\varDelta S}_{i}^{j}$ is defined as: ${\varDelta S}_{i}^{j}={S}_{i}^{j}-{{S}_{R}}_{i}^{j}$ (*i* = 1, 2, 3, *j* = 1,2… 18). The mean absolute error $({\rm{MAE}})\tfrac{{\sum }_{j=1,}^{18}|\varDelta {S}_{i}^{j}|}{18}$ (*i* = 1,2,3) for S_1_, S_2_, S_3_ are 1.84 %, 1.93 %, 1.79% for green and 1.03%, 1.43%, 1.99% for red, respectively. Measured ${\varDelta S}_{i}^{j}$ of other incidence angles is included in Supplementary Information (Fig. S24 for red and Fig. S25 for green color). Based on the Stokes parameters measured, we calculated the angle of polarization (${\rm{AOP}}=\frac{1}{2}{\rm{arctan }}\frac{{{\rm{S}}}_{2}}{{{\rm{S}}}_{1}}$), degree of circular polarization (DOCP = S_3_/S_0_), and degree of linear polarization (${\rm{DOLP}}=\sqrt{{{\rm{S}}}_{1}^{2}+{{\rm{S}}}_{2}^{2}}/{{\rm{S}}}_{0}$). Here ${\sigma }_{i}$ is written as: ${\sigma }_{i}=\tfrac{{\sum }_{j}^{18}{\sigma }_{i}^{j}}{18}$ (*i* = 1,2,3, *j* = 1,2…18) where ${\sigma }_{i}^{j}$ is defined as the standard deviation of Stokes parameter measurement error of MPFA, denoted as: ${\sigma }_{i}^{j}=\sqrt{\tfrac{\mathop{\sum }\nolimits_{a=1,b=1}^{n,p}{\left({{S}_{i}^{j}}_{a,b}/{{S}_{0}^{j}}_{a,b}-{{S}_{R}}_{i}^{j}\right)}^{2}}{n\times p}}$. Measured MAE and averaged standard deviation *σ*_*i*_ for S_1_, S_2_, S_3_, AOP, DOLP, and DOCP at different incidence angles is included in Supplementary Information (see Table S5 for red color and Table S6 for green color measurement results respectively). MAE for AOP, DOLP, and DOCP are 0.26%,1.41%,1.99% for red and 0.78°,1.26%, 1.79% for green at *φ* = 0°. When the incident angle *φ* is within ±20°, MAE for S_1_, S_2_, S_3_ can maintain less than 4% for the red color. For *φ* = ±30°, MAE for S_3_ increases to 17.51%, this is because CPER of chiral metasurface reduces by two orders compared to normal incidence, indicated by FDTD simulation (Fig. S26a). Similarly, the device has an MAE of less than 4.1% for green color input with an incidence angle within ±5°, and with an incidence angle up to ±10°, the detection error of S3 increases to 15% due to a decrease of CPER (Fig. S26b). Here we evaluate the field of view for a polarization imaging sensor with the full angle range, i.e., twice the incident angle, for full Stokes parameter measurement with a reasonably small measurement error (<5%). Our device can achieve a field of view over 40° for red color and only 10° for green color.

We then evaluated the uniformity of detection accuracy across the polarimetric imaging sensor. Figure 4e shows the distribution of measurement error of AOP, DOCP, and DOLP for polarization state D at different incidence angles. Over 90% of the polarimetric imaging pixels provide small measurement errors for DOLP (<2%), DOCP (<2%), and AOP (0.5°), respectively, for both red and green color at normal incidence. A larger AOP, DOCP, and DOLP error range can be seen when the incidence angle increases. When *φ* is within ±20°, over 80% AOP, DOCP, and DOLP error range are less than 10%, 10%, 2°. The increase of the non-uniformity of measurement errors at oblique incidence is majorly due to the instrument matrix being calibrated at *φ* = 0° (normal incidence), causing calibration errors when the imaging sensor is measuring polarization states in oblique incidence as MPFA has different CPER upon oblique incidence (Fig. S26). Measurement error distribution of other polarization states under the red/green input can be found in Supplementary Information session 7 (Figs. S27 to S44).

So far, we have shown that with the instrument matrix method, our full Stokes polarimetric imager allows highly accurate polarization state detection at red and green colors with a single snapshot. Moreover, we have shown that the instrument matrix method can be applied to an array of micro polarization filters with a standard deviation of less than 1% for both red and green color under normal incidence and less than 5% for red color within ±20° blique incidence. Our polarimetric imager, together with the instrument matrix reconstruction method, has the potential to achieve high accuracy full Stokes parameter imaging with dual-wavelength coverage. Note that we applied bandpass filters of 630–670 nm, 480–520 nm to demonstrate the best measurement accuracy with S_1_, S_2_, S_3_ measurement errors less than 2%. The measured CPER was still above 5 (Fig. 3d) over the wavelength range from 600 nm to 700 nm. In the shorter wavelength region, the measured CPER was close to 2 (Fig. 3e) from 460 nm to 530 nm. The LPER of the linear polarization filter was over 80 (Fig. 3c) from 460 nm to 700 nm. Therefore, we expect the sensor array can still perform full Stokes polarization detection for a broader wavelength range from 600 nm to 700 nm and from 460 nm to 530 nm at the cost of increased measurement errors. According to simulation results presented in Fig. 2c, f, and h, if we can improve the fabrication process to obtain better performance for the CP filters, the operational wavelength range can be further extended.

### Dual-wavelength full-Stokes parameter imaging

**Fig. 5:**
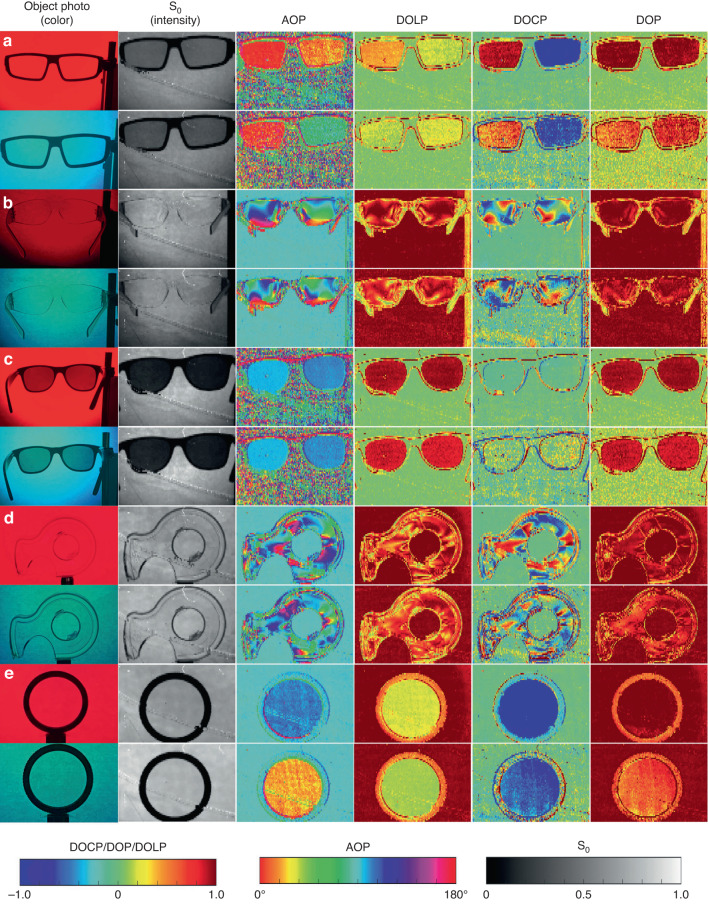
Full Stokes polarization images of various objects. Full Stokes polarization imaging of objects carrying polarization information. **a** Full Stokes polarization image of a 3D glasses with the unpolarized background. **b** Full Stokes polarization image of a Plastic goggles with LP input as background. **c** Full Stokes polarization image of a pair of Sunglasses with unpolarized input as background. **d** Full Stokes polarization image of a Plastic cage, with LP input as background. **e** Full Stokes polarization image of a camera CP filter, with LP input as background. In each case, an object photo was taken by a cell phone camera. “Color” image indicates Bandpass filters applied, 630 nm–670 nm (Red) and 480 nm–520 nm (Green) in front of the light source

Here we demonstrate the full Stokes polarization imagery with several objects for proof of concept. The experimental setup for full Stokes polarization imaging is shown in Supplementary Information (Fig. S45). A 40-nm bandpass filter centered at 650 nm and 500 nm was applied separately in front of the mercury lamp fiber. We took polarization images of several real-life objects carrying the polarization information. The objects were positioned behind the paper allowing diffused light to transmit from behind. Each image section includes an object photo taken by a cell phone camera; an image with colorful background identifying the color; the raw exposure S_0_; the angle of polarization (AOP); the degree of linear polarization (DOLP); the degree of circular polarization (DOCP) and the degree of polarization (DOP). Next, we discuss those sections respectively. Figure 5a shows a pair of 3D glasses consisting of opposite CP information. The handedness of the input CP cannot be seen in the sample photo and intensity image but is clearly shown in the DOCP image. We notice that the values of DOCP and AOP of the right glasses are different when taken with red and green colors, indicating differences in the transmitted polarization state of 3D glasses under a different color. This example reveals potential of MetaPolarIm in virtual reality and augmented reality technology where CP glasses are widely used^49^. Figure 5b shows a pair of plastic goggles. In the sample photo, the plastic goggle looks transparent. However, the DOCP image of goggles looks rather un-uniform because of the birefringence of plastic stemming from stress. In addition, the goggles’ DOCP image under the red and green color light input shows a readily different distribution, indicating plastics’ birefringence dependence on the input color. This example clearly exemplifies the advantage of full Stokes polarization imaging under dual operation wavelengths, which could be applied to numerous applications such as industry imaging and remote sensing. Figure 5c examines the polarization information of sunglasses. The red and green DOLP images and DOP images both show high values, while the DOCP value of the glasses region is nearly 0, indicating the unpolarized light gets linearly polarized upon transmission through the sunglasses. Both red color and green color images show similar conclusions, revealing the broadband linearly polarized characteristics of the sunglasses. This example reveals the potential of MetaPolarIm in applications such as glare reduction and contrast enhancing of objects with polarization information. Figure 5d depicts a plastic cage imaged with LP as the input background. High DOCP values and un-uniform AOP image in the cage area are due to the inner stress of the plastic material upon molding, giving rise to birefringent material optical characteristics. We notice that the red and green color DOCP are readily different, which reveals differences in the inner stress distribution of the plastic cage under different colors. This example reveals the potential of MetaPolarIm in applications in material stress detection. Figure 5e examines a simple test; the LCP camera filter is circularly polarized; this is not visible to the traditional imaging sensor but is clearly shown in red and green color DOCP images. Besides, thanks to dual operation wavelength, our sensor shows that the AOP of the CP filter is readily different in red and green color, indicating the light transmitted through the LCP filter with different wavelengths shows different polarization states.

## Discussion

In this work, we designed and fabricated metasurface-based microscale polarization filter array composed of broadband microscale linear polarization filters and dual-color (green and red) circular polarization filters (75.2 K pixels). We then chip-integrated the MPFA onto the CMOS sensor and calibrated the sensor polarization detection with the instrument matrix calibration method. With calibration, we achieved high Stokes measurement accuracy: Averaged measurement Error of less than 2% for S_1,_ S_2_ S_3_ in red and green color. Moreover, our polarimetric imaging sensor can maintain an error of less than 5% up to ±20° oblique incidence for red color and ±5° for green color. Finally, we demonstrated the full Stokes polarimetric imaging in real-life objects invisible to the traditional imaging sensor in red and green color with total operation bandwidth of 80 nm. From the polarization images of objects, we find polarization information carried by these objects is color-dependent, revealing the advantage of dual-wavelength operation. Overall, the MetaPolarIm is featured with high speed (single-shot measurement), superior mechanical stability, ultra-compact footprint, fabrication scalability, and CMOS compatibility. Our demonstration proved a viable path to implement chip-integrated full-Stokes polarimetric imaging sensors at visible wavelengths based on metasurface device concepts, which could be widely applied in various real-world applications, such as autonomous vision, industrial inspection, space exploration, and biomedical imaging.

## Materials and methods

### Simulations

FDTD simulations from Lumerical Inc. FDTD solver were applied to calculate the transmission efficiency, CPER of the chiral metasurface, and LPER of double-layer gratings. The real optical material refractive index of *a*-Si and Aluminum measured by UV-NIR spectroscopic ellipsometry (J.A. Woollam, M-2000) was applied to FDTD material explorer to calculate the device performance precisely. Specifically, for the simulation of double-layer gratings, the plane wave along the grating width and grating length direction was applied to calculate the LPER and efficiency of double-layer gratings. For the chiral metasurface, a tilt angle of 6° of the Si grating obtained from SEM images was considered in the simulations. Two orthogonally linearly polarized plane waves with a phase difference of ±*π*∕2 were super positioned to represent LCP/RCP light input, respectively. In all FDTD simulations, we simulate one unit cell and apply periodic boundary conditions along the in-plane direction. The simulation convergence auto shut-off level was set to 10^−5^. The mesh size was set to 2 nm for higher simulation accuracy. For oblique incidence, we use the BFAST plane wave type to maintain the oblique incidence angle the same for all wavelengths.

### Fabrication

1. Si nanograting: Fused silica wafer was cleaned by RCA-1 cleaning, then amorphous silicon (α-Si) of 130 nm was deposited by plasma-enhanced chemical vapor deposition (PECVD) (Oxford PlasmaLab 100, 350 °C/ 15 W) on fused silica wafer, followed by deposition of 60 nm SiO_*x*_ (350 °C/ 20 W) without breaking vacuum as a hard mask layer. 10 nm Cr layer was then deposited by thermally evaporating (Denton benchtop turbo) as the discharge layer during 1st EBL exposure. Double-layer polymethyl methacrylate (PMMA) resists (70 nm 2.5% 495k followed with 50 nm 2% 950k) were coated, followed by 2-min post-baking at 180 °C. A pattern composed of 168 by 56 super-pixels array was written with a JEOL JBX-6000FS EBL machine working at 50 keV with a current of 500 pA. After exposure, the sample was developed for 2 min. The developer is a mixture of methyl isobutyl ketone (MIBK) and isopropanol (IPA) with a mixing ratio of 1:3. Next, the sample is cleaned with 30 s of Oxygen plasma (PIE Scientific Plasma cleaner, immersion mode O_2_ 10 sccm /20 W) to remove PMMA residue on the exposure region. Next, 3 nm Cr adhesive layer and 12 nm SiO_*x*_ were deposited by electron beam evaporating (lesker #3). Cr and SiO_*x*_ were lifted off by soaking in warm acetone (60 °C) for more than 12 h, followed by acetone gun cleaning. After the lift-off process, a SiO_*x*_ nanostructures array was formed, which masked Cr discharge layer etching by Reactive Ion Etching (RIE) (OXFORD PLASMALAB 80PLUS, Cl_2_/O_2_: 9/3 sccm, 10 mTorr, D.C. bias/power 18 V/70 W). An isolated Cr/ SiO_*x*_ layered nanostructure mask were thus formed, which then masked anisotropic etching of 60 nm SiO_*x*_ hard mask by RIE (Plasma-Therm RIE 790, CHF_3_/ O_2_ 40/3 sccm, 40 mTorr, 250 W). The dry etching of SiO_*x*_ stopped at the α-Si layer; during the dry etching procedure, 12 nm SiO_*x*_ on SiO_*x*_/Cr layered structure was consumed. Then the Cr was removed by CR-4s (Greentek) etchant, and the α-Si layer was etched by ICP-RIE (ICP/bias power of 250/140 W, 10 mTorr, Cl_2_: Ar = 100/5 sccm) using the SiO_*x*_ mask to complete Si nanograting fabrication.
2. Spacer deposition: The samples were brought into the sputtering chamber (Lesker PVD 75) and covered with a 520 nm SiO_*x*_ spacer layer (250 W) at a rate of 0.6 Å/s.
3. Vertically coupled double-layered Al gratings: After spacer layer deposition, double-layer PMMA (70 nm 2.5% 495k followed with 50 nm 2% 950k) were coated again, post-baked, and exposed by EBL aligned to the first layer. Then the sample was cleaned with Oxygen plasma to remove residual PMMA on the exposed region. Next, 3 nm Cr and 12 nm SiO_*x*_ were deposited and lifted off, as mentioned above, to form a SiO_*x*_ mask for Cr discharge layer etching. Next, Cr discharging layer is etched by RIE to form Cr/ SiO_*x*_ layered nanostructures, which masked 100 nm SiO_*x*_ RIE etching to form SiO_*x*_ nano-gratings. Then 2 nm Cr and 80 nm Aluminum is deposited by E-beam evaporation, forming vertically coupled Aluminum (Al) gratings.
4. U.V. bonding: After sample fabrication is completed and essential device characterization, the sample was then cut into 4 mm * 3 mm by a dicing saw. A CMOS sensor IMX477 was customized to remove the cover glass, micro lens, and Bayer pattern by MaxMax. corp ltd. Then it was spin-coated with 90% UV at a spin speed of 3000 rpm/s; the sample was then visually aligned and bonded onto the CMOS sensor by a homemade transfer setup. The detailed schematic of homemade transfer setup is illustrated in Supplementary Information (Fig. S11). Afterward, U.V. resists were cured by a 365 nm U.V. lamp (100 W) for 20 min of illumination.

### Measurement

#### Device transmission and extinction ratio characterization

For the chiral metasurface, the unpolarized laser was first polarized by linear polarizer (WP25M-UB by Thorlabs, Inc.) and super achromatic QWP (SAQWP05M-700 by Thorlabs, Inc.) to generate LCP, RCP input, respectively. The CP light is then focused onto the sample with a focal spot size of 15 um in diameter. The transmission efficiency was then measured using Olympus BX53 fluorescent microscope and Horiba iHR320 visible spectrometer. The CPER of LCP chiral metasurface was calculated using the formula: $E={T}_{{LCP}}/{T}_{{RCP}}$, where ${T}_{{LCP}},\,{T}_{{RCP}}$ denotes the transmission efficiency of LCP and RCP input, respectively. As for double-layer gratings, the transmission efficiency of TE-mode TM mode linearly polarized input was measured respectively to calculate LPER, using the equation LPER = T_*x*_/T_*y*,_ where T_*x*_, T_*y*_ denotes the transmission efficiency of LP input along x axis and y axis, respectively.

#### Instrument matrix calibration for polarimetric Imager

Eight polarization states were induced with a broadband linear polarizer (WP25M-UB by Thorlabs, Inc.) and super achromatic QWP (SAQWP05M-700 by Thorlabs, Inc.). The induced polarization states are then normally incident onto the polarimetric imager. Images were taken with sufficient exposure time to ensure a high enough signal-to-noise ratio (SNR). The instrument matrix of each super-pixel was then calculated in MATLAB according to the transmitted intensities of each metasurface filter.

#### Full Stokes polarization detection measurement

Light coming from a High-Intensity Fiber coupled Halogen lamp light source (Thorlabs OSL2) is firstly collimated using a parabolic mirror (Thorlabs MPD129-P01), and the iris is applied to control the beam divergence angle. The bandpass filter (red: FBH650-40 green: FBH500-40) is applied for wavelength selection. Lens with F = 30 mm (AC254-030-AB) and F = 100 mm (AC254-100-AB) are applied for beam expansion. The final beam divergence is controlled to be 0.5 degrees with a spot size of 9 mm in diameter. Arbitrary polarization states were generated using a broadband linear polarizer (WP25M-UB by Thorlabs, Inc.) and super achromatic QWP (SAQWP05M-700 by Thorlabs, Inc.) A list of Stokes parameters was first designed, then each polarization state was normally incident onto the polarimetric imager. These polarization states were first measured by the rotation of a linear analyzer (LPIREA100-C); the transmitted intensity was then fitted to obtain the polarization states. Afterward, the polarization states were captured by the polarimetric imager at a normal incidence angle. The images were then transferred onto a computer to calculate polarization states according to the transmitted intensities and the calibrated instrument matrix of each metasurface filter. The code for extracting polarization states for all super-pixels as well as Stokes parameter measurement is performed in a MATLAB environment.

#### Full Stokes polarization imaging

A camera zoom lens is applied for imaging purposes. A color filter is attached in front of the lens. The field of view of the camera lens applied is ~40 degrees for imaging demonstration.

### Supplementary information

Supplementary information
