# Supplementary material for: Chip-integrated metasurface full-Stokes polarimetric imaging sensor
Source: Light Sci Appl. 2023 Sep 6;12:218. doi: 10.1038/s41377-023-01260-w (PMC10482842; doi:10.1038/s41377-023-01260-w)
Supplement: Supplementary file 1 — Supplementary information [file 41377_2023_1260_MOESM1_ESM.docx]

Supplementary Information for:

**"** **Chip-Integrated Metasurface Full-Stokes Polarimetric Imaging Sensor"**

*Jiawei Zuo^1,2^, Jing Bai^1,2^, Shinhyuk Choi^1,2^, Ali Basiri^1,2^, Xiahui Chen^1,2^, Chao Wang^1,2,3^, Yu Yao^1,2^**

^1^School of Electrical, Computer and Energy Engineering, Arizona State University, Tempe, AZ, USA, 85281

^2^Centre for Photonic Innovation, Arizona State University, Tempe, AZ, USA, 85281

^3^Biodesign Center for Molecular Design &Biomimetics

***Corresponding authors：** [**yuyao@asu.edu**](mailto:yuyao@asu.edu)

Contents

[1. Comparison of full Stokes polarimetric imaging sensors 2](#_Toc141304929)

[2. Design of VCDG and chiral metasurface structures 7](#_Toc141304930)

[3. Fabrication and spectroscopic characterization of Chiral metasurface and VCDG 13](#_Toc141304931)

[4. Analysis of degradation of optical performances of Chiral metasurface and VCDG due to fabrication 18](#_Toc141304932)

[5. Instrument matrix calibration process 24](#_Toc141304933)

[6. Full Stokes polarization measurement at different incidence angle 26](#_Toc141304934)

[7. Full Stokes polarization imaging of ASU logo with polarization information 47](#_Toc141304935)

## Comparison of full Stokes polarimetric imaging sensors

**Supplementary Table 1**. State of the Art polarimetric imaging systems based on different types of technologies.

| Full Stokes polarimetric imagers | Chip integrated? | Compactness of polarization filter/micro filter array (thickness) | Detection error | Full Stokes Parameter imaging /detection |
| --- | --- | --- | --- | --- |
| Our Work  (metasurface-based) | Yes | ~600 nm | <2%(S1,S2,S3), DOLP<1.5%, DOCP<2% AOP<0.8° at normal incidence  <4%(S1,S2,S3) DOLP<2.28%  DOCP<4%,  AOP<0.9° at ±20° oblique incidence (red color) | Yes |
| Rotating QWP and linear polarizer | No | cm scale | DOP error <1% [1] | Yes |
| Rotational Liquid Crystal and a fixed linear polarizer | No | mm~cm scale [2] | Elliptical polarization:  DOCP error< 2%  DOLP error<3%[2] | Yes |
| Metallic wire-grid LP filters | Yes | 300 nm [3] | LPER = 80~450[3]  DOLP error < 2% characterized in lab | No  (Cannot measure S3) |
| Birefringent polymer integrated on micro-LP filters array | Yes | 2 μm [4] | full Stokes image of LCD screen:  DOCP error <6.7%  DOLP error < 3.35%  AOP error < 1.90°[4] | Yes |
| Liquid crystal polymer based DoFP polarimeter | Yes | 1 μm[5]  5 μm [6] | DOCP error: < 25%  DOLP error:<18% [5]  LPER =1100 [6]  <2.3%(S1,S2,S3)[6] | Yes |

**Supplementary Table 1**. State of the Art polarimetric imaging systems based on different technologies (continued)

| Polarimetric imagers | PFOV demonstrated. | Operational wavelength | Pixel size | Detection Speed | Single shot? |
| --- | --- | --- | --- | --- | --- |
| Our Work | 40° for Red  10° for Green | 630-670 nm(red)  480-520 nm(green) | 6.2μm | 15 fps to 30 fps | Yes * |
| Rotating QWP and linear polarizer | <2°[1] | 400 to 700 nm[1] | NA | 30 sample/s to 400 sample /s[1] | No |
| Rotational Liquid Crystal and a fixed linear polarizer | NA | 520-550 nm[2] | NA | 15- 20 fps | No |
| Metallic wire-grid LP filters | 45° | 400-900 nm[3] | 3.45 μm | 22 fps | Yes |
| Birefringent polymer integrated on micro-LP filters array | NA | 400-700 nm[4] | 6.9 μm[4] | 22 fps | Yes |
| Liquid crystal polymer based DoFP polarimeter | NA | 577.5-582.5 nm[5]  400-700 nm [6] | 7.4 μm[5]  20 μm[6] | <1 fps  4.5S per frame[5] | Yes |

(* Full Stokes polarization images can be obtained with a single snapshot)

**Supplementary Table 2**. Full Stokes polarimetric imagers based on metasurface structures.

| Full Stokes polarimeters | materials | Chip integrated? | Detection error | PFOV | Bandwidth | Pixel Number of polarization images |
| --- | --- | --- | --- | --- | --- | --- |
| Our work | Dielectric-metal hybrid | Yes | <2% (S1, S2, S3) | Up to 40° | Dual color  630-670 nm(red)  480-520 nm(green) | 335×224 |
| Dielectric metasurface diffraction grating | Dielectric | No  (Requires spacing to diffract incoming light) | Polarization contrast error < 10%[7]  Error: 10%~20%[8] | 10°[7]  normal incidence[8] | Narrow band  527 to 537nm[7]  Narrow band  530 nm [8] | 3M[7]  13×13 [8] |
| Microscale polarization metalens array |  | No  (requires spacing for focus) | averaged relative error.  <4.85% (S1, S2, S3)[9] | normal incidence | Narrow band  1550 nm[9] | 5×10 [9] |
| Microscale polarization metalens splitter array |  | No  (requires spacing for focus) | 7.5-15% (S1,S2,S3) [10] | normal incidence | Narrow band  845-855 nm [10] | 70×46 [10] |
| Plasmonic metasurface microscale polarization filter array | Plasmonic | No  (requires relay lens) | <6%(S1),  <8% (S2),  < 7%(S3)[11] | normal incidence | Broadband  4~9 μm [11] | 30×25 |

**Supplementary Table 3**. Full Stokes single point polarimetric detectors based on metasurfaces

| Full Stokes polarimeters | materials | Chip Integrated? | Detection error | PFOV | Operational wavelength | Pixel number |
| --- | --- | --- | --- | --- | --- | --- |
| Our Work | Dieletric metal Hybrid | Yes | <2% (S1,S2,S3) at normal incidence  <4% (S1,S2,S3) at ±20° incidence at red color | 40° for red  10° for cyan | 630-670 nm(red)  480-520 nm(green) | 168×56 super pixels |
| Graphene, plasmonic hybrid | metal | No | <3.9% (S1),  <6.5% (S2),  <2.5% (S3)[12] | normal incidence | 6.7 μm-6.8 μm[12] | NA |
|  |  | Yes | <50%(S3)  Ellipticity:7~10degree  AOP:7~14 degree[13] | normal incidence | 1.55 μm[13] | NA |
| Plasmonic metasurface |  | Yes | <0.44%(S1),  <1.4% (S2),  < 0.79%(S3)[14] | normal incidence | 3.5 to 5 μm[14] | NA |
|  |  | No | 7.3–12.3% (S1),  7.2–27.4% (S2),  5.2–17.7% (S3)[15] | normal incidence | 830 nm[15] | NA |
|  |  | Yes | <2.3% (DOLP)  <10.3% (DOCP)[16] | normal incidence | 3.725 μm to 3.875 μm[16] | NA |
|  |  | No | <45% (S1, S2, S3) [17] | normal incidence | 750-1050 nm[17] | NA |
| Plasmonic absorber |  | No | NA | Normal incidence | 5 to 6 μm[18] | NA |

**Supplementary Table 3**. Full Stokes polarimetric detectors based on metasurfaces(continued)

| Full Stokes polarimeters | materials | Chip integrated? | Detection error | PFOV | Operational wavelength | Pixel Number |
| --- | --- | --- | --- | --- | --- | --- |
| Our work | Dielectric-metal hybrid | Yes | <2% (S1,S2,S3) at normal incidence  <4% (S1,S2,S3) at ±20° incidence at red color | 40° for red  10° for cyan | 630-670 nm(red)  480-520 nm(cyan) | 168×56 super pixels |
| Dielectric Nanobar on gold nanowires |  | Yes | <1.9% (S1),  <2.7% (S2),  <7.2% (S3)[19] | normal incidence | 1.3-1.6 μm | NA |
| Dielectric diffraction grating | dielectric | No | S1 S2 S3 averaged fidelity:  99.27 ± 0.86%[20] | Normal incidence | Narrow band at 808 nm | NA |

## Design of VCDG and chiral metasurface structures


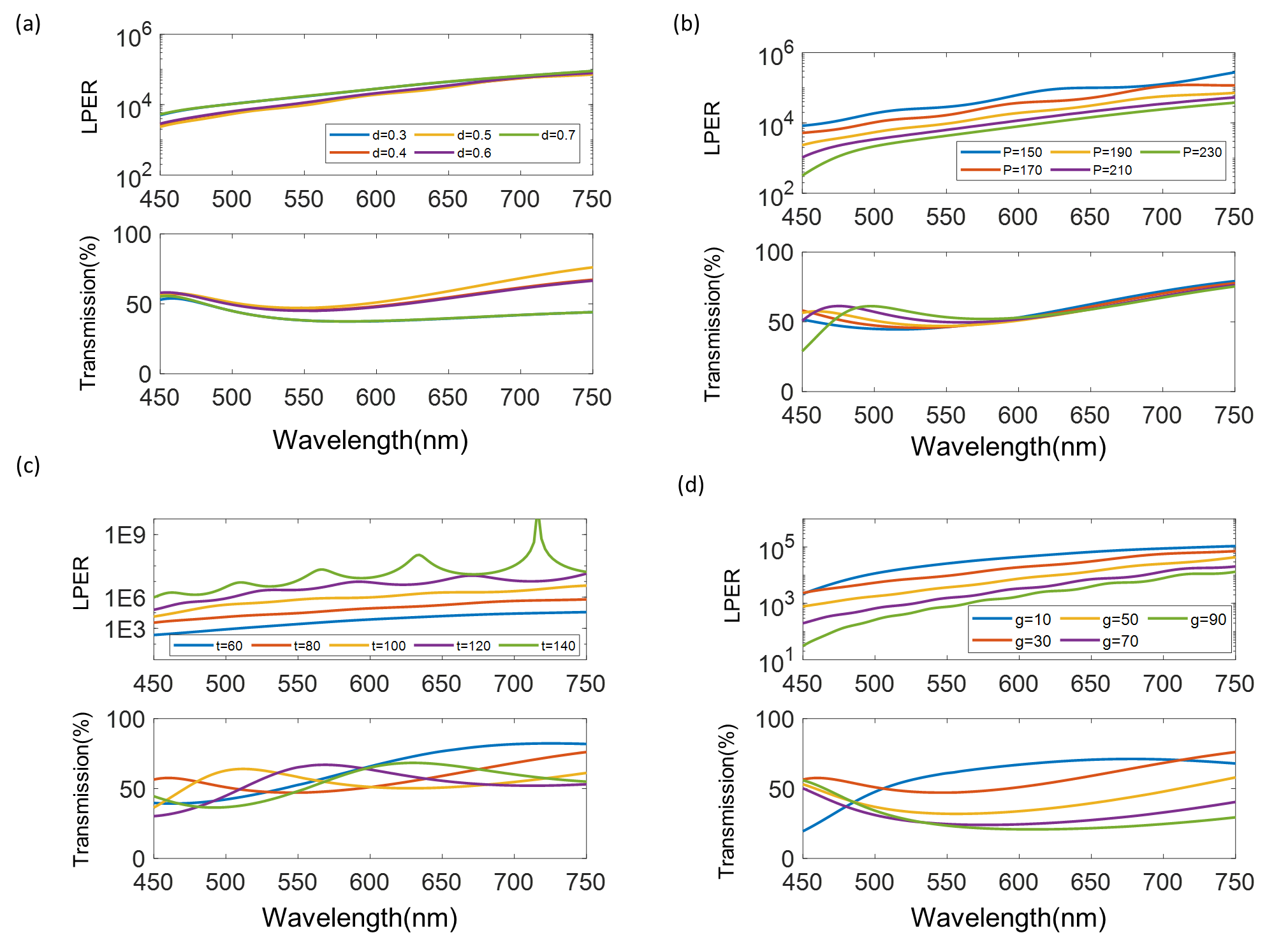


**Supplementary Figure 1. Parameter scan of VCDG’s duty cycle d, period P, Aluminum thickness t and vertical gap g.** (a) Simulated transmission and LPER as a function of duty cycle written as d, here d is defined as ratio between the width of elevated Al grating(w_Al_) and VCDG period(*p_1_*), other parameters as the same as shown in Fig.2a. (b) Simulated transmission and LPER as a function of VCDG period(p_1_), other parameters as the same as shown in Fig.2a. (c) Simulated transmission and LPER as a function of Aluminum thickness t_Al_, other parameters as the same as shown in Fig.2a. (d) Simulated transmission and LPER as a function of vertical gap written as g. period, other parameters as the same as shown in Fig.2a.


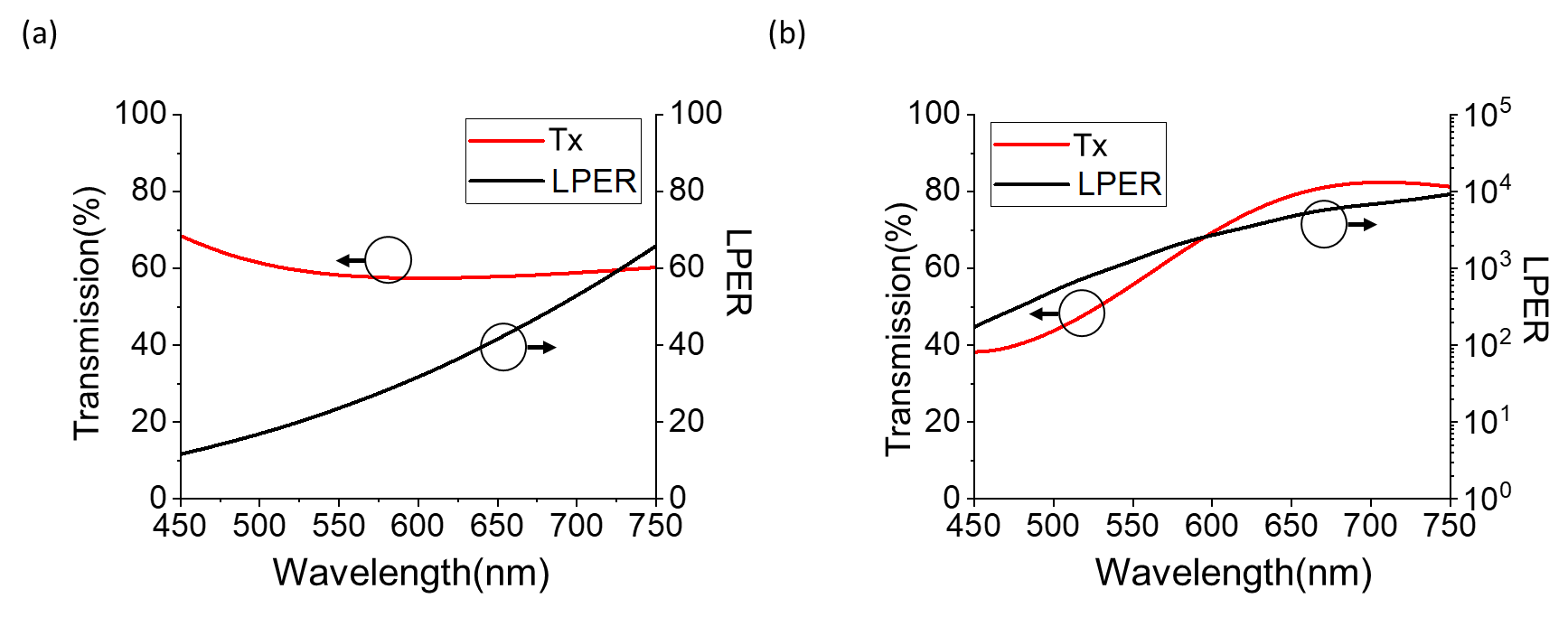


**Supplementary Figure 2. LPER and efficiency of single layered gratings with different thickness**. (a) Transmission efficiency and LPER of single layered grating with Al deposition thickness of 80 nm, which is the same as VCDG design. The period, duty cycle of Al grating is 190 nm and 0.5 respectively. (b) Transmission efficiency and LPER of single layered grating with Al thickness of 180nm, the period, duty cycle of Al gratings is 190 nm and 0.5 respectively.

Supplementary Figure 2 shows the LPER and efficiency of single layered gratings with different Al deposition thickness. At same thickness(80nm), VCDG has 3 orders higher LPER than single layered gratings, we contribute such high LPER of VCDG to its bi-layer nature, which allows transmitted electric field from top Al nanowire to be further damped out by the lower grating. Further, we increased single grating thickness to 180 nm to catch up the LPER difference (Supplementary Figure 1b). However, such thick Al requires Al etching in fabrication, which is a challenging task because both etching uniformity and etching reproducibility needs to be optimized for millimeter-scale fabrication. On the contrary, the fabrication of VCDG does not require the etching of Al but Al deposition, which is much simpler and robust.

*
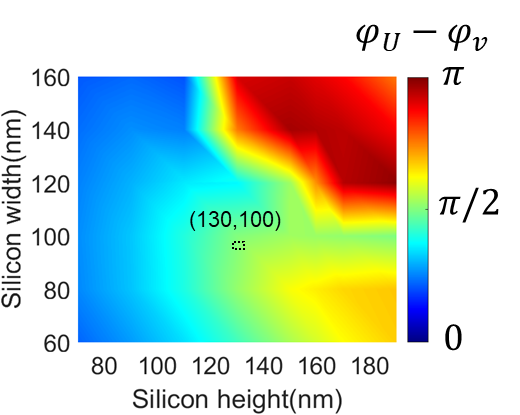
*

**Supplementary Figure 3. Tuning phase difference between U, V axes at 630nm by changing Si grating height and grating width. Other parameters of Si gratings are the same as shown in Fig.2d.**

***
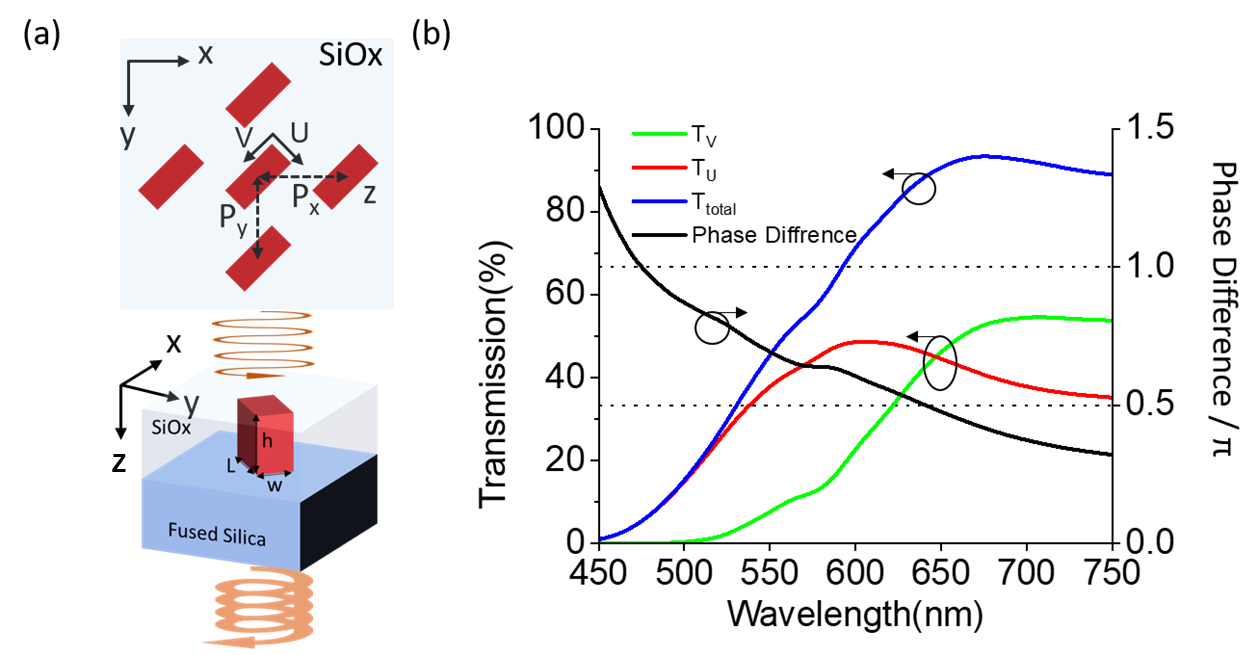
***

**Supplementary Figure 4. Optimized Si Bar nanopillars working as QWP at 645nm**. (a) 2D illustration of Si nanobar array and 3D illustration of Si nanobarn unit cell. Si nanopillar is immersed in SiOx for on chip integration. The width w, length L and height h and period along x axis and y axis of Si nanopillar are *w*=60 nm, *L*=250 nm, *h*=260 nm, *Px=Py*=190 nm respectively. (b) Phase difference between fast axis (U axis) and slow axis (V axis), left axis, and correspondent transmission along U axis(red) and V axis(green) and total transmission (black) (right axis).


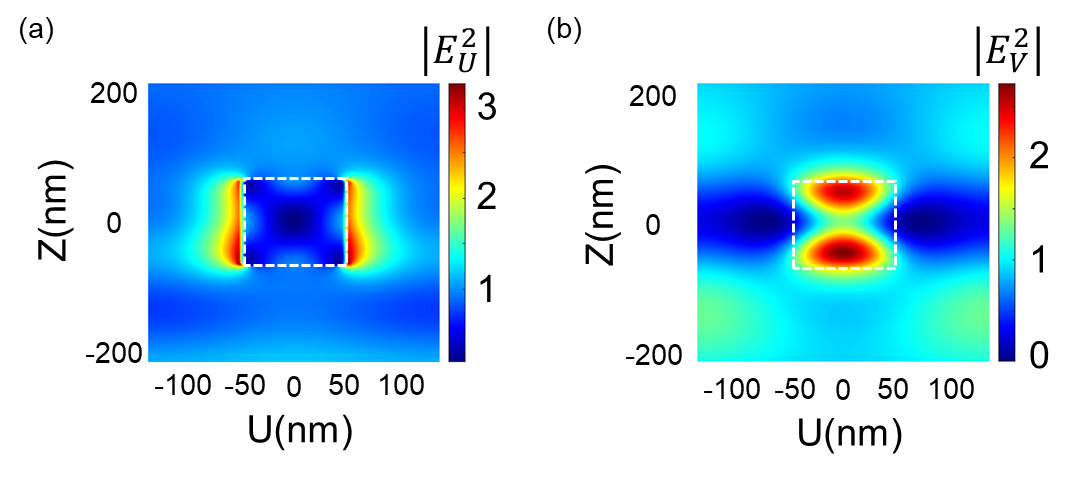


**Supplementary Figure 5. Cross-sectional near field distribution of Si gratings for input LP along grating U,V axes, respectively.** **Si grating tilt angle θ_Si_ were considered to be 0° , other parameters of Si gratings were the same as shown in Fig.2d.**


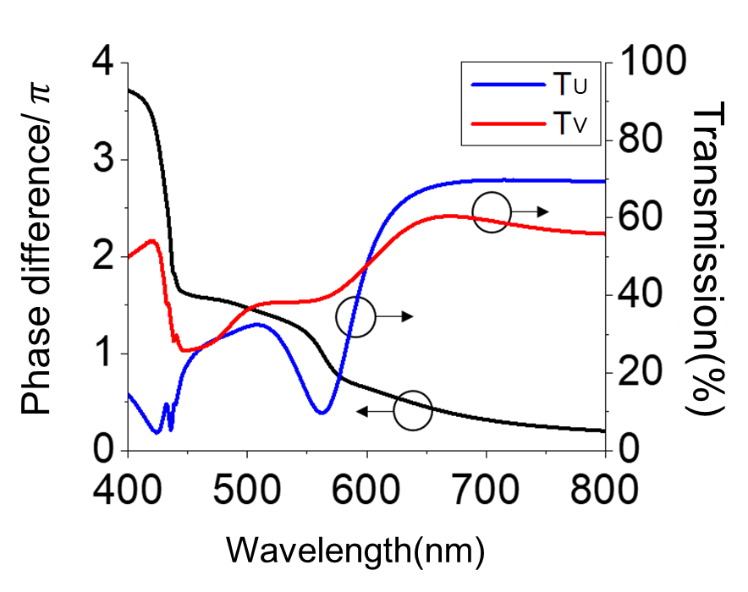


**Supplementary Figure 6. Phase difference between E_U_ and E_V_ calculated by FDTD simulation. Si grating tilt angle *θ_Si_* were considered to be 0°, other parameters of Si gratings were the same as shown in Fig.2d.**

***
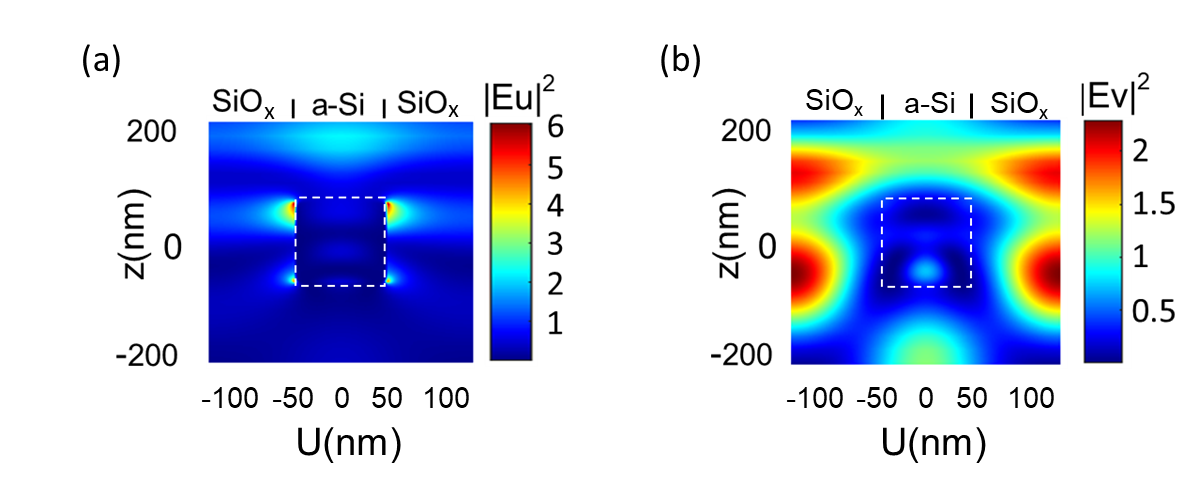
***

**Supplementary Figure 7. Cross-sectional near field distribution of Si grating at 500nm. Si grating tilt angle *θ_Si_* were considered to be 0°, other parameters of Si gratings were the same as shown in Fig.2d.**


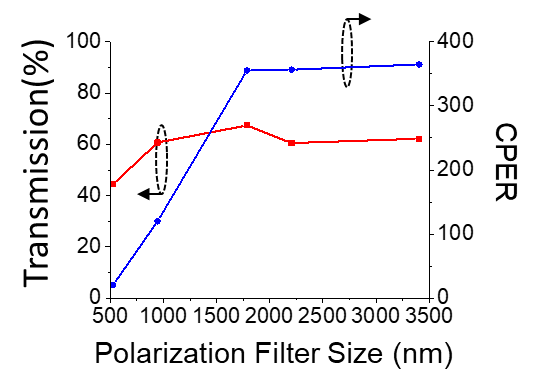


**Supplementary Figure 8.** **Simulated transmission and CPER dependence on lateral dimensions of CP filter at red color.**

Figure S8 shows simulated CPER and transmission dependence on finite CP filter size. PML boundary conditions for all directions were applied, square-shaped Al metal frames with 1 μm width and 80 nm thickness were used to enclose the chiral metasurface and eliminate the diffraction from PML boundary. As shown above, CPER is above 100 and transmission is more than 60 % with a pixel size of $0.945\times0.945 \mu m^{2}$.

***
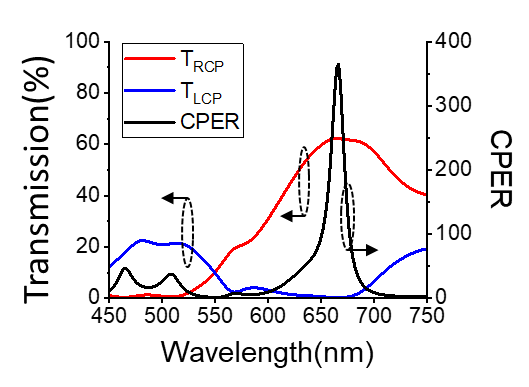
***

**Supplementary Figure 9.** Simulated transmission and CPER at CP filters’ lateral dimensions of 3.045 μm×3.045 μm. The dimensions of Si nanogratings and VCDGs and spacer layer thickness are the same as Fig. 2d.

## Fabrication and spectroscopic characterization of Chiral metasurface and VCDG


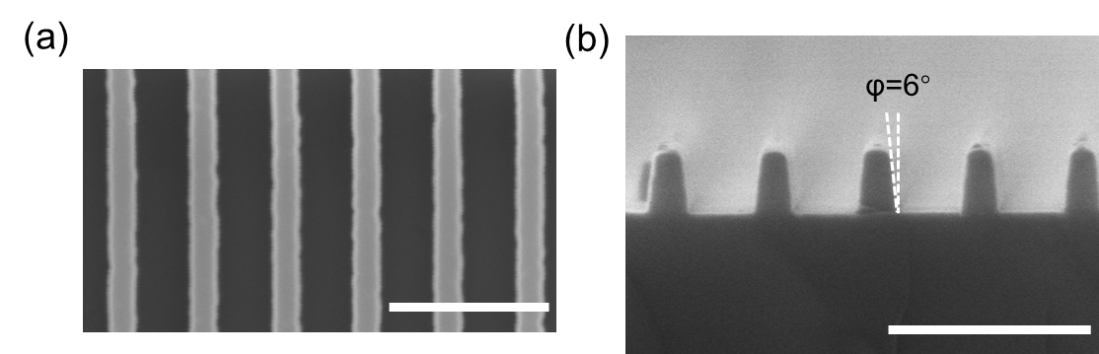


**Supplementary Figure 10. Top view and Cross-section SEM image of the dry etched Si gratings. (a) Top view SEM, scale bar: 500nm. (b) Cross-section SEM, scale bar: 500nm. Tilt angle of Si grating is 6° due to partially anisotropic Si dry etching.**

Figure S10 shows the dimensions of Si gratings. The thickness (*t_si_*), period (*p_Si_*), width (*w_Si_*), and tilted angle (*θ_Si_*) of fabricated Si nanograting are *t_si_*=130 nm, *p_Si_*=297 nm, *w_Si_*=108 nm and *θ_Si_*=6° respectively.

Supplementary Table 4 Summary table of dimensions of fabricated VCDGs and chiral metasurface

| dimension name | VCDG period: *p1* | Al thickness *t_Al_* | Al linewidth w_Al_  (Duty cycle %) | VCDG Vertical gap: *g* | Si width: *w_Si_* | Si grating period: *p_si_* | Si grating thickness:  *t_si_* |
| --- | --- | --- | --- | --- | --- | --- | --- |
| 0° LP filter (P1) | 190 nm | 80 nm | 105 nm (55%) | 30 nm | NA | NA | NA |
| 45° LP filter (P3) | 190 nm | 80 nm | 87 nm (46%) | 30 nm | NA | NA | NA |
| 90° LP filter (P2) | 190 nm | 80 nm | 89 nm (47%) | 30 nm | NA | NA | NA |
| 135° LP filter (P4) | 190 nm | 80 nm | 88 nm (46%) | 30 nm | NA | NA | NA |
| LCP CP filter (P5, P5’) | 210 nm | 80 nm | 97 nm (46%) | 30 nm | 108 nm | 297 nm | 130 nm |
| RCP CP filter (P6, P6’) | 210 nm | 80 nm | 98 nm (47%) | 30 nm | 108 nm | 297 nm | 130 nm |


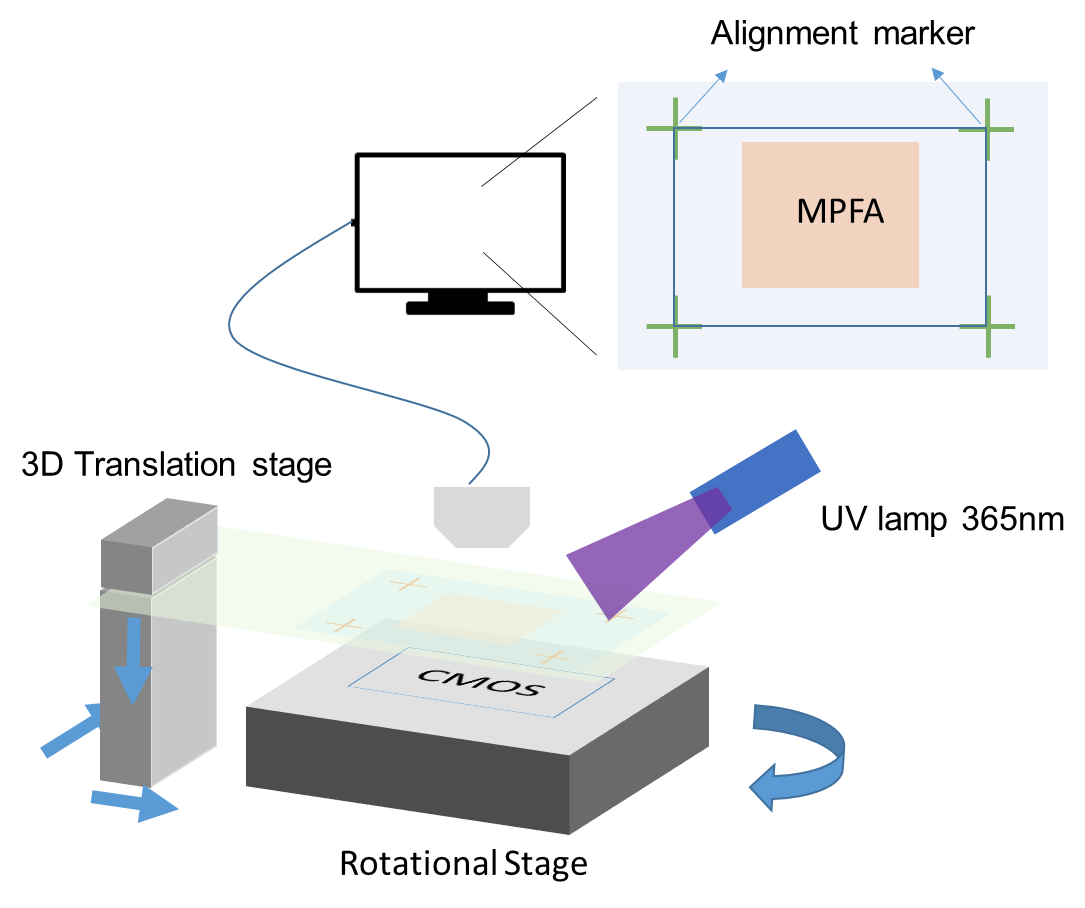


**Supplementary Figure 11. Homemade CMOS bonding setup.** The device is taped onto a glass slide and then mounted on a 3D translation stage. Meanwhile, the CMOS imaging sensor is spin coated with UV resist and mounted onto the rotational stage. Then the device is visually aligned to the CMOS imaging sensor using pre-designed alignment marker. After alignment, the device is bonded on the CMOS imaging sensor via UV bonding.

*
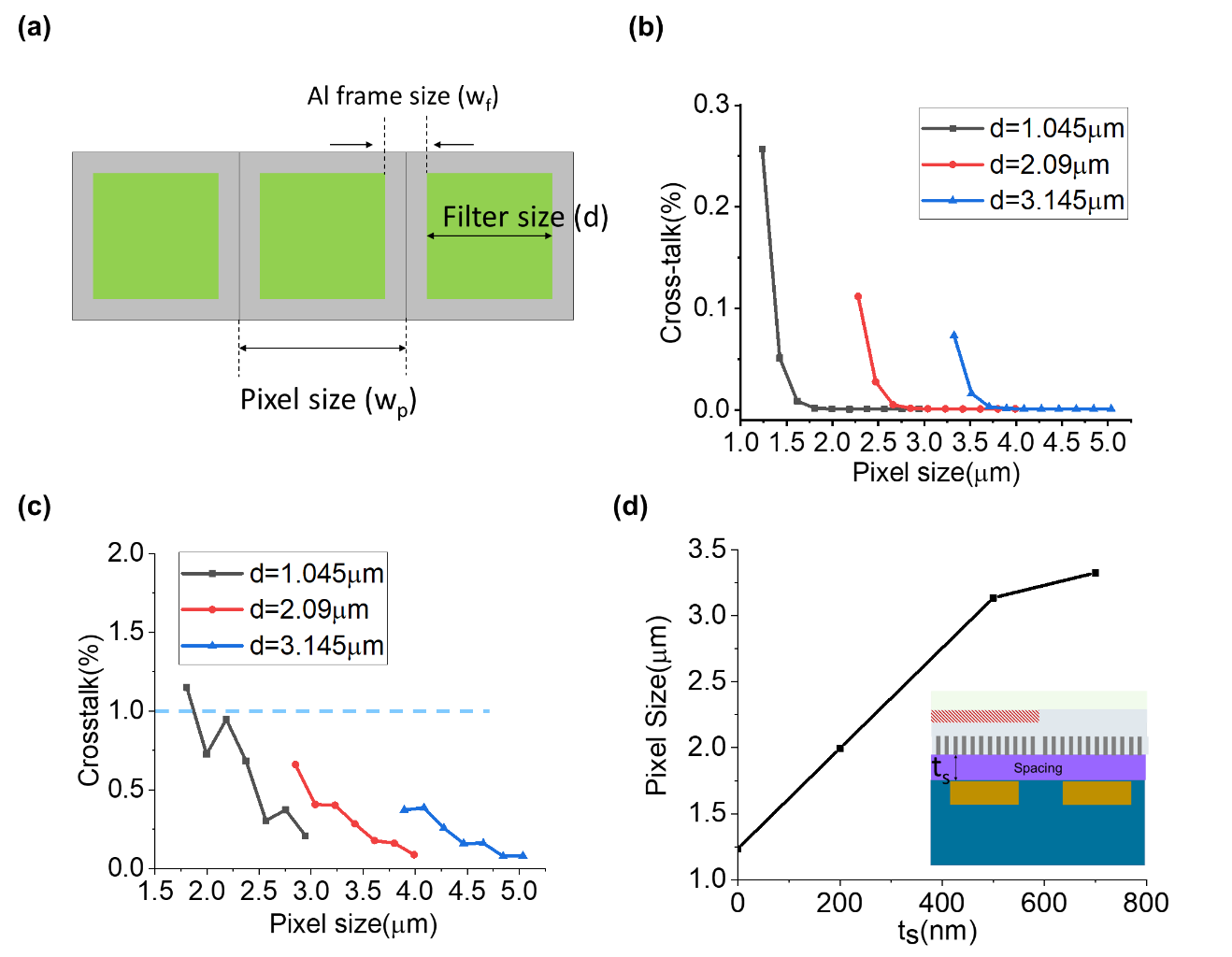
*

**Supplementary Figure 12.** **The dependence of crosstalk ratio on polarization filters dimensions and Al frame size. All the polarization filter dimensions used in simulation are the same as shown in Fig. 2a and Fig.2d.** (a) Schematic to show the configuration of a subpixel. (b) Dependence of crosstalk ratio on pixel size when polarization filter is fabricated on photodetector directly (*t_s_*=0 nm). (c) Dependence of crosstalk ratio on pixel size when spacing (*t_s_*) between photodetector and polarization filter is 200 nm (*t_s_*=200 nm). (d) Dependence of pixel size on the distance (*t_s_*) from polarization filter to the photo detector to ensure crosstalk is less than 1%.

Figure S12 shows the dependence of crosstalk ratio on polarization filter size and Al frame size. Gaussian beam was used as the light source and PML boundary conditions for all directions were applied, Al metal frames with 80nm thickness were used. Material with refractive index of *n*=3.5, *k*=0.3 is considered to represent Si photodiode. The adjacent pixels were blocked by the Al thin films, here the crosstalk is defined as the intensity $I_{a}$ recorded by adjacent pixels divided by the transmitted intensity $I_{0}$ at polarization filter region: $cross-talk =I_{a}/I_{0}$. When the polarization filter is fabricated directly on the Si photodiode (*ts*=0, see Figure.S12d). 1μm pixel size can ensure optical crosstalk less than 1% (Figure.S12b). Minimum required pixel size increases with the gap (*ts*) between the polarization filter and photodiode to increased optical diffraction (Figure.S12c). For example, a minimum of 3.3 μm (Al frame size included) is required to ensure crosstalk less than 1% for *ts* = 700 nm. (Figure.S12d).

*
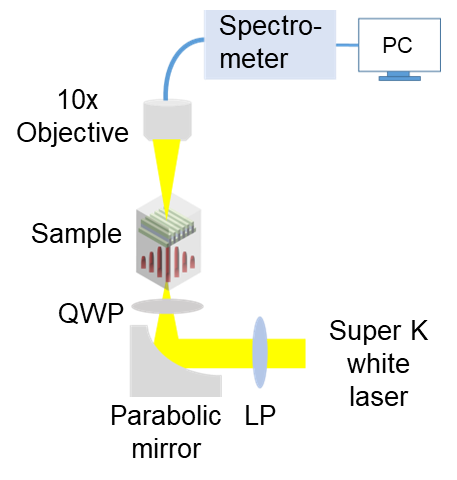
*

**Supplementary Figure 13. Schematics of the spectroscopy setup for chiral metasurface and VCDG characterization.**

## Analysis of degradation of optical performances of Chiral metasurface and VCDG due to fabrication


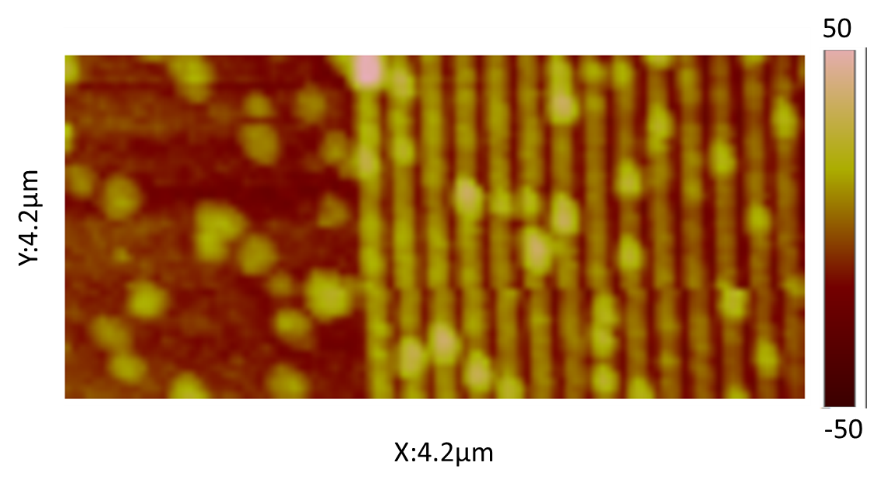


**Supplementary Figure 14. AFM image of VCDG before Al deposition. Surface roughness *Ra*=8.43nm at blank regions.**

**
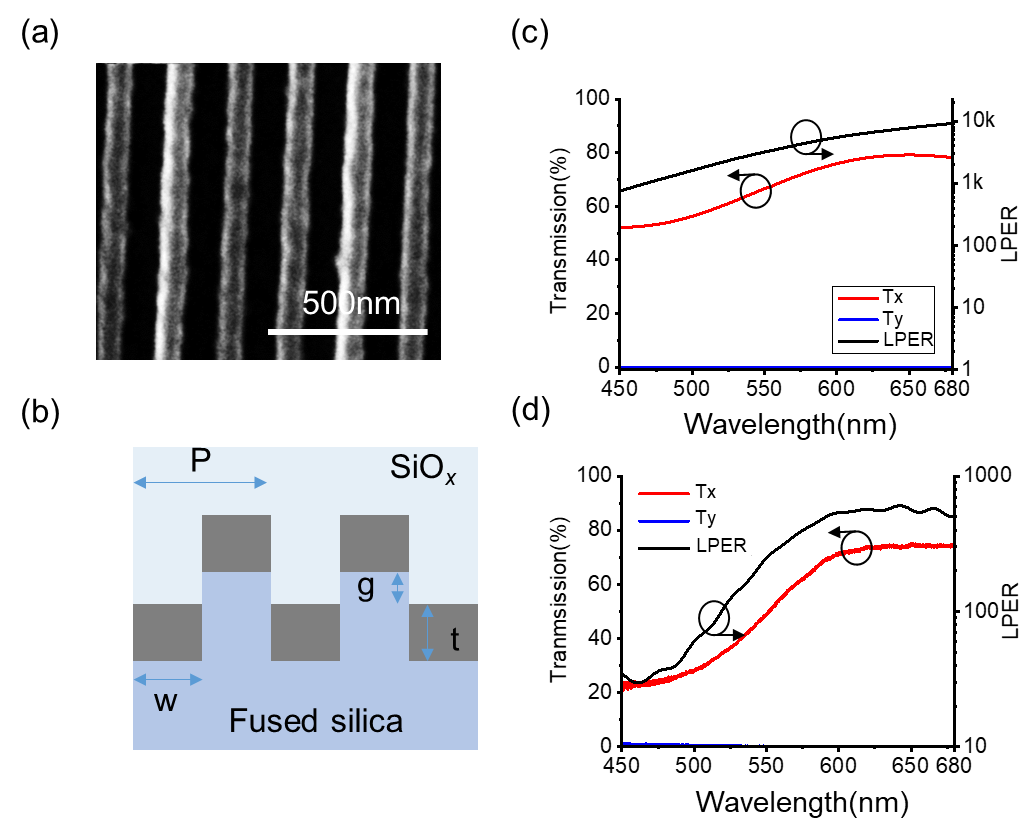
**

**Supplementary Figure 15. SEM image and characterization of VCDG fabricated by EBL patterning on fused silica wafer.** (a) SEM image of VCDG, after taking SEM images, 200 nm *SiOx* is sputtered as encapsulation layer on device. (b)2D cross sectional illustration of VCDG fabricated, the period, thickness of Al, vertical gap size and grating width are *P*=180 nm, *t*=60 nm, *g*=20 nm and *w*=88 nm respectively. (c) Simulated transmission and LPER of VCDG. (d) Measured transmission and LPER of VCDG.


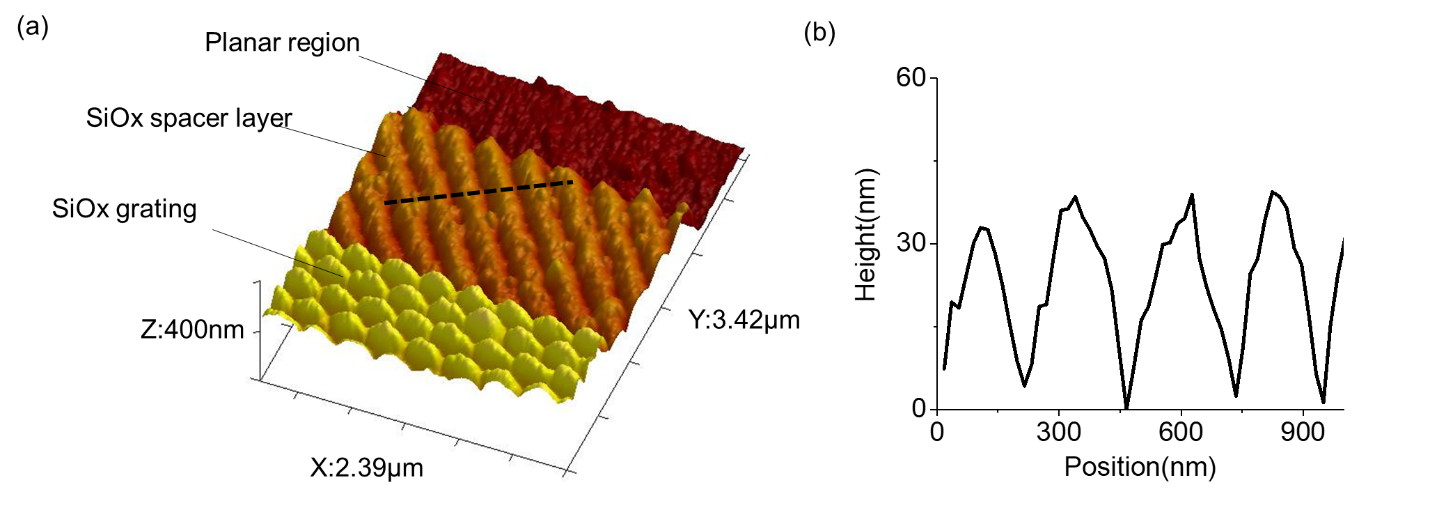


**Supplementary Figure 16. Fabrication of chiral metasurface.** (a). Atomic Force Microscope (AFM) 3D image of SiOx spacer layer and SiOx grating after 2^nd^ EBL patterning followed by dry etching of 100 nm SiOx to form SiOx grating. (b) Height variation of SiOx spacer layer after SiOx etching, indicating surface roughness of SiOx spacer layer (*Ra*= 27.8 nm).

**
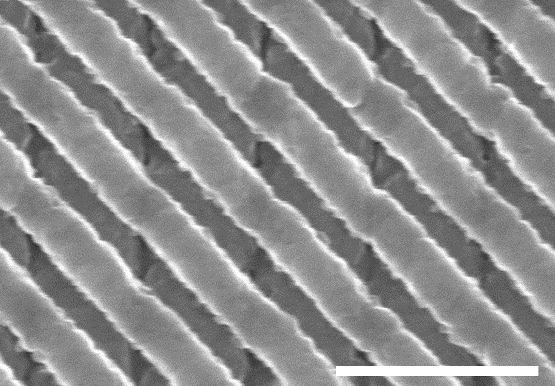
**

**Supplementary Figure 17. SEM of another chiral metasurface (Device B) fabricated by same fabrication procedures, scale bar: 500nm.** The thickness, period, width, and tilted angle of Si nanograting are *t_si_*=130 nm, *p_1_*=268 nm, *w*=100 nm. The thickness of Aluminum (Al), period, and vertical gap of bottom layer VCDG are *t_Al_*= 60 nm, *p_2_*= 190 nm, and *g*=20 nm, respectively. The thickness of the SiO_x_ spacer layer is *t*=335nm measured by Atomic Force Microscope (AFM).

**
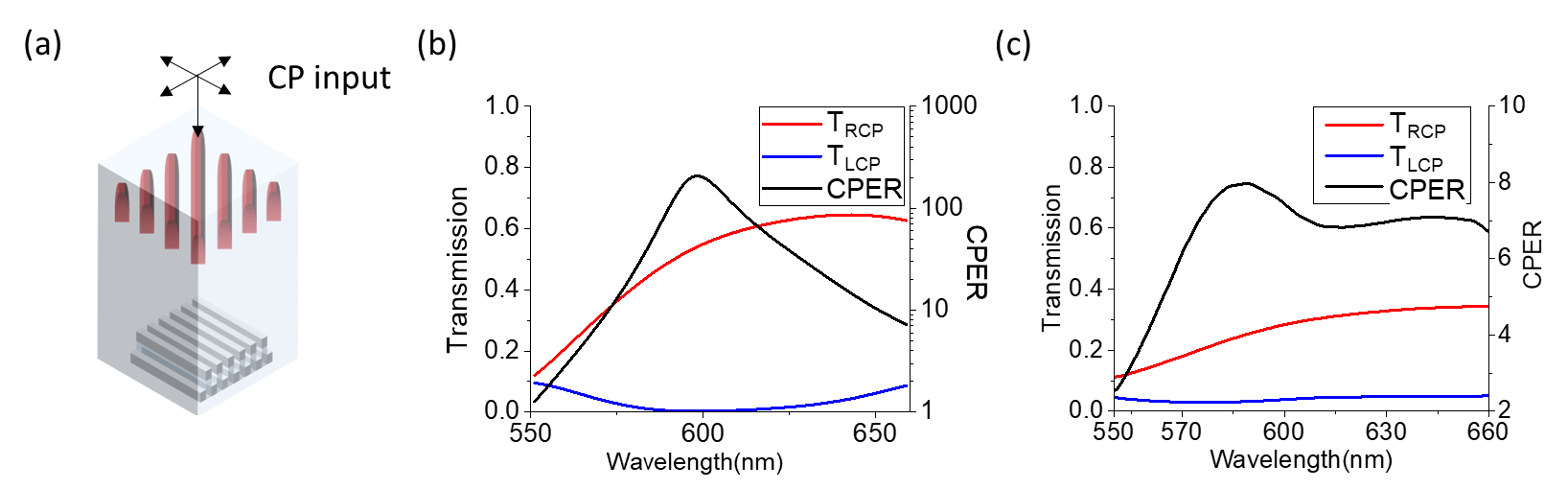
**

**Supplementary Figure 18. FDTD simulated and measured transmission/ CPER of chiral metasurface (device B) with CP light input from Si grating side of chiral metasurface.** (a). 3D schematic to show CP input from the Si grating side of the chiral metasurface. (b). FDTD simulated CPER and efficiency of chiral metasurface. (c). Measured CPER and efficiency of chiral metasurface (device B). The simulated chiral metasurface dimension is the same as dimensions shown in Figure S17.

**
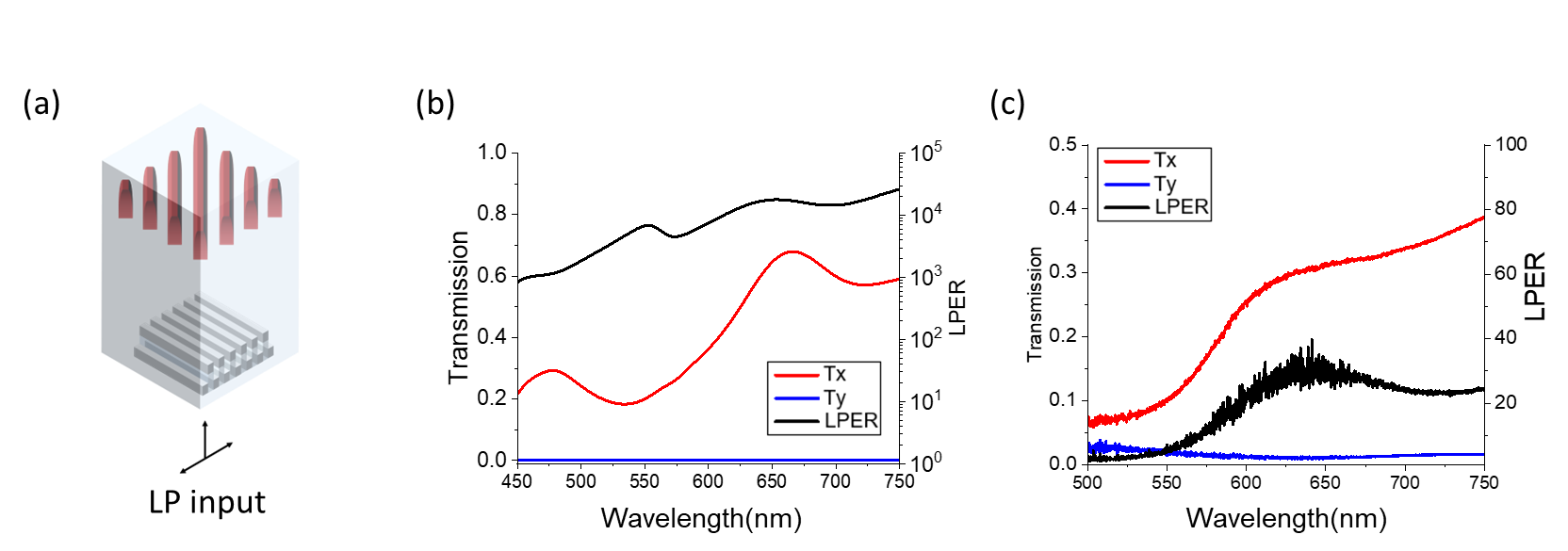
**

**Supplementary Figure 19. FDTD simulated and measured transmission/ LPER of VCDG with LP light incident from VCDG side of chiral metasurface (device B). The thickness of Aluminum (Al), period, and vertical gap of bottom layer VCDG used in simulation are *t_Al_*= 60 nm, *p_2_*= 190 nm, and *g*=20 nm, respectively.** (a). 3D schematic to show LP input from the VCDG side of the chiral metasurface. (b). FDTD simulated LPER and efficiency of chiral metasurface with LP input from VCDG side of chiral metasurface. (c). Measured LPER and efficiency of chiral metasurface with LP input from VCDG side of chiral metasurface.

***
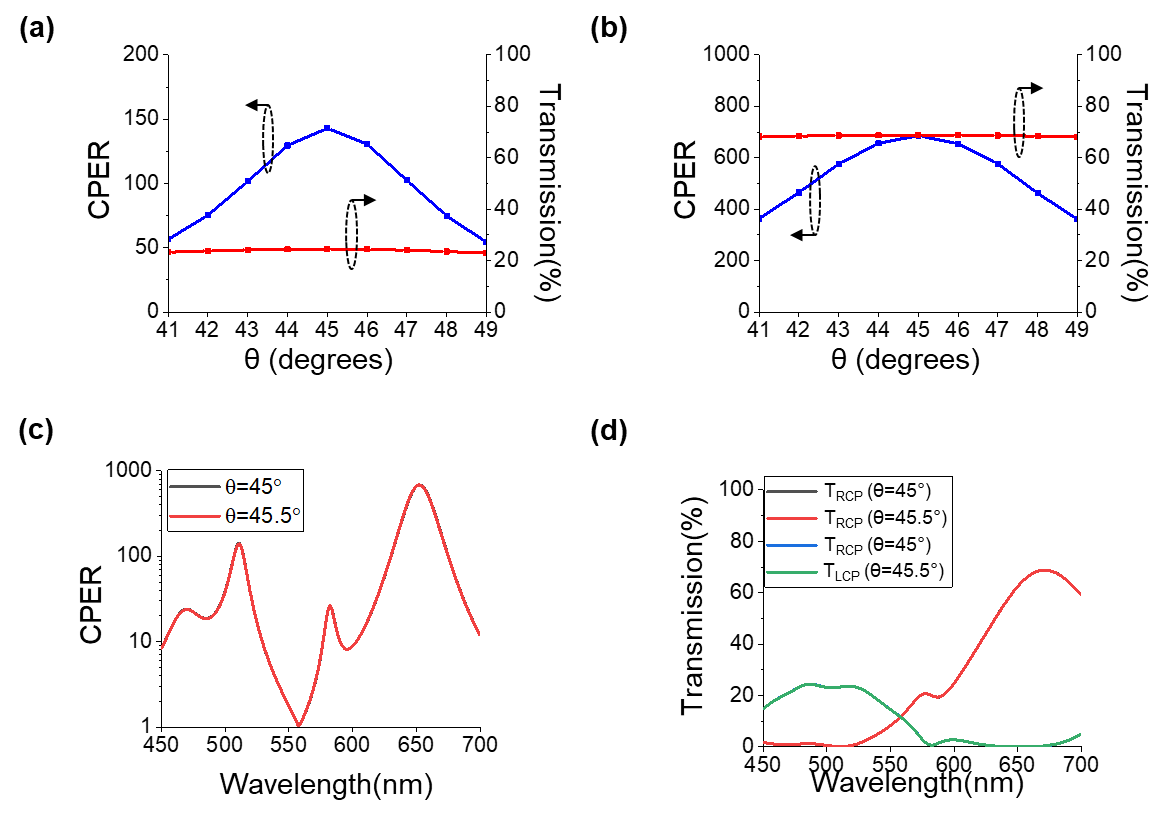
***

**Supplementary Figure 20. Simulated CPER and efficiency dependence of chiral metasurface on orientation angle between top Si grating and bottom VCDGs. The dimensions of Si gratings, spacer layer thickness and VCDGs are the same as presented in Fig.2d.** (a). CPER and efficiency dependency on green color. (b). CPER and efficiency dependency on red color. (c) Simulated CPER spectra of *θ*=45.5° and *θ*=45° respectively. (d) Simulated transmission spectra for *θ*=45.5° and *θ*=45° respectively.


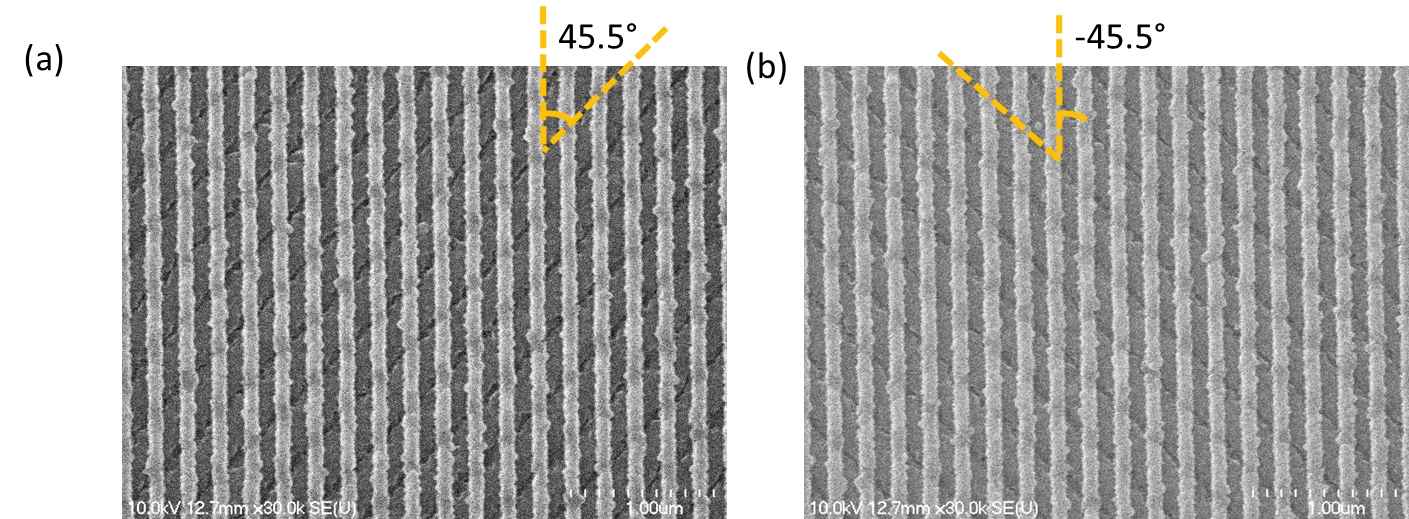


**Supplementary Figure 21.** **Alignment of Si nanogratings and VCDGs.** (a,b) The relative rotation angle of Si nanogratings and VCDGs after alignment is measured from SEM images, indicating ~±0.5° alignment error for RCP and LCP filter respectively.

***
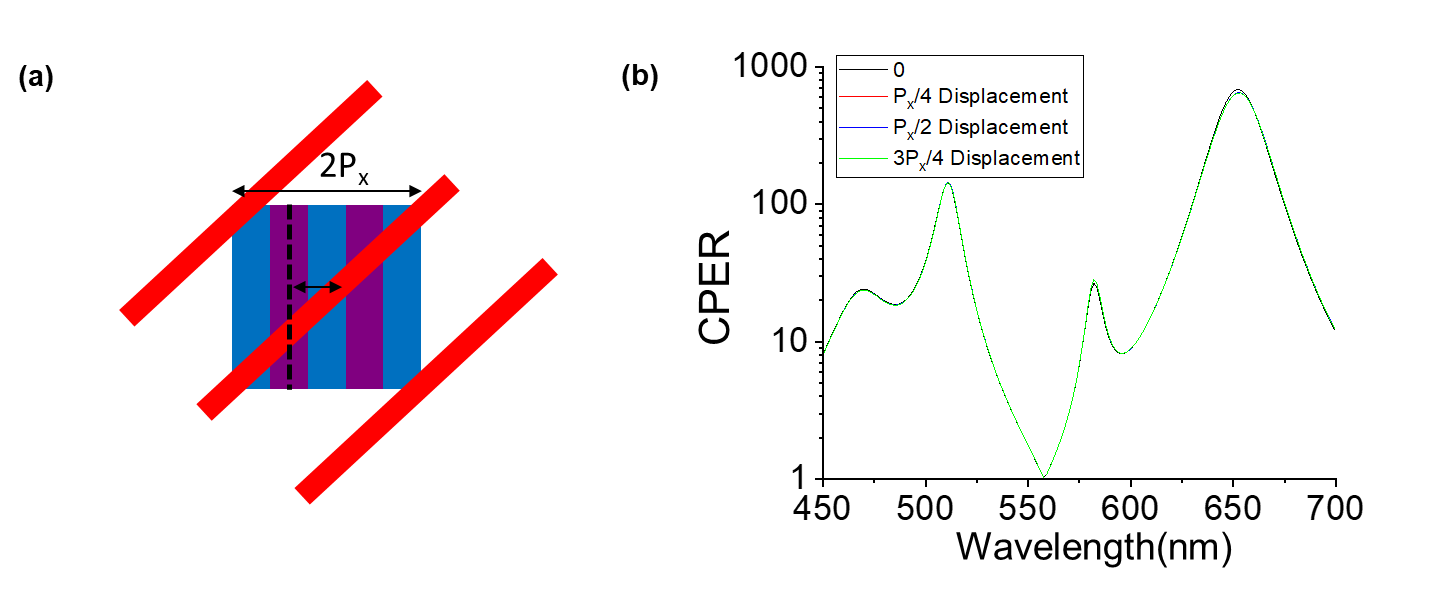
***

**Supplementary Figure 22. Simulated CPER and efficiency dependence of Si grating displacement from bottom VCDGs. The dimensions of Si gratings, spacer layer thickness and VCDGs are the same as presented in Fig.2d.** (a) Illustration to show the alignment of Si grating and bottom VCDGs. (b) CPER of different displacement of Si gratings. Simulation results indicate lateral displacement of Si grating with respect to VCDGs has a slight effect on the CPER by less than 5.4%.

## Instrument matrix calibration process

The transmitted intensity of a linear grating which transmits LP light oriented in a horizontal direction, can be described by the input polarization state $\vec{s}$ And the first row of its Mueller matrix:

|  | $s_{0}^{HG\_out}$=$\left[ \begin{matrix} \begin{matrix} m_{11}^{HG} & m_{12}^{HG} \end{matrix} & \begin{matrix} m_{13}^{HG} & m_{14}^{HG} \end{matrix} \end{matrix} \right]$×$\left[ \begin{matrix} \begin{matrix} s_{0}^{in} \\ s_{1}^{in} \end{matrix} \\ \begin{matrix} s_{2}^{in} \\ s_{3}^{in} \end{matrix} \end{matrix} \right]$ | (1) |
| --- | --- | --- |

Likewise, the output intensity of linear gratings responsible for detection of 90°, 45°, 135° LP light, and chrial metasurface responsible for detection of LCP and RCP light can also be described by the input polarization state and their first row of mueller matrix:

|  | $I=\left[ \begin{matrix} \begin{matrix} s_{0}^{0G\_out} \\ s_{0}^{90G\_out} \end{matrix} \\ \begin{matrix} s_{0}^{135G\_out} \\ s_{0}^{45G\_out} \end{matrix} \\ \begin{matrix} s_{0}^{LCPG\_out} \\ s_{0}^{RCPG\_out} \end{matrix} \end{matrix} \right]=\left[ \begin{matrix} \begin{matrix} \begin{matrix} \begin{matrix} m_{11}^{0G} & m_{12}^{0G} \end{matrix} & \begin{matrix} m_{13}^{0G} & m_{14}^{0G} \end{matrix} \end{matrix} \\ \begin{matrix} \begin{matrix} m_{11}^{90G} & m_{12}^{90G} \end{matrix} & \begin{matrix} m_{13}^{90G} & m_{14}^{90G} \end{matrix} \end{matrix} \end{matrix} \\ \begin{matrix} \begin{matrix} \begin{matrix} m_{11}^{135G} & m_{12}^{135G} \end{matrix} & \begin{matrix} m_{13}^{135G} & m_{14}^{135G} \end{matrix} \end{matrix} \\ \begin{matrix} \begin{matrix} m_{11}^{45G} & m_{12}^{45G} \end{matrix} & \begin{matrix} m_{13}^{45G} & m_{14}^{45G} \end{matrix} \end{matrix} \end{matrix} \\ \begin{matrix} \begin{matrix} \begin{matrix} m_{11}^{LCPG} & m_{12}^{LCPG} \end{matrix} & \begin{matrix} m_{13}^{LCPG} & m_{14}^{LCPG} \end{matrix} \end{matrix} \\ \begin{matrix} \begin{matrix} m_{11}^{RCPG} & m_{12}^{RCPG} \end{matrix} & \begin{matrix} m_{13}^{RCPG} & m_{14}^{RCPG} \end{matrix} \end{matrix} \end{matrix} \end{matrix} \right]\times\left[ \begin{matrix} \begin{matrix} s_{0}^{in} \\ s_{1}^{in} \end{matrix} \\ \begin{matrix} s_{2}^{in} \\ s_{3}^{in} \end{matrix} \end{matrix} \right]=A\times S$ | (2) |
| --- | --- | --- |

The matrix A here is denoted as the instrument matrix of the metasurface filter array. For any unknown polarization state $\vec{s}^{'}$ , it can be inversely calculated via the transmission intensity and the instrument matrix:

|  | $I\vec{s}^{'}=A^{-1}\times I$ | (3) |
| --- | --- | --- |

The measurement of the instrument matrix A can be done by inputting more than four pre-known polarization states measured by traditional angle-resolved measurements:

|  | $A=\left[ \begin{matrix} \begin{matrix} \begin{matrix} \begin{matrix} m_{11}^{0G} & m_{12}^{0G} \end{matrix} & \begin{matrix} m_{13}^{0G} & m_{14}^{0G} \end{matrix} \end{matrix} \\ \begin{matrix} \begin{matrix} m_{11}^{90G} & m_{12}^{90G} \end{matrix} & \begin{matrix} m_{13}^{90G} & m_{14}^{90G} \end{matrix} \end{matrix} \end{matrix} \\ \begin{matrix} \begin{matrix} \begin{matrix} m_{11}^{135G} & m_{12}^{135G} \end{matrix} & \begin{matrix} m_{13}^{135G} & m_{14}^{135G} \end{matrix} \end{matrix} \\ \begin{matrix} \begin{matrix} m_{11}^{45G} & m_{12}^{45G} \end{matrix} & \begin{matrix} m_{13}^{45G} & m_{14}^{45G} \end{matrix} \end{matrix} \end{matrix} \\ \begin{matrix} \begin{matrix} \begin{matrix} m_{11}^{LCPG} & m_{12}^{LCPG} \end{matrix} & \begin{matrix} m_{13}^{LCPG} & m_{14}^{LCPG} \end{matrix} \end{matrix} \\ \begin{matrix} \begin{matrix} m_{11}^{RCPG} & m_{12}^{RCPG} \end{matrix} & \begin{matrix} m_{13}^{RCPG} & m_{14}^{RCPG} \end{matrix} \end{matrix} \end{matrix} \end{matrix} \right]=\left[ \begin{matrix} \begin{matrix} \begin{matrix} \begin{matrix} s_{0}^{0G\_1} & s_{0}^{0G\_2} \end{matrix} & \begin{matrix} s_{0}^{0G\_3} & s_{0}^{0G\_4} \end{matrix} \end{matrix} \\ \begin{matrix} \begin{matrix} s_{0}^{90G\_1} & s_{0}^{90G\_2} \end{matrix} & \begin{matrix} s_{0}^{90G\_3} & s_{0}^{90G\_4} \end{matrix} \end{matrix} \end{matrix} \\ \begin{matrix} \begin{matrix} \begin{matrix} s_{0}^{135G\_1} & s_{0}^{135G\_2} \end{matrix} & \begin{matrix} s_{0}^{135G\_3} & s_{0}^{135G\_4} \end{matrix} \end{matrix} \\ \begin{matrix} \begin{matrix} s_{0}^{45G\_1} & s_{0}^{45G\_2} \end{matrix} & \begin{matrix} s_{0}^{45G\_3} & s_{0}^{45G\_4} \end{matrix} \end{matrix} \end{matrix} \\ \begin{matrix} \begin{matrix} \begin{matrix} s_{0}^{LCPG\_1} & s_{0}^{LCPG\_2} \end{matrix} & \begin{matrix} s_{0}^{LCPG\_3} & s_{0}^{LCPG\_4} \end{matrix} \end{matrix} \\ \begin{matrix} \begin{matrix} s_{0}^{RCPG\_1} & s_{0}^{RCPG\_2} \end{matrix} & \begin{matrix} s_{0}^{RCPG\_3} & s_{0}^{RCPG\_4} \end{matrix} \end{matrix} \end{matrix} \end{matrix} \right]\times\left[ \begin{matrix} \begin{matrix} s_{0}^{1} \\ s_{1}^{1} \end{matrix} \\ \begin{matrix} s_{2}^{1} \\ s_{3}^{1} \end{matrix} \end{matrix}\begin{matrix} \begin{matrix} s_{0}^{2} \\ s_{1}^{2} \end{matrix} \\ \begin{matrix} s_{2}^{2} \\ s_{3}^{2} \end{matrix} \end{matrix}\begin{matrix} \begin{matrix} s_{0}^{3} \\ s_{1}^{3} \end{matrix} \\ \begin{matrix} s_{2}^{3} \\ s_{3}^{3} \end{matrix} \end{matrix}\begin{matrix} \begin{matrix} s_{0}^{4} \\ s_{1}^{4} \end{matrix} \\ \begin{matrix} s_{2}^{4} \\ s_{3}^{4} \end{matrix} \end{matrix} \right]^{-1}=I\times S^{-1}$ | (4) |
| --- | --- | --- |

To guarantee a more stable calculation of the instrument matrix, eight polarization states are used for real measurements.


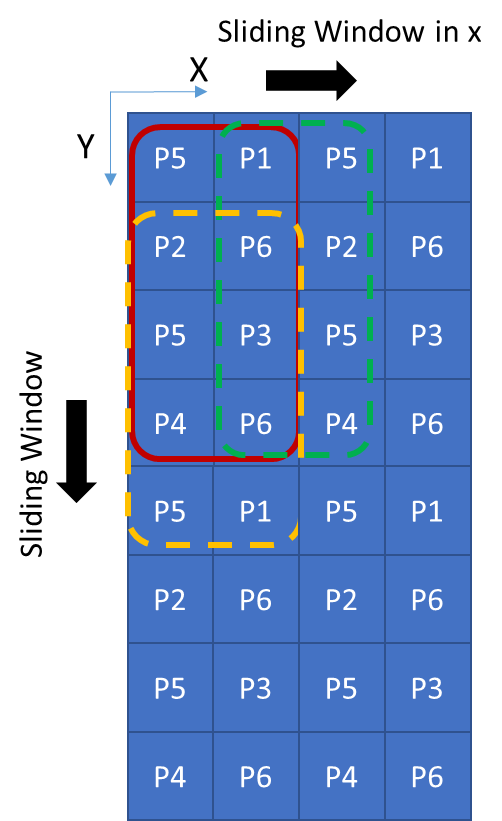


**Supplementary Figure 23. Illustration of the working principle of demosaicking during the instrument matrix calibration process.** A new intensity vector can be obtained by sliding the superpixel registry window at both the x and y direction, 1 pixel per step, because of the periodic distribution of each pixel (P1-P6).

A sliding window sampling approach is applied during instrument matrix calibration process to increase the imaging resolution, as shown in Supplementary Figure S20. A sliding window is moving along x and y directions with one pixel, i.e., metasurface filter per step. Each newly registered superpixel has a complete set of LP filters and CP filters, a correspondent instrument matrix can thus be registered using method mentioned above.

## Full Stokes polarization measurement at different incidence angle

***
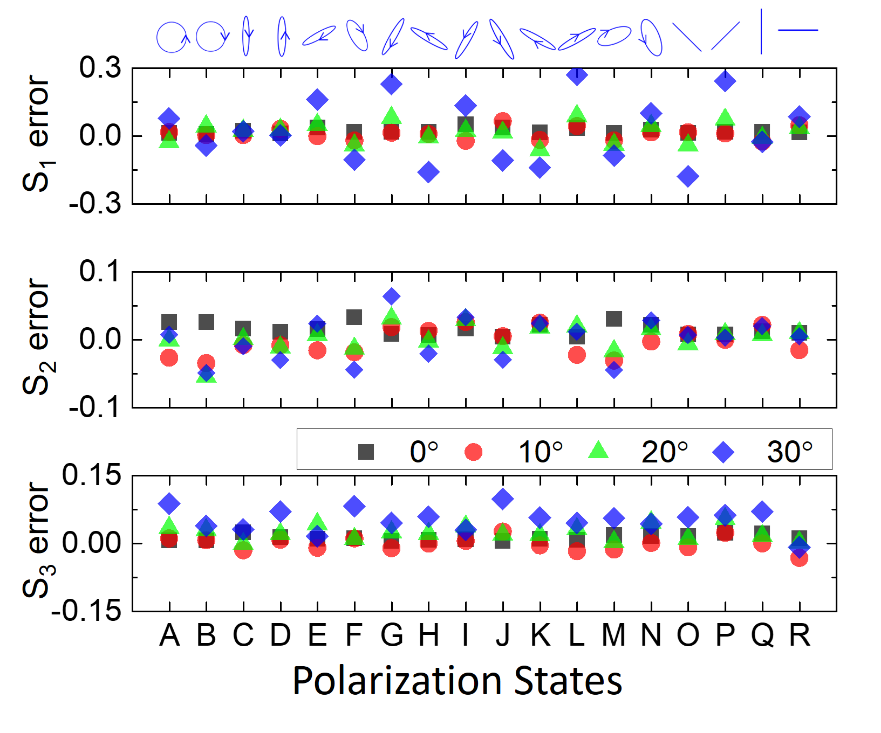
***

**Supplementary Figure 24. Full Stokes polarization measurement at different incidence angles, bandwidth 630-670 nm.**


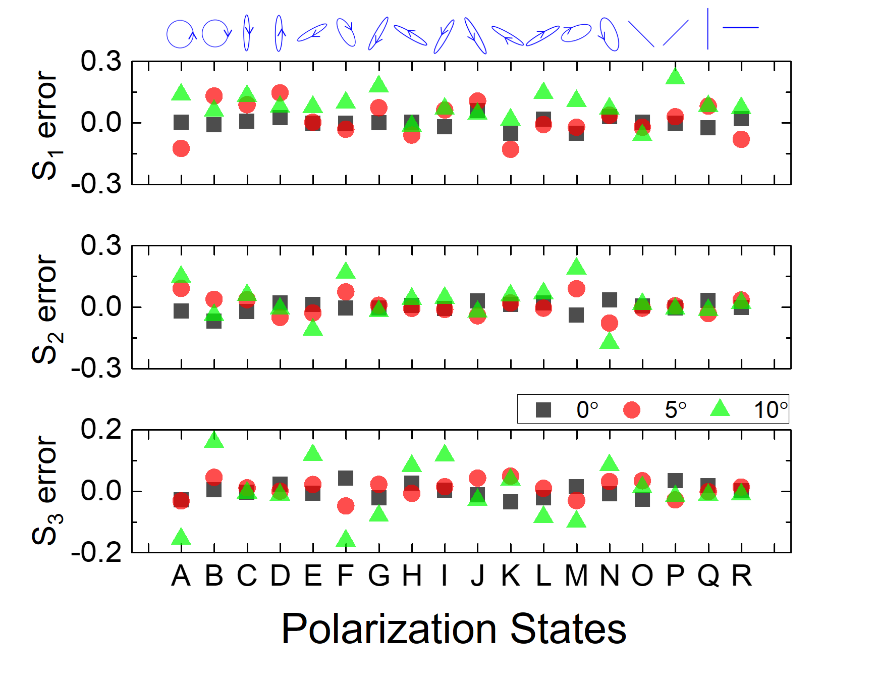


**Supplementary Figure 25. Full Stokes polarization measurement at different incidence angles, bandwidth 480-520 nm.**

**Supplementary Table 5. Table of mean absolute error and average standard deviation for 18 polarization states at different incidence angle** $\varphi$ **at red color input.**

| ***Red (630-670nm) MAE ± SD*** | | | | | | |
| --- | --- | --- | --- | --- | --- | --- |
| Name $\varphi$ | ***S_1_/S_0_*** | ***S_2_/S_0_*** | ***S_3_/S_0_*** | ***DOCP*** | ***AOP*** | ***DOLP*** |
| 0° | 1.03%±0.84% | 1.43%±0.77% | 1.99%±0.88% | 1.99%±0.88% | 0.26°±0.28° | 1.41%±0.72% |
| ±10° | 2.02%±3.97% | 1.57%±3.69% | 2.78%±4.35% | 2.78%±4.35% | 0.63°±1.24° | 1.71%±3.68% |
| *±*20° | 3.29%±3.88% | 1.82%±3.85% | 3.84%±4.72% | 3.84%±4.72% | 0.85°±1.23° | 2.28%±3.80% |
| ±30° | 9.99%±5.93% | 5.19%±5.36% | 17.51%±7.14% | 17.51%±7.14% | 3.07°±1.54° | 6.41%±6.16% |

**Supplementary Table 6. Table of mean absolute error and average standard deviation for 18 polarization states at different incidence angle** $\varphi$ **at green color input.**

| ***Green (480-520nm) MAE± SD*** | | | | | | |
| --- | --- | --- | --- | --- | --- | --- |
| Name  $\varphi$ | ***S_1_/S_0_*** | ***S2/S_0_*** | ***S_3_/S_0_*** | ***DOCP*** | ***AOP*** | ***DOLP*** |
| 0° | 1.84%±0.85% | 1.93%±0.76% | 1.79%±2.38% | 1.79%±2.38% | 0.78°±0.25° | 1.26%±0.79% |
| ±5° | 1.88%±3.21% | 2.03%±3.09% | 4.44%±11.38% | 4.44%±11.38% | 0.85°±1.07° | 1.04%±2.97% |
| ±10° | 2.17%±3.54% | 3.71%±3.49% | 22.75%±15.39% | 22.75%±15.39% | 0.78°±1.15° | 3.52%±3.34% |


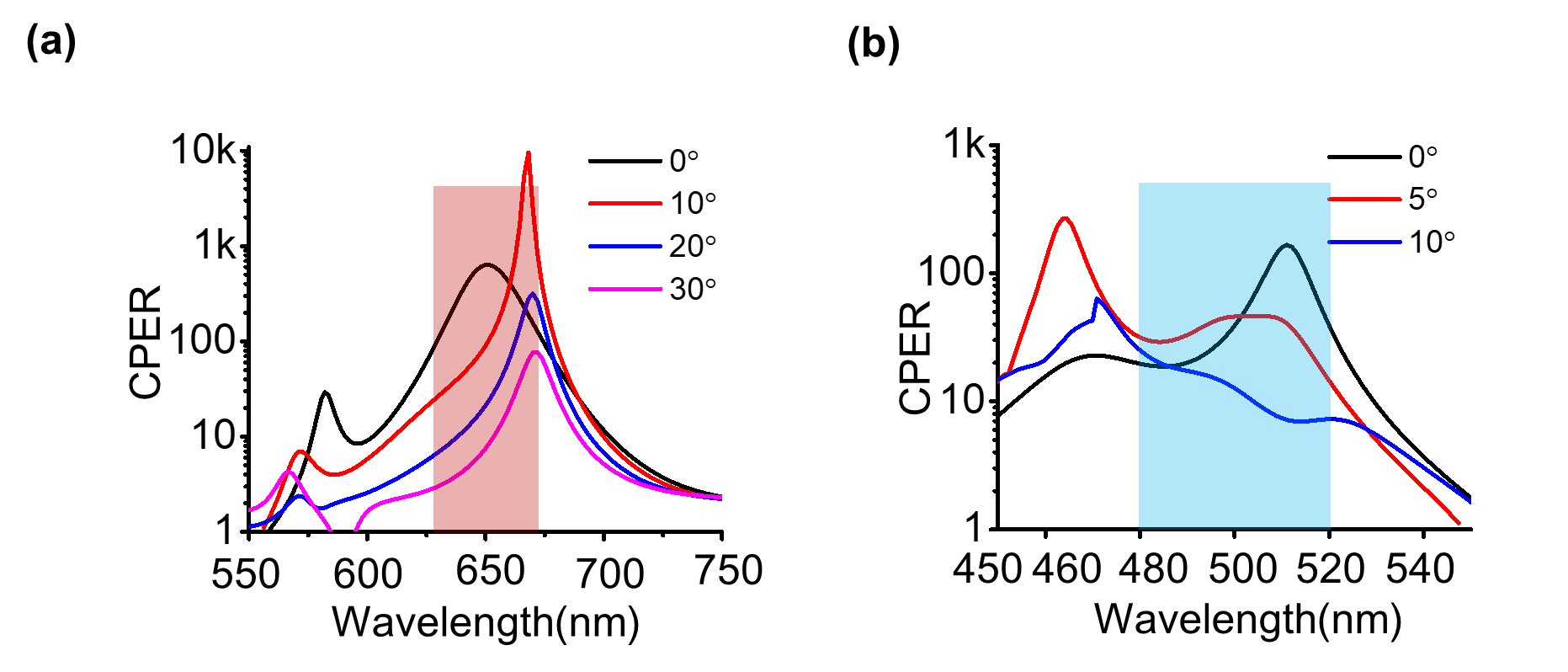


**Supplementary Figure 26. FDTD simulated CPER of chiral metasurface of under oblique incidence**. **The dimensions of Si gratings, spacer layer thickness and VCDGs are the same as presented in Fig.2d.** (a,b).FDTD simulation at 550 nm~750 nm and 450 nm~550 nm respectively.


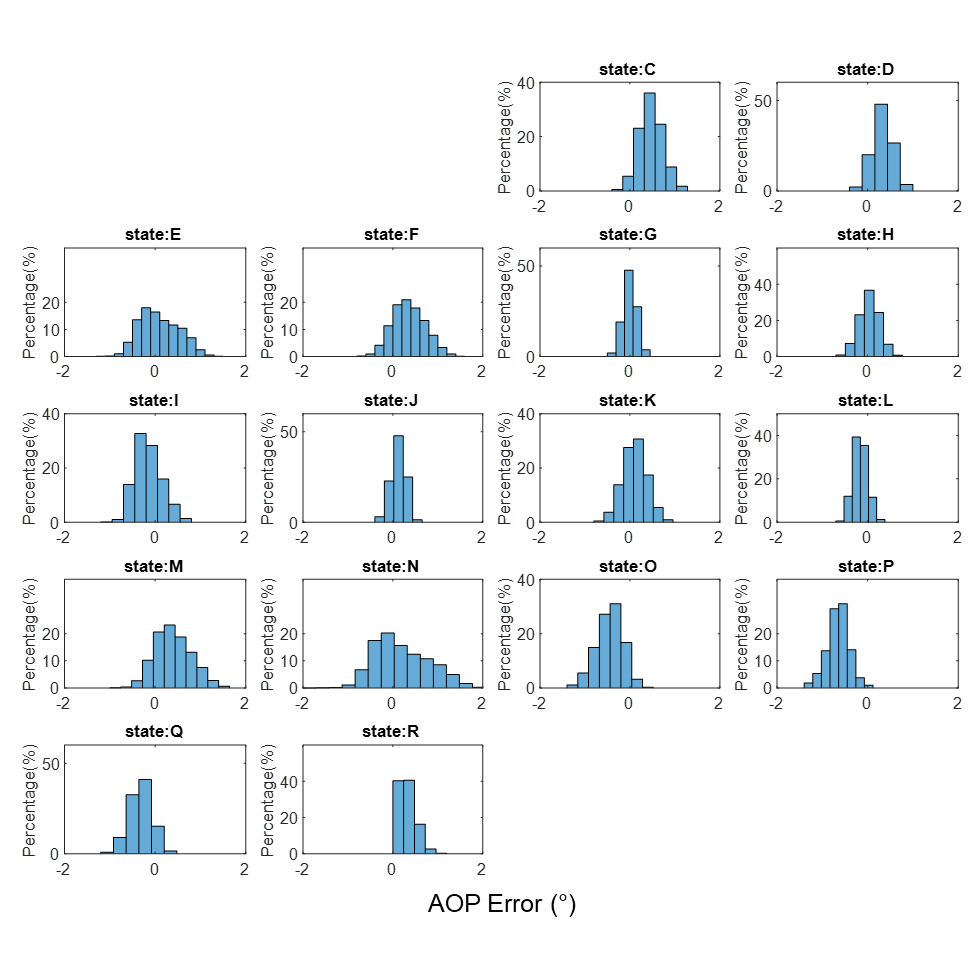


**Supplementary Figure 27. AOP measurement error distribution of MPFA under red color input,** $\varphi=0$°.


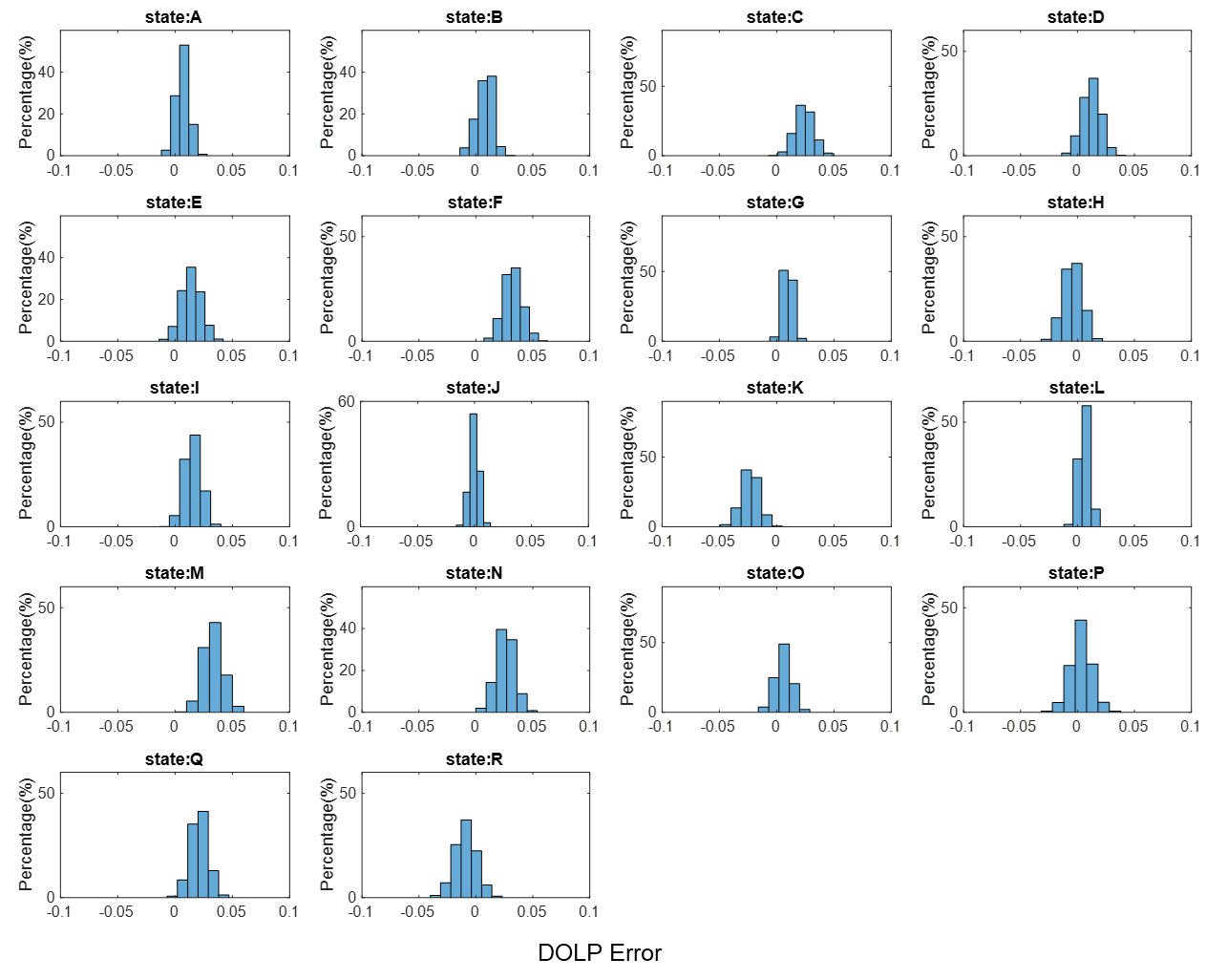


**Supplementary Figure 28. DOLP measurement error distribution of MPFA under red color input,** $\varphi=0$°.


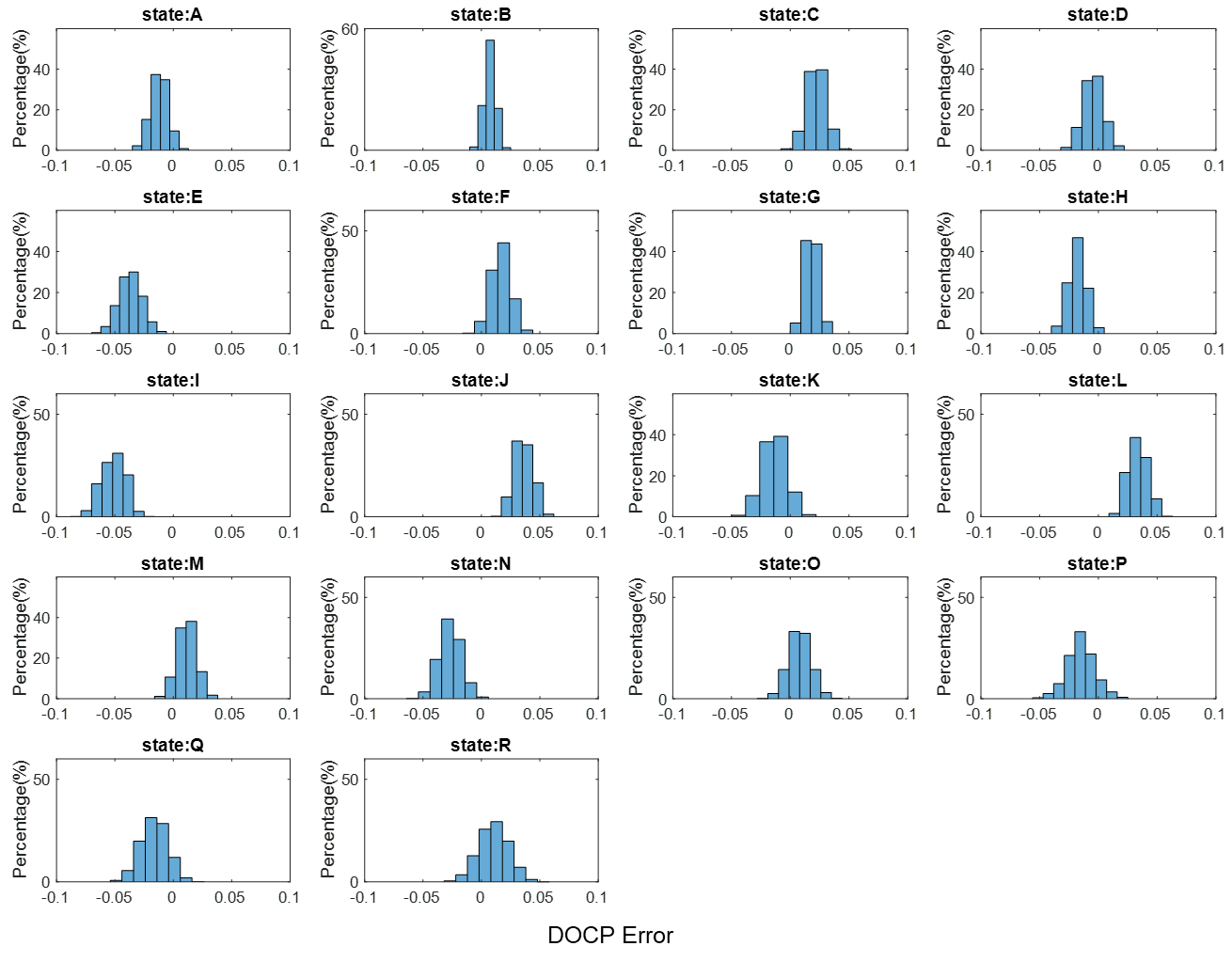


**Supplementary Figure 29. DOCP measurement error distribution of MPFA under red color input,** $\varphi=0$°.

***
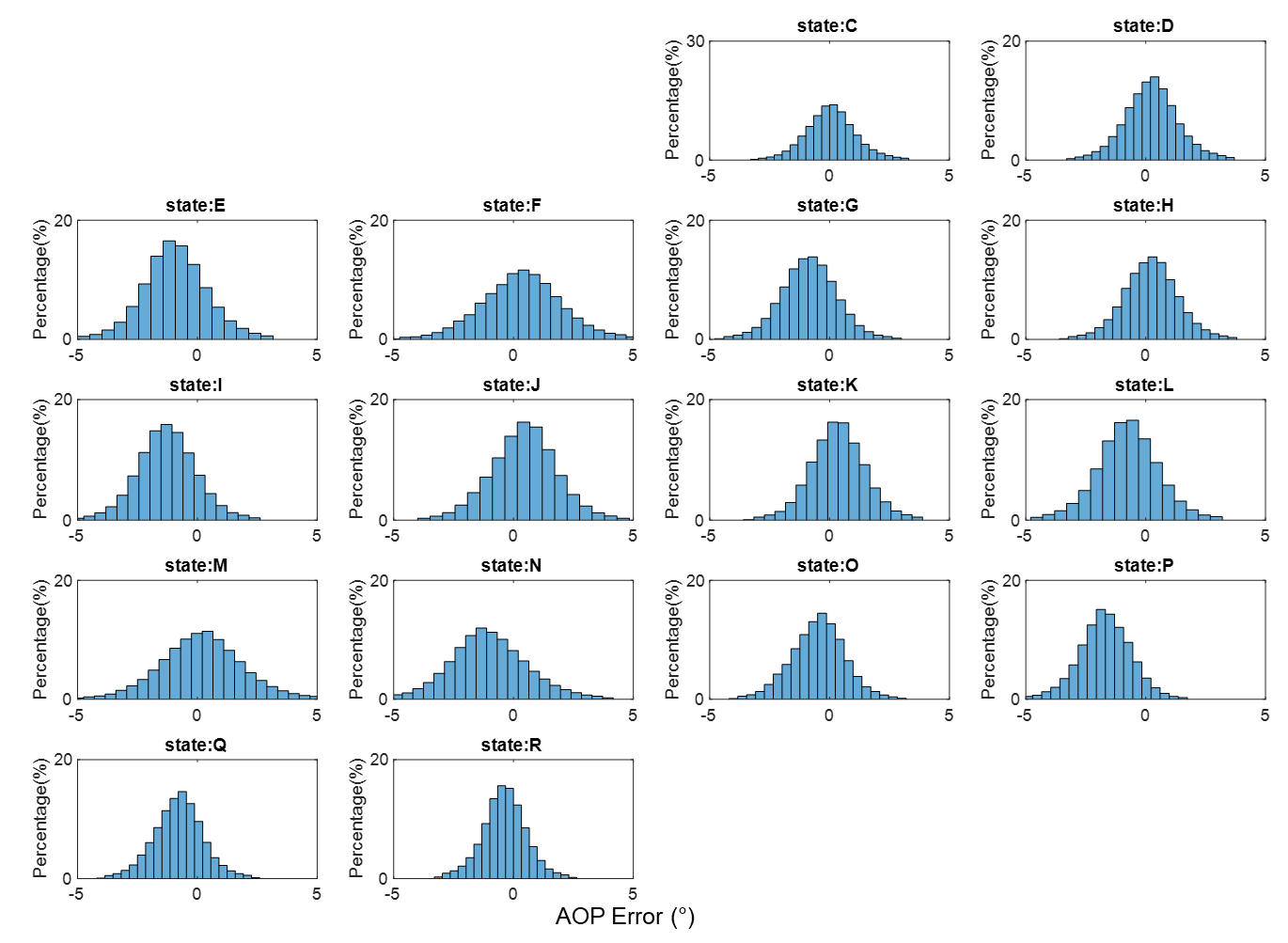
***

**Supplementary Figure 30. AOP measurement error distribution of MPFA under red color input,** $\varphi=10$°.

***
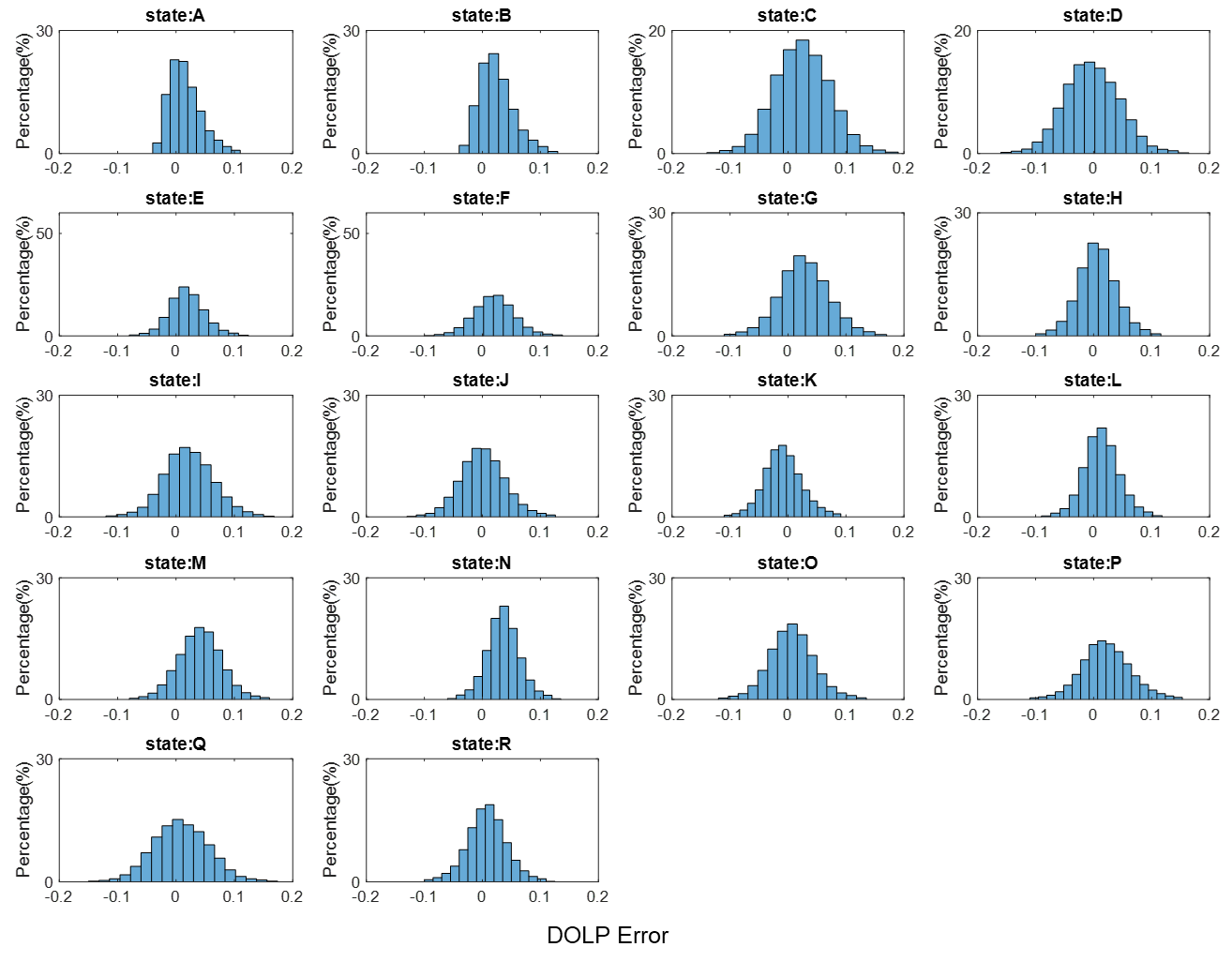
***

**Supplementary Figure 31. DOLP measurement error distribution of MPFA under red color input,** $\varphi=10$°

***
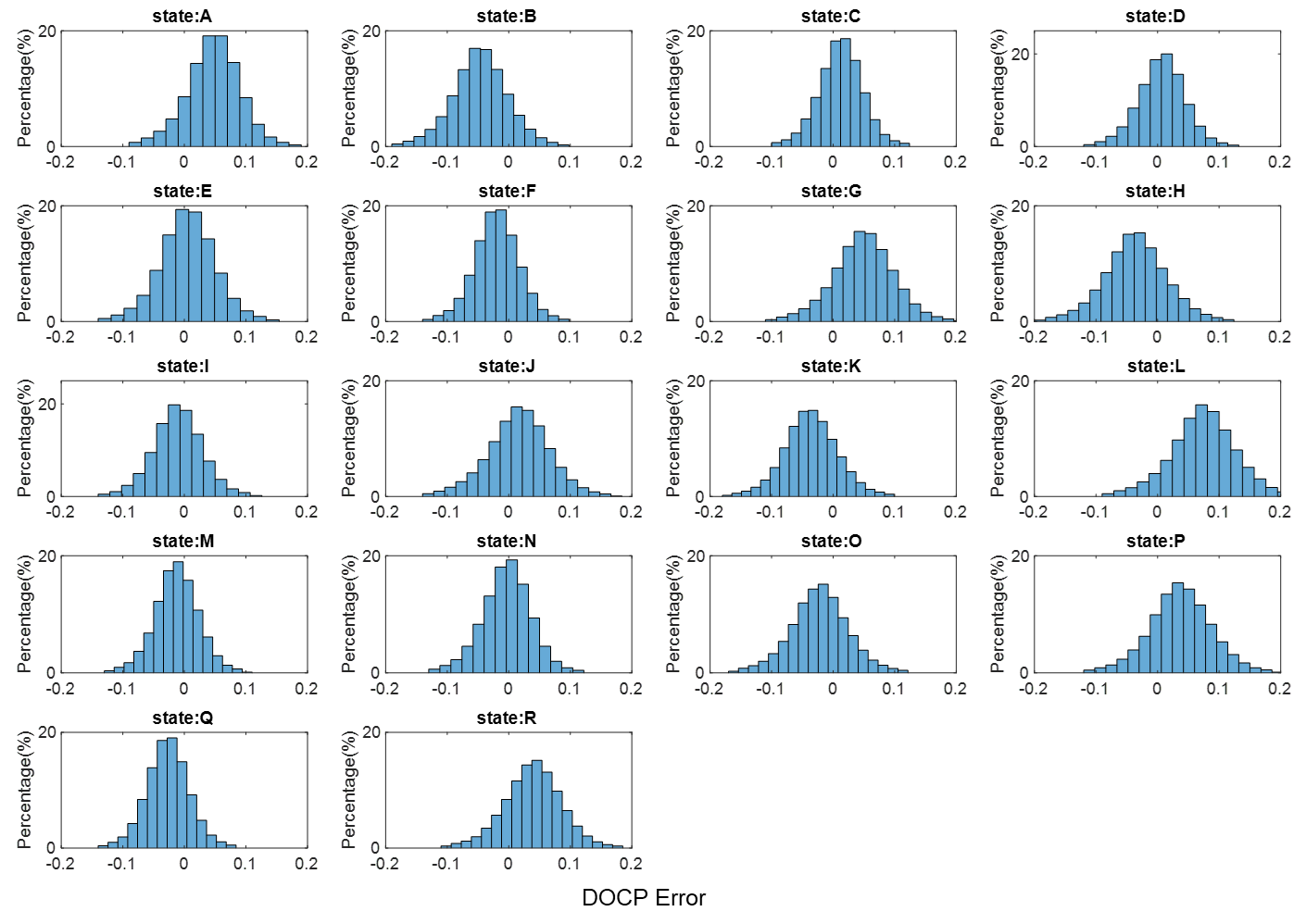
***

**Supplementary Figure 32. DOCP measurement error distribution of MPFA under red color input,** $\varphi=10$°

***
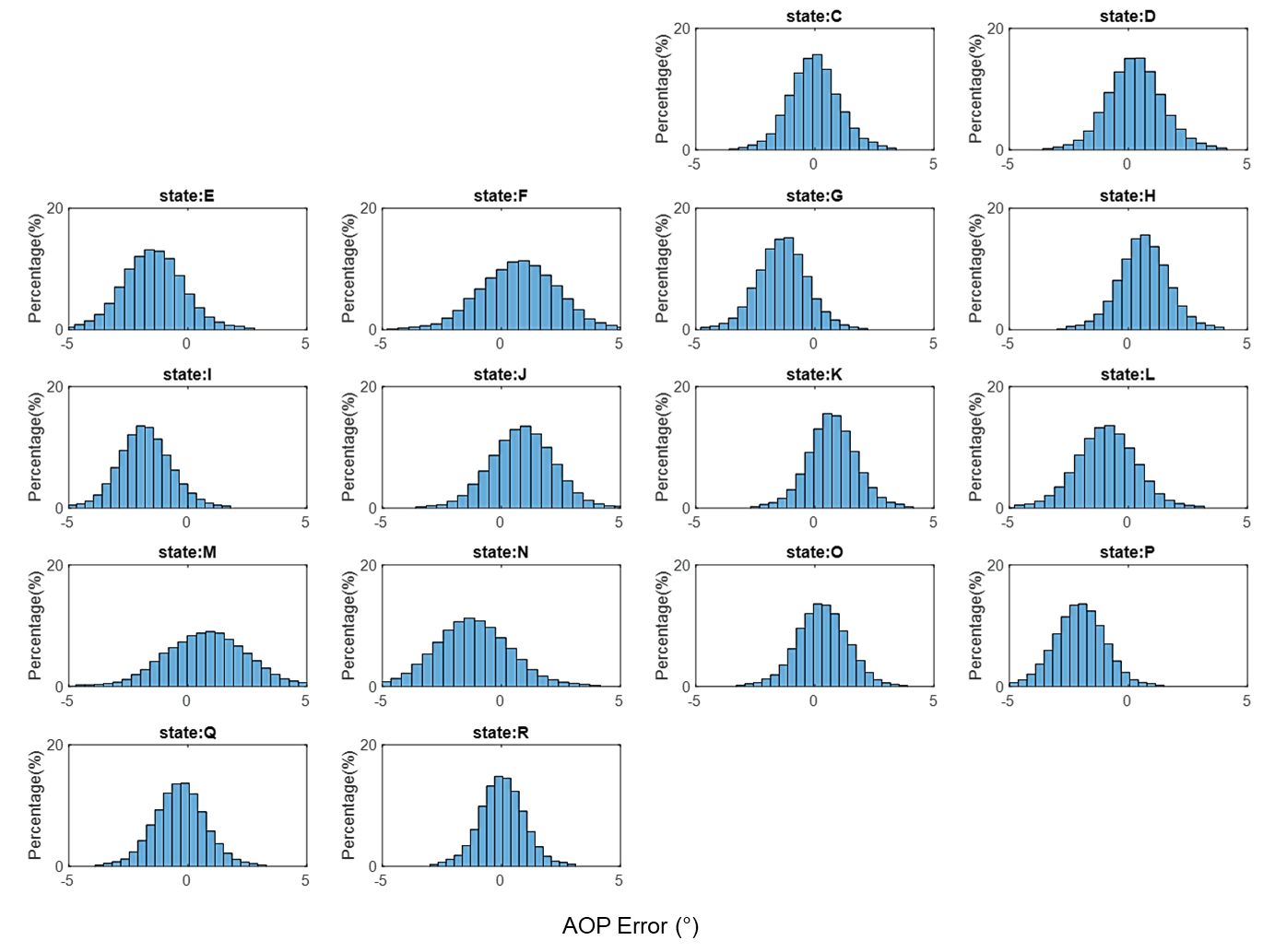
*Supplementary Figure 33. AOP measurement error distribution of MPFA under red color input,** $\varphi=20$°.

***
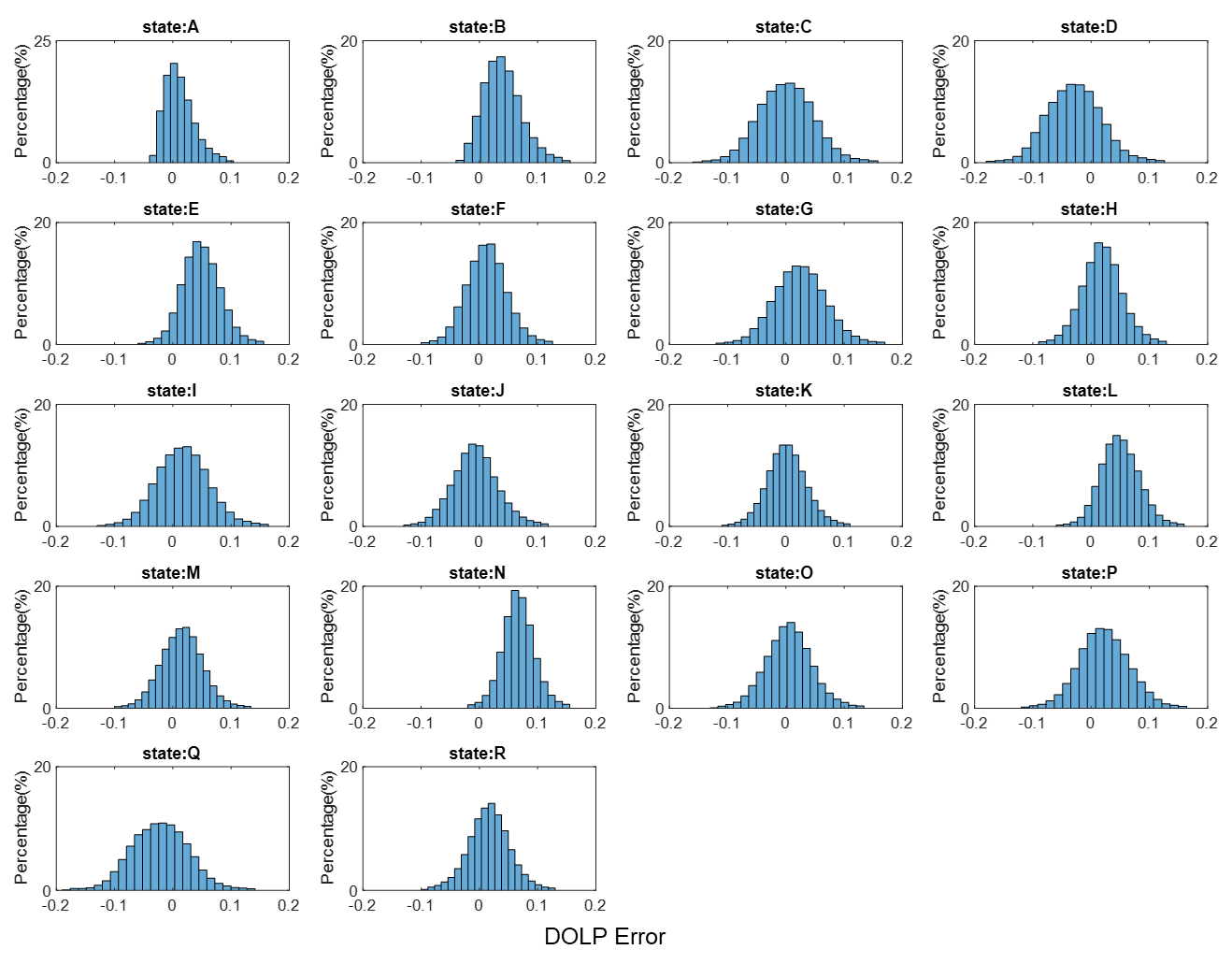
***

**Supplementary Figure 34. DOLP measurement error distribution of MPFA under red color input,** $\varphi=20$°

***
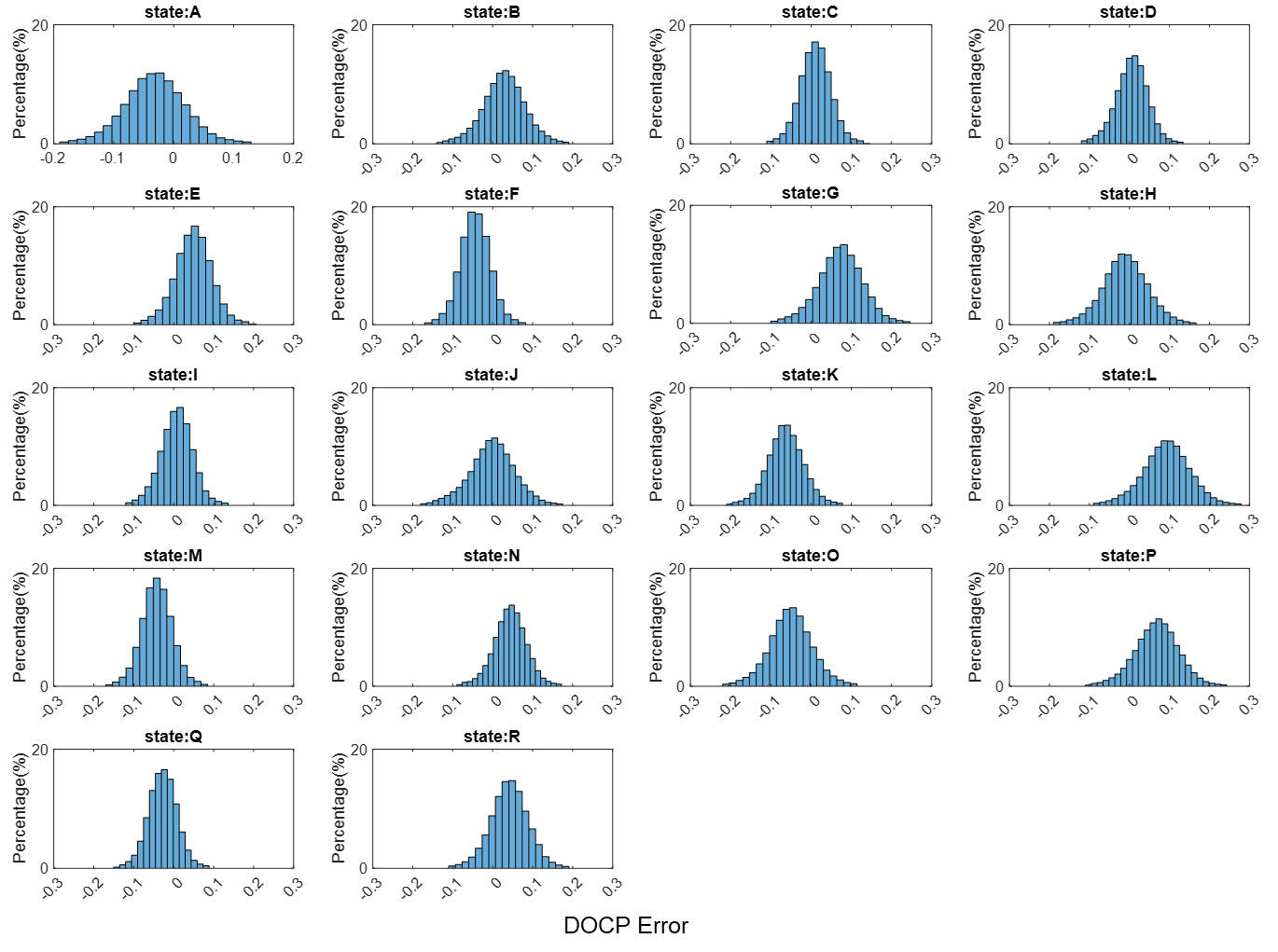
***

**Supplementary Figure 35. DOCP measurement error distribution of MPFA under red color input,** $\varphi=20$°

***
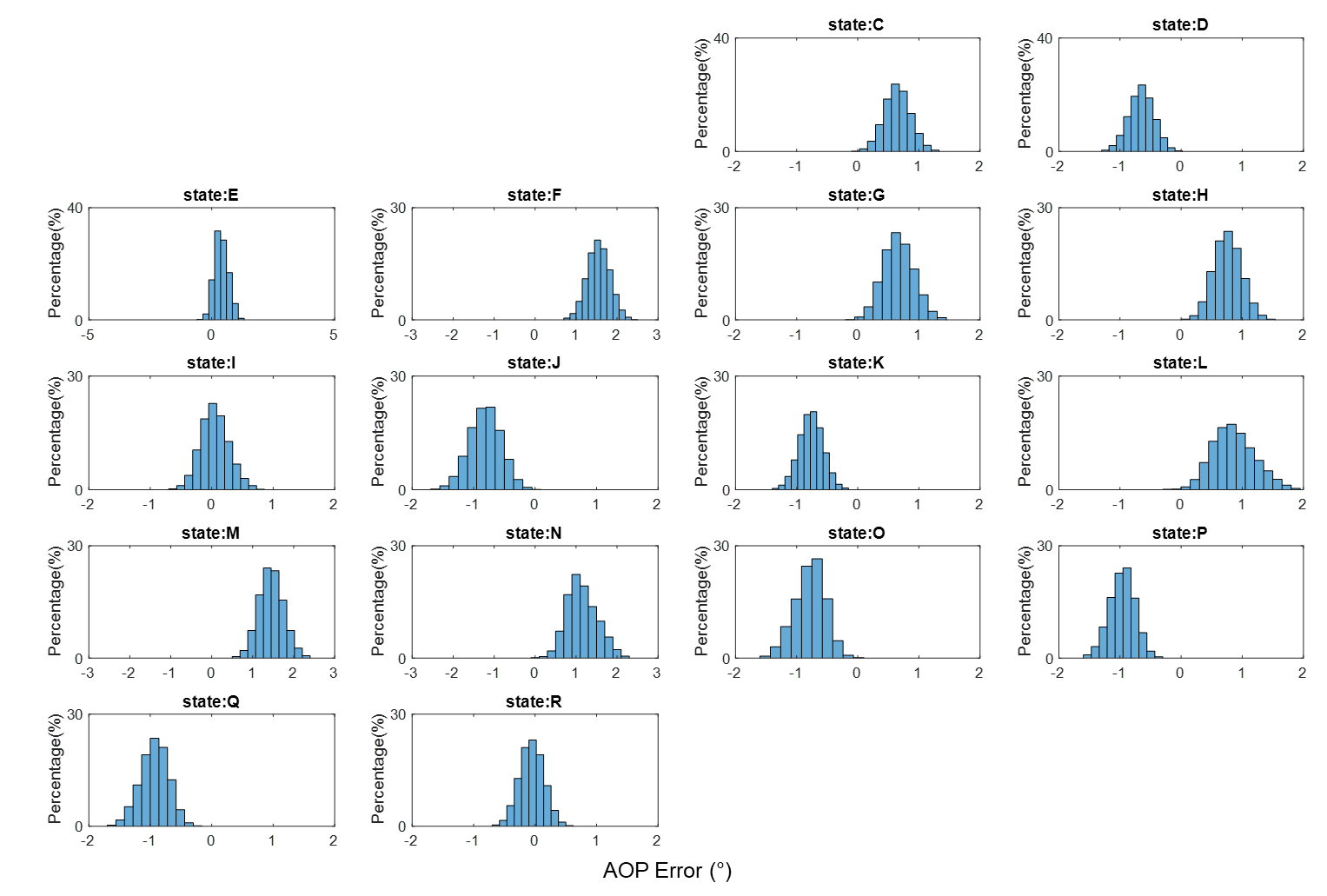
***

**Supplementary Figure 36. AOP measurement error distribution of MPFA under green color input,** $\varphi=0$°.

***
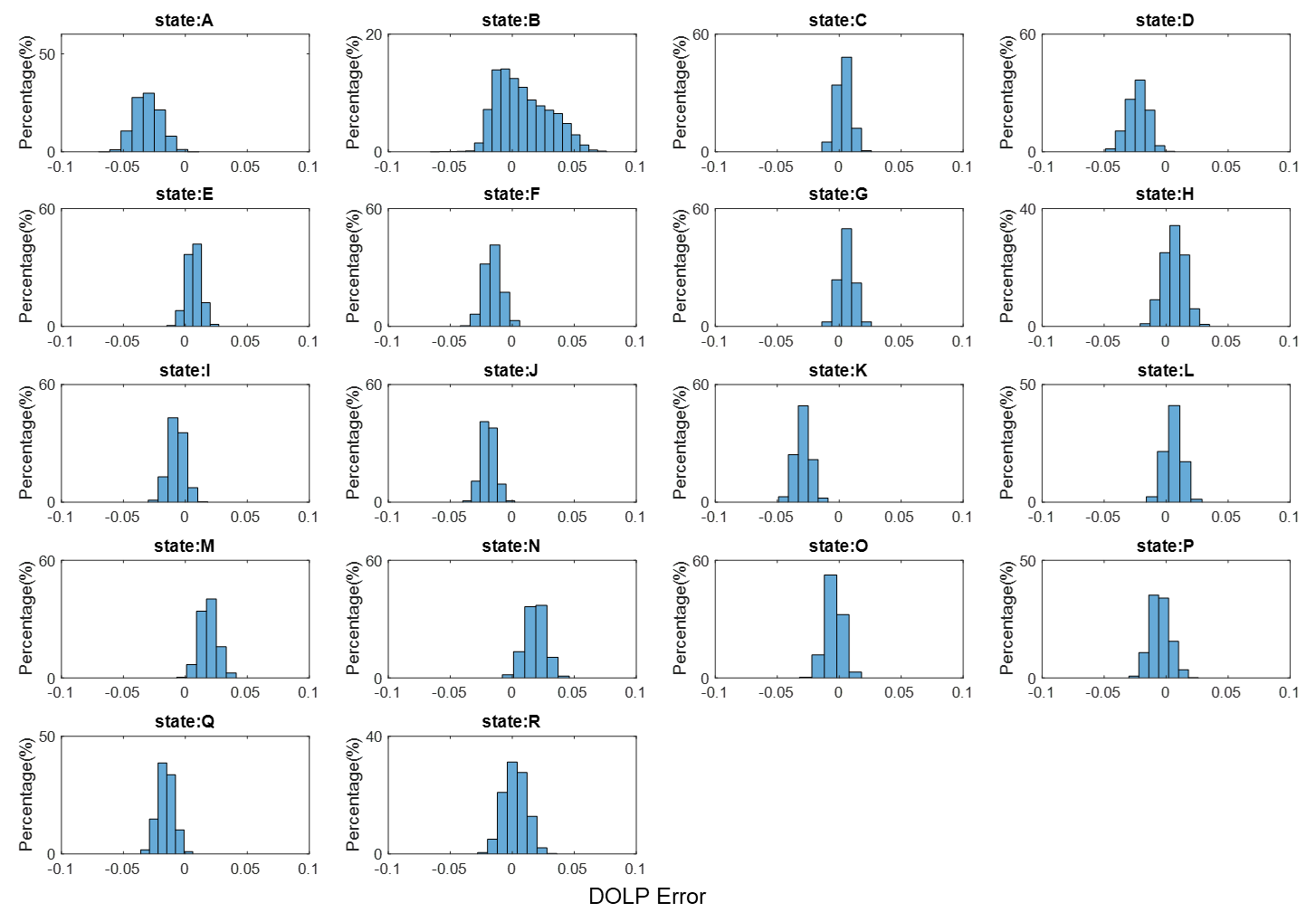
***

**Supplementary Figure 37. DOLP measurement error distribution of MPFA under green color input,** $\varphi=0$°

***
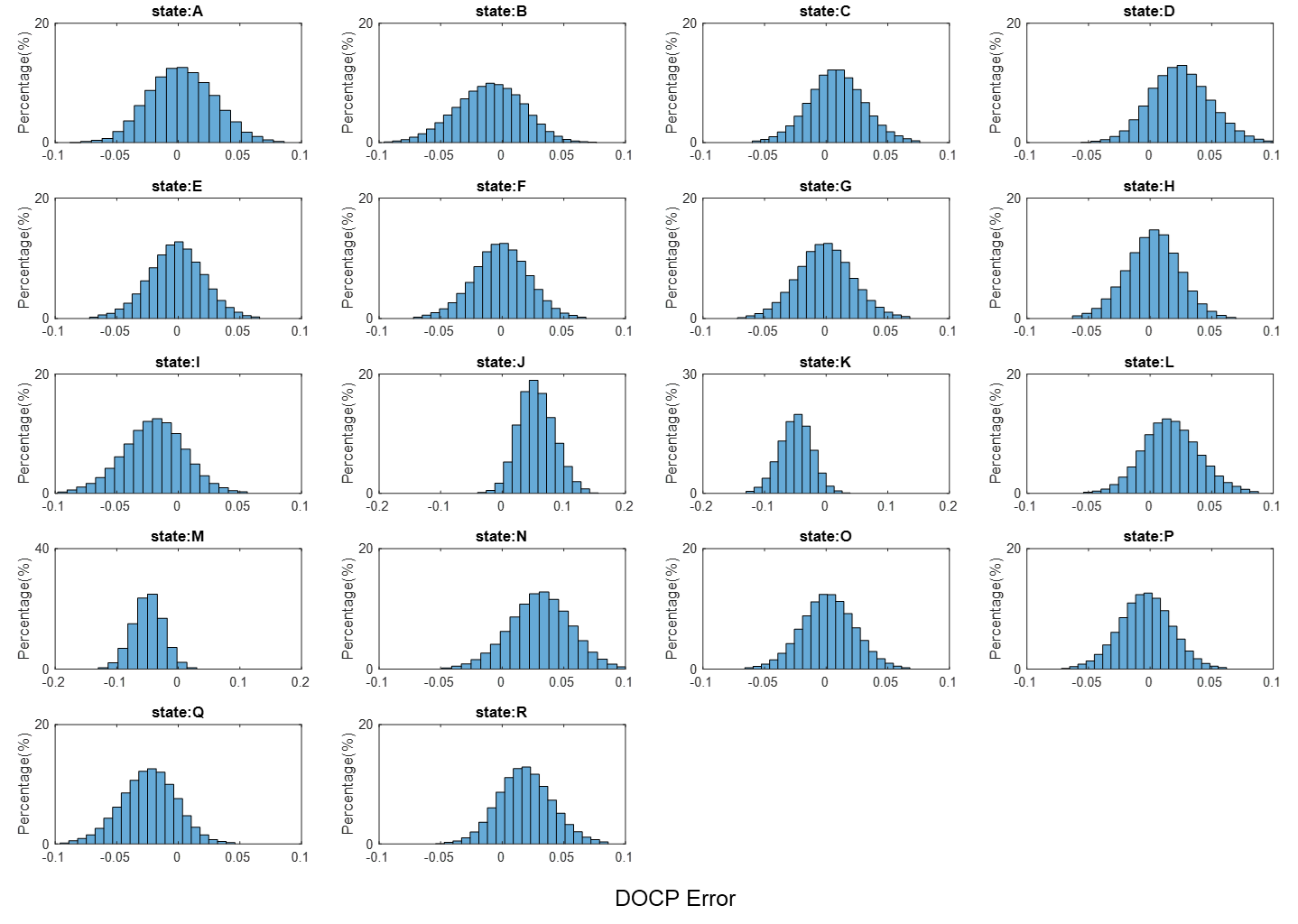
***

**Supplementary Figure 38. DOCP measurement error distribution of MPFA under green color input,** $\varphi=0$°

***
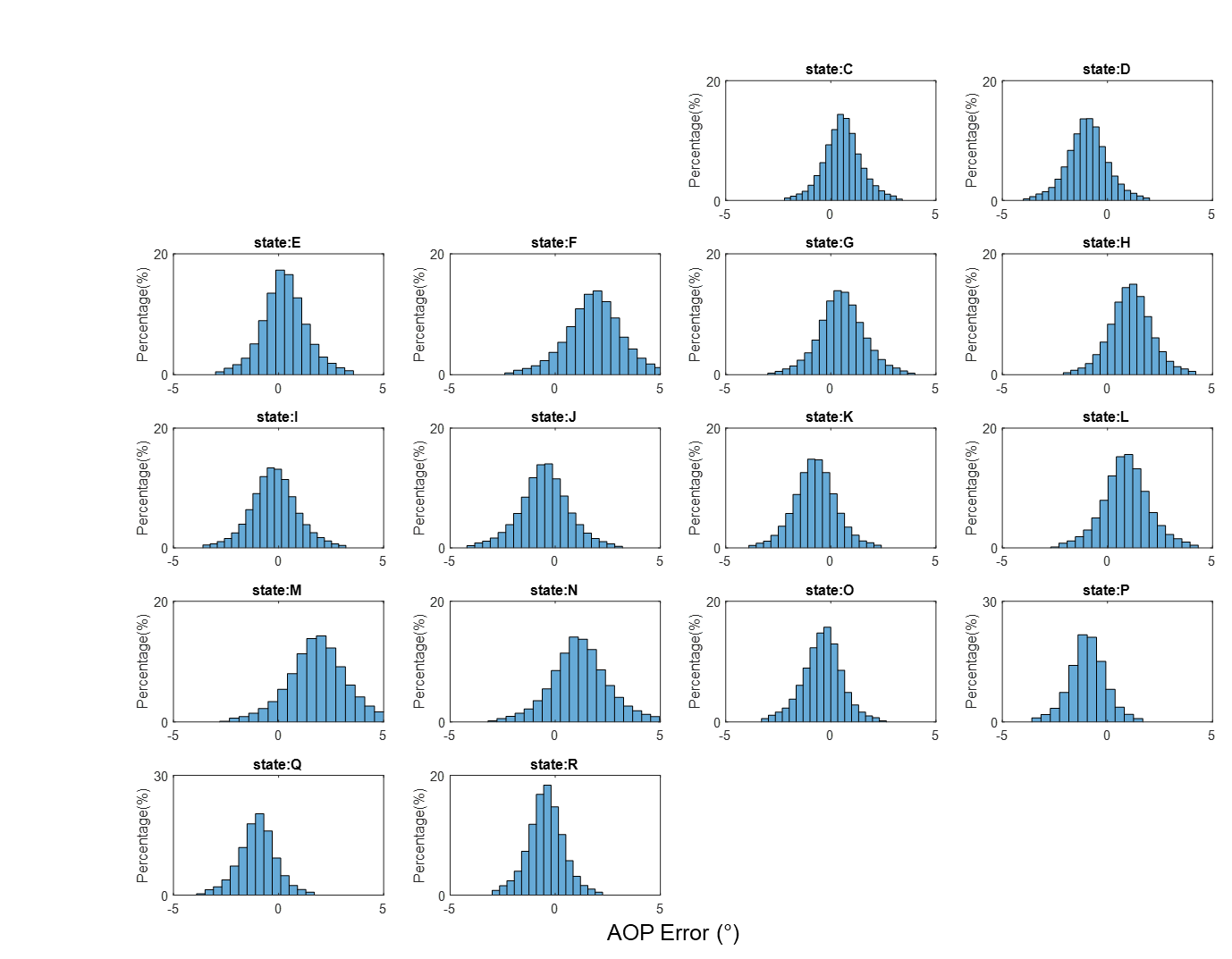
***

**Supplementary Figure 39. AOP measurement error distribution of MPFA under green color input,** $\varphi=5$°.

***
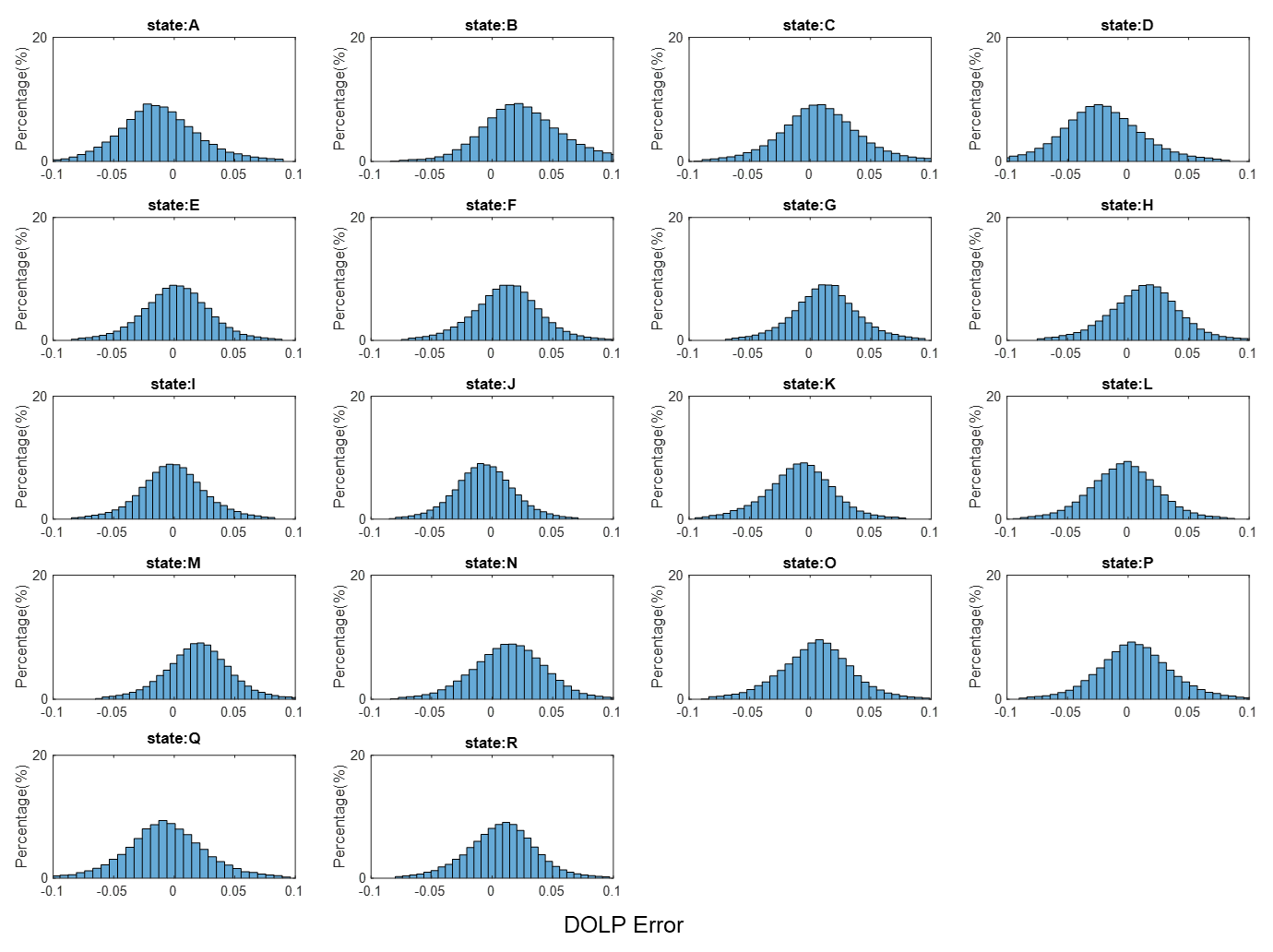
***

**Supplementary Figure 40. DOLP measurement error distribution of MPFA under green color input,** $\varphi=5$°

***
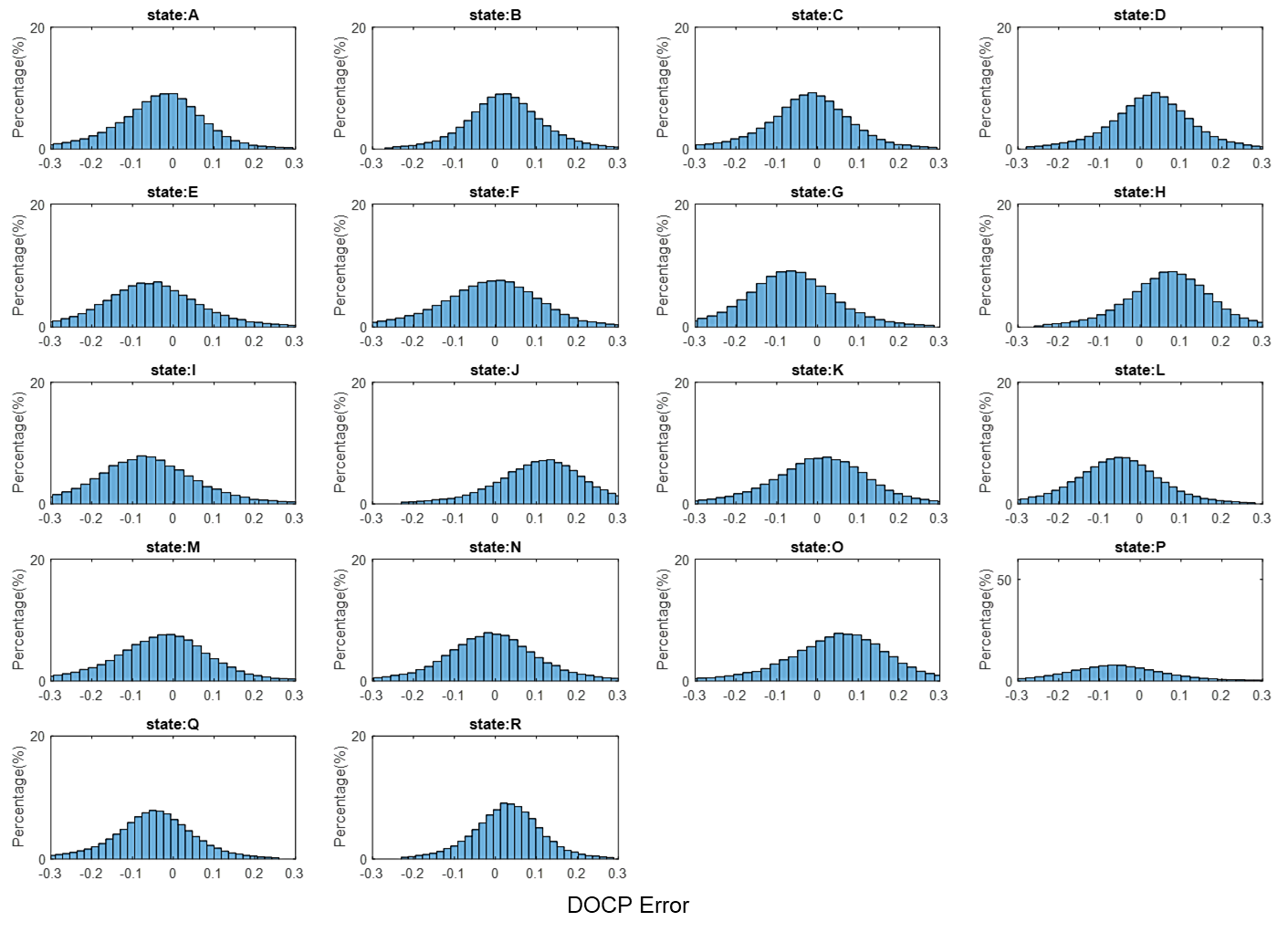
***

**Supplementary Figure 41. DOCP measurement error distribution of MPFA under green color input,** $\varphi=5$°


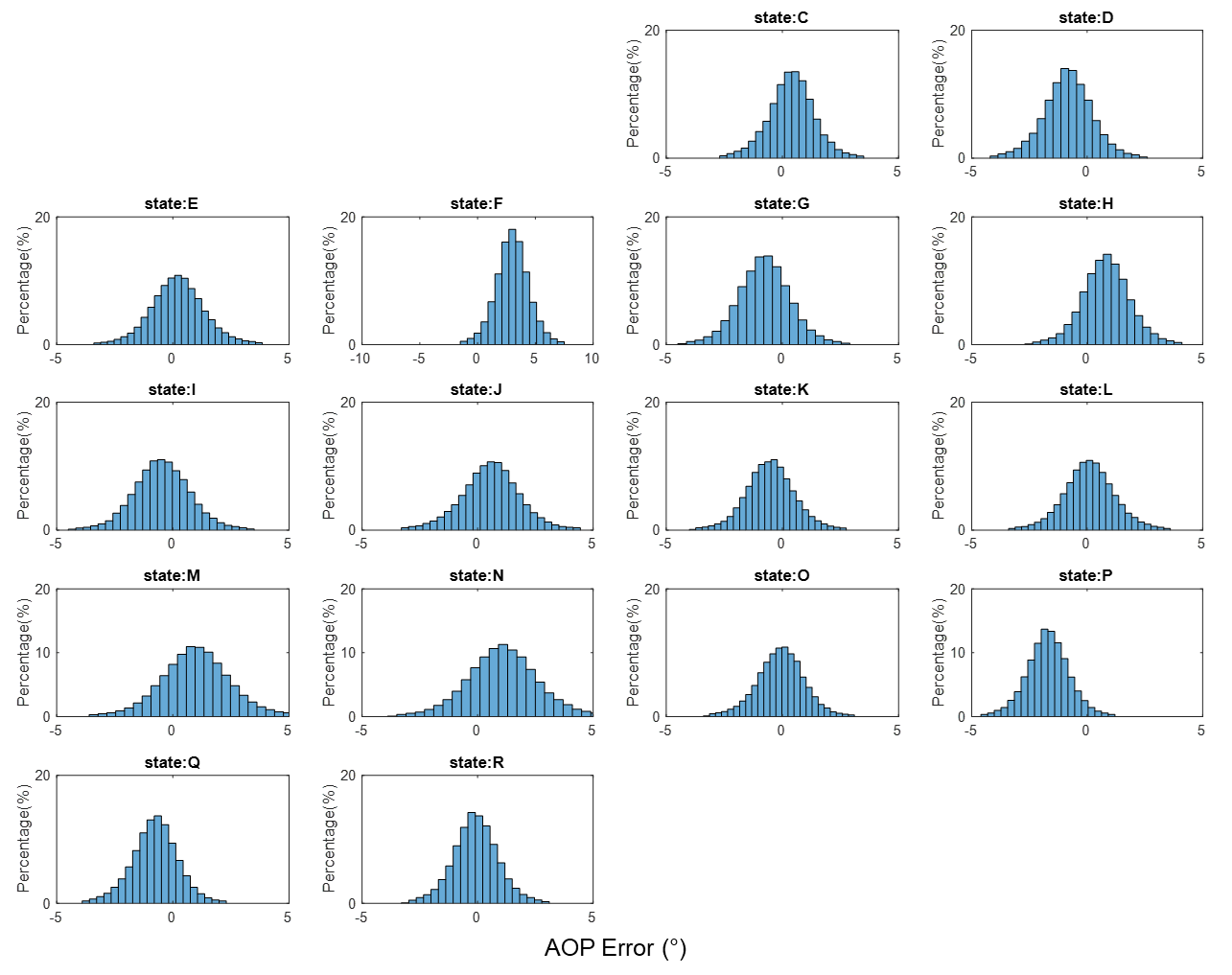


**Supplementary Figure 42. AOP measurement error distribution of MPFA under green color input,** $\varphi=10$°.

***
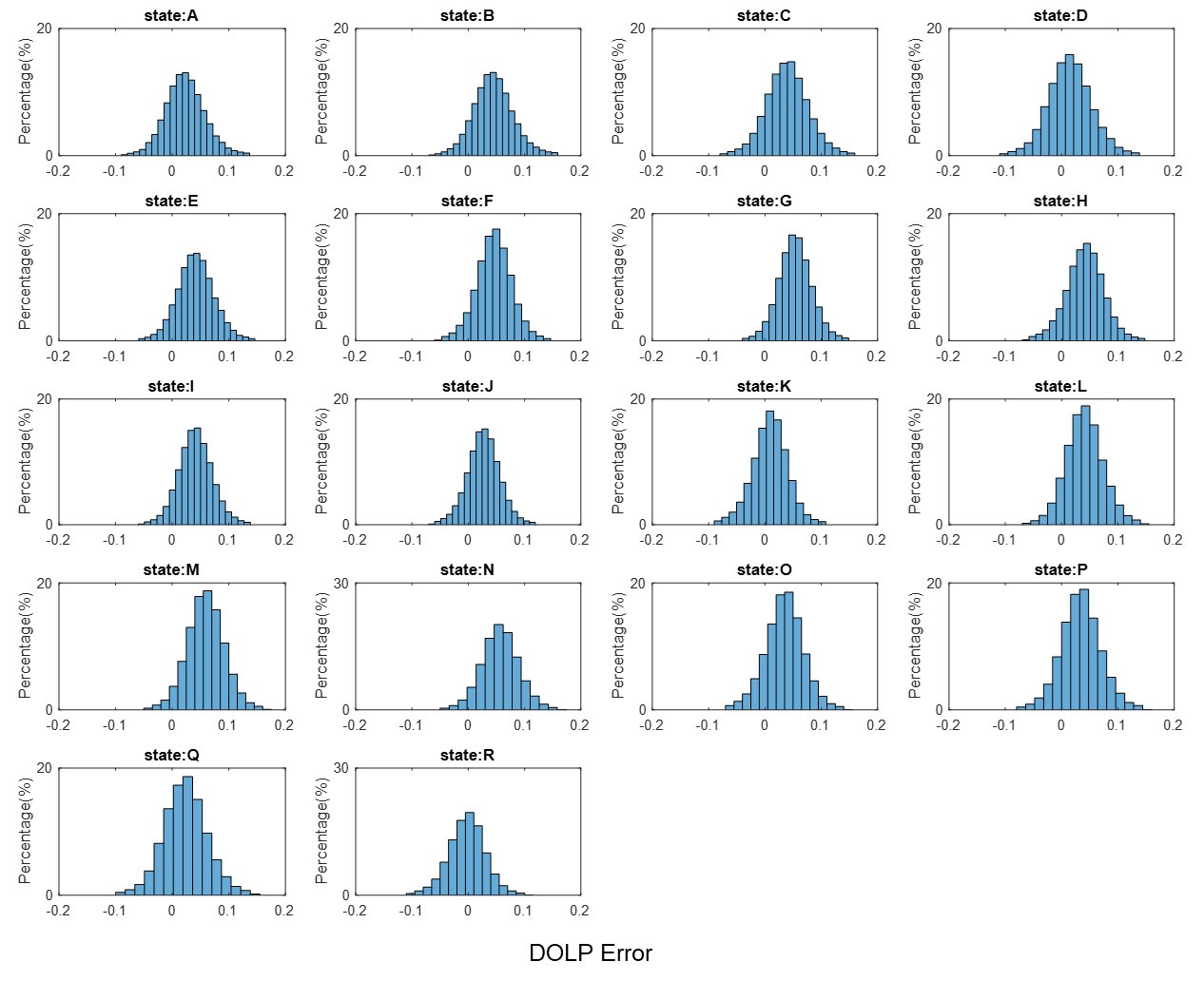
***

**Supplementary Figure 43. DOLP measurement error distribution of MPFA under green color input,** $\varphi=10^{\circ}$

***
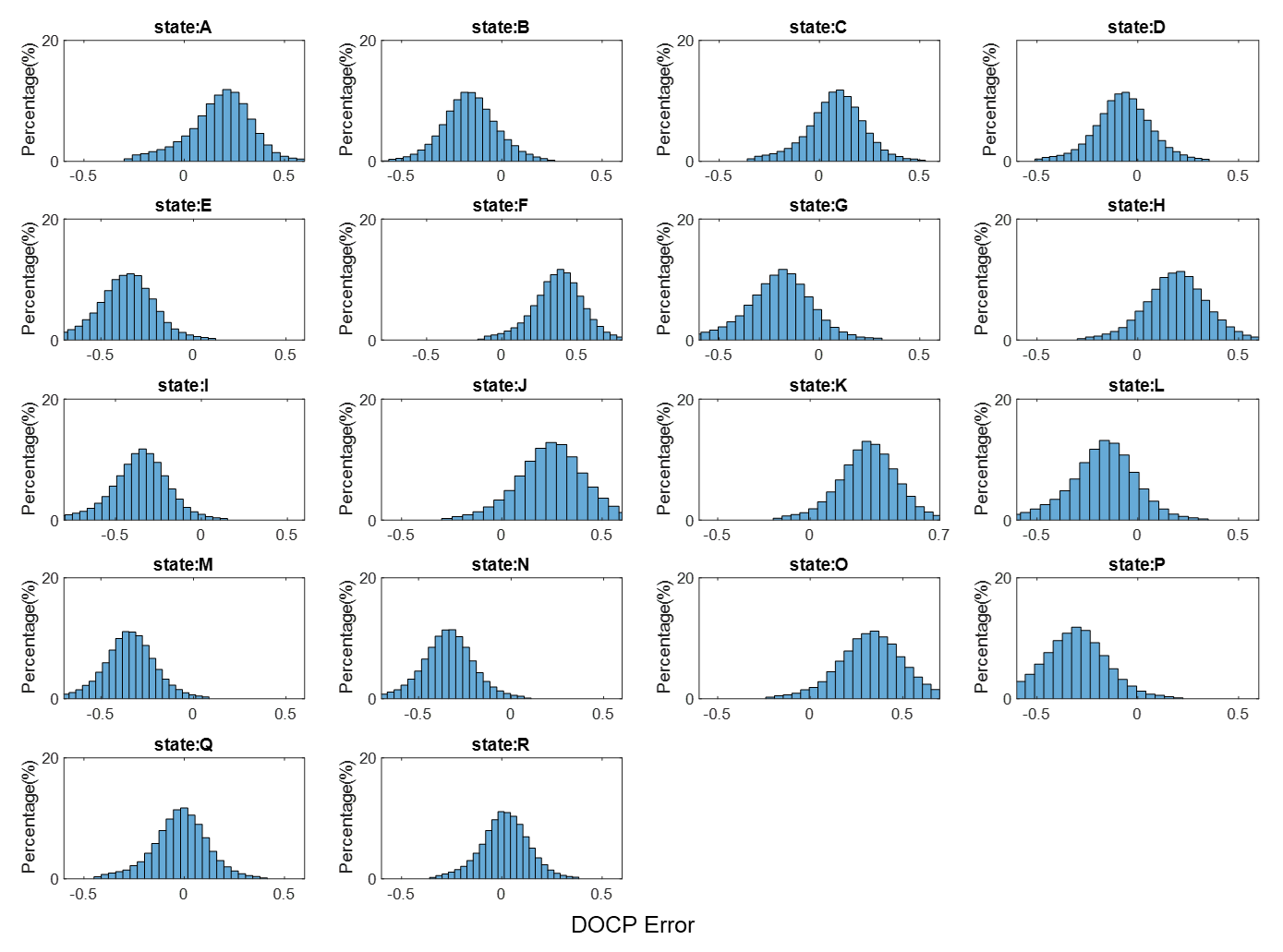
***

**Supplementary Figure 44. DOCP measurement error distribution of MPFA under green color input,** $\varphi=10^{\circ}$

## Full Stokes polarization imaging of ASU logo with polarization information

***
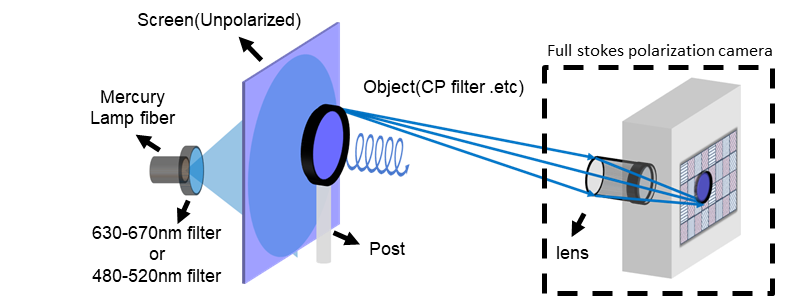
***

**Supplementary Figure 45. Illustration of full Stokes polarization imaging setup.**

Supplementary information accompanies the manuscript on the Light: Science & Applications website ([http://www.nature.com/lsa](https://urldefense.com/v3/__http:/www.nature.com/lsa__;!!IKRxdwAv5BmarQ!f2KAXTU6MEzLYaGeS2V0FrMfLrudtUVNBNYeIoCO1O2O7SsiFZGr2PXUnbdsK938ZxnmHR-Rr0zWEl_mCn0$))

**Supplementary References**

1. Thorlabs. *PAX1000VIS/M - Polarimeter*. Available from: <https://www.thorlabs.com/thorproduct.cfm?partnumber=PAX1000VIS/M>.

2. Vedel, M., S. Breugnot, and N. Lechocinski. *Full Stokes polarization imaging camera*. in *Polarization Science and Remote Sensing V*. 2011. International Society for Optics and Photonics.

3. Sony. *Sony Polarsens* 2022; Available from: <https://www.sony-semicon.com/en/technology/industry/polarsens.html>.

4. Tu, X., et al., *Division of focal plane red–green–blue full-Stokes imaging polarimeter.* Applied optics, 2020. **59**(22): p. G33-G40.

5. Myhre, G., et al., *Liquid crystal polymer full-stokes division of focal plane polarimeter.* Optics express, 2012. **20**(25): p. 27393-27409.

6. Zhao, X., et al., *Liquid-crystal micropolarimeter array for full Stokes polarization imaging in visible spectrum.* 2010. **18**(17): p. 17776-17787.

7. Rubin, N.A., et al., *Matrix Fourier optics enables a compact full-Stokes polarization camera.* Science, 2019. **365**(6448).

8. Ren, Y., et al., *Full‐Stokes Polarimetry for Visible Light Enabled by an All‐Dielectric Metasurface.* 2022: p. 2100373.

9. Yang, Z., et al., *Generalized Hartmann-Shack array of dielectric metalens sub-arrays for polarimetric beam profiling.* Nature communications, 2018. **9**(1): p. 1-7.

10. Arbabi, E., et al., *Full-Stokes imaging polarimetry using dielectric metasurfaces.* Acs Photonics, 2018. **5**(8): p. 3132-3140.

11. Bai, J., J. Zuo, and Y. Yao. *Mid-Infrared Chip-Integrated Full-Stokes Polarimeter Array Based on Plasmonic Metasurfaces*. in *2022 Conference on Lasers and Electro-Optics (CLEO)*. 2022. IEEE.

12. Jung, M., et al., *Polarimetry using graphene-integrated anisotropic metasurfaces.* ACS Photonics, 2018. **5**(11): p. 4283-4288.

13. Li, L., et al., *Monolithic Full-Stokes Near-Infrared Polarimetry with Chiral Plasmonic Metasurface Integrated Graphene–Silicon Photodetector.* 2020. **14**(12): p. 16634-16642.

14. Bai, J. and Y.J.A.n. Yao, *Highly efficient anisotropic chiral plasmonic metamaterials for polarization conversion and detection.* 2021. **15**(9): p. 14263-14274.

15. Lee, K., et al., *Ultracompact broadband plasmonic polarimeter.* 2018. **12**(3): p. 1700297.

16. Bai, J., et al., *Chip-integrated plasmonic flat optics for mid-infrared full-Stokes polarization detection.* 2019. **7**(9): p. 1051-1060.

17. Afshinmanesh, F., et al., *Measurement of the polarization state of light using an integrated plasmonic polarimeter.* 2012. **1**(2): p. 125-129.

18. Liang, Y., et al., *Full-stokes polarization perfect absorption with diatomic metasurfaces.* 2021. **21**(2): p. 1090-1095.

19. Basiri, A., et al., *Nature-inspired chiral metasurfaces for circular polarization detection and full-Stokes polarimetric measurements.* 2019. **8**(1): p. 1-11.

20. Shah, Y.D., et al., *An All-Dielectric Metasurface Polarimeter.* ACS Photonics, 2022. **9**(10): p. 3245-3252.
